# Thiazole–Chalcone Hybrids as Prospective Antitubercular and Antiproliferative Agents: Design, Synthesis, Biological, Molecular Docking Studies and In Silico ADME Evaluation

**DOI:** 10.3390/molecules26102847

**Published:** 2021-05-11

**Authors:** Ashok Babu Kasetti, Indrajeet Singhvi, Ravindra Nagasuri, Richie R. Bhandare, Afzal B. Shaik

**Affiliations:** 1 Research Scholar, Faculty of Pharmacy, Pacific Academy of Higher Education and Research University, Pacific University, Udaipur 313003, India; 2 Dr. Samuel George Institute of Pharmaceutical Sciences, Markapuram, Andhra Pradesh 523316, India; 3 Faculty of Pharmacy, Pacific Academy of Higher Education and Research University, Pacific University, Udaipur 313003, India; indrajeetsinghvi@yahoo.com; 4 A.M. Reddy Memorial College of Pharmacy, Narasaraopeta, Andhra Pradesh 523316, India; nravi1965@gmail.com; 5 Department of Pharmaceutical Sciences, College of Pharmacy & Health Sciences, Ajman University, Ajman P.O. Box 346, United Arab Emirates; 6 Center of Medical and Bio-Allied Health Sciences Research, Ajman University, Ajman P.O. Box 346, United Arab Emirates; 7 Department of Pharmaceutical Chemistry, Vignan Pharmacy College, Vadlamudi, Guntur, Andhra Pradesh 522213, India

**Keywords:** thiazole, chalcone, antiproliferative activity, antitubercular activity, cytotoxic activity, AutoDock, SwissADME

## Abstract

Compounds bearing thiazole and chalcone pharmacophores have been reported to possess excellent antitubercular and anticancer activities. In view of this, we designed, synthesized and characterized a novel series of thiazole–chalcone hybrids (**1**–**20**) and further evaluated them for antitubercular and antiproliferative activities by employing standard protocols. Among the twenty compounds, chalcones **12** and **7**, containing 2,4-difluorophenyl and 2,4-dichlorophenyl groups, showed potential antitubercular activity higher than the standard pyrazinamide (MIC = 25.34 µM) with MICs of 2.43 and 4.41 µM, respectively. Chalcone **20** containing heteroaryl 2-thiazolyl moiety exhibited promising antiproliferative activity against the prostate cancer cell line (DU-145), higher than the standard methotrexate (IC_50_ = 11 ± 1 µM) with an IC_50_ value of 6.86 ± 1 µM. Furthermore, cytotoxicity studies of these compounds against normal human liver cell lines (L02) revealed that the target molecules were comparatively less selective against L02. Additional computational studies using AutoDock predicted the key binding interactions responsible for the activity and the SwissADME tool computed the in silico drug likeliness properties. The lead compounds generated through this study, create a way for the optimization and development of novel drugs against tuberculosis infections and prostate cancer.

## 1. Introduction

**Figure 1:**
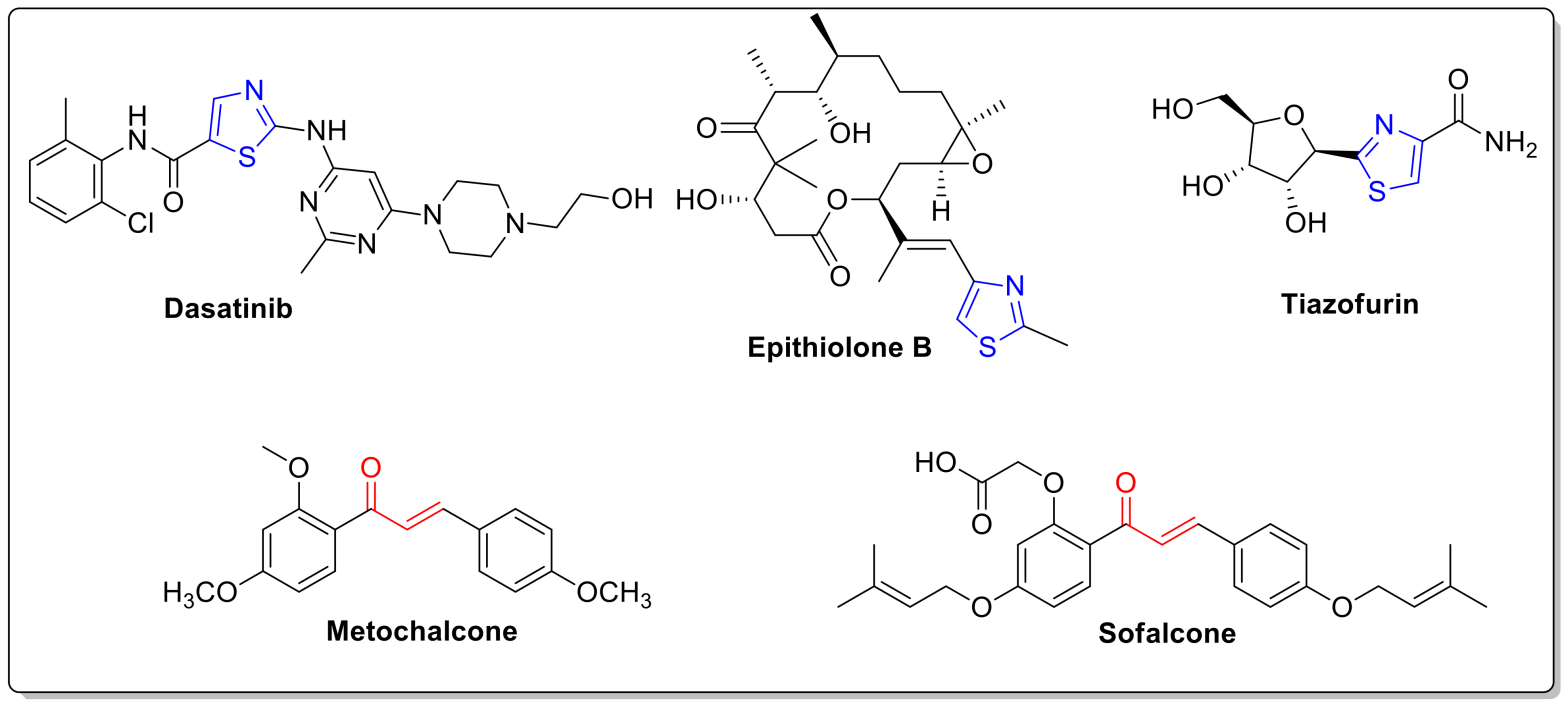
Structures of clinically approved thiazole and chalcone based drugs.

Heterocyclic chemistry plays a pivotal role in the design and development of novel drug molecules as most drugs possess heterocyclic rings. In 2020, the US-FDA approved 24 small molecules and among them 22 molecules contained heterocyclic moieties [1]. This represents the impact of heterocyclic chemistry on the development of new therapies. Thiazole is one such five membered heterocyclic ring which constitutes an essential structural moiety of many marketed drugs. Many anticancer drugs like Dasatinib, Bleomycin, Epothiolone B, Tiazofuran possess a thiazole ring (Figure 1). Additionally, other drugs with a thiazole ring include Abafungin, Ravuconazole (antifungal agents), Acinitrazole, Sulfathiazole (antibacterial agents), Micrococcin, Penicillins, third, fourth and fifth generation cephalosporins (antibacterial antibiotics), Nitazoxanide (antiprotozoal), Ritonavir (antiretroviral agent), Meloxicam (anti-inflammatory), Febuxostat (antigout agent), Famotidine, Nizatidine (antiulcer agents), Pramipexole (antidepressant), Chlormethiazole/Clomethiazole (sedative) and Thiamine (vitamin). Thiazole derivatives were extensively studied by organic and medicinal chemists for their diverse biological activities. The thiazole motif is important for altering the pharmacokinetic and pharmacodynamic properties of the drug molecules. It has exerted different roles in the lead identification and optimization including its use as a pharmacophore or spacer or a bioisosteric scaffold [2,3].

Chalcones are a group of natural open chain flavonoids with promising biological activities [4]. Chemically, chalcones are diarylpropenones (diaryl vinyl ketones) and their structure can be seen in clinically approved drugs including Metochalcone and Sofalcone (Figure 1). The vinyl ketone part of the chalcones is not only responsible for the biological activities of chalcones but is also useful as a key synthon for the preparation of different heterocyclic compounds of pharmacological interest [5]. Thiazole and chalcone derivatives possess a broad spectrum of bioactivities, including antitubercular [6,7,8,9,10,11,12,13,14,15,16,17], anticancer [18,19,20,21,22,23,24,25,26,27,28,29,30,31,32], antioxidant [33,34,35,36,37,38], antifungal [39,40,41,42,43,44,45,46] and antibacterial [47,48,49,50,51,52,53,54,55]. The nature of the substituents as well as the type of the aryl rings connected to the propenone bridge of chalcones governs the intensity of a given biological activity. The improved biological activity of the chalcones was observed with halogenated chalcones and the chalcones in which the aryl ring was replaced with heteroaryl rings. Interestingly, the incorporation of heterocyclic scaffolds and halogen substituted aryl rings improves the ADMET and pharmacodynamic properties of drugs and drug-like candidates [56]. The linking of two biologically active pharmacophoric groups is a practice followed by medicinal chemists for many years through molecular hybridization. Such combination approach will produce molecules with synergistic and higher bioactivity [57]. Additionally, the new structures that emerge due to molecular hybridization are typically rigid pharmacophores that are able to bind effectively to the target.

**Figure 2:**
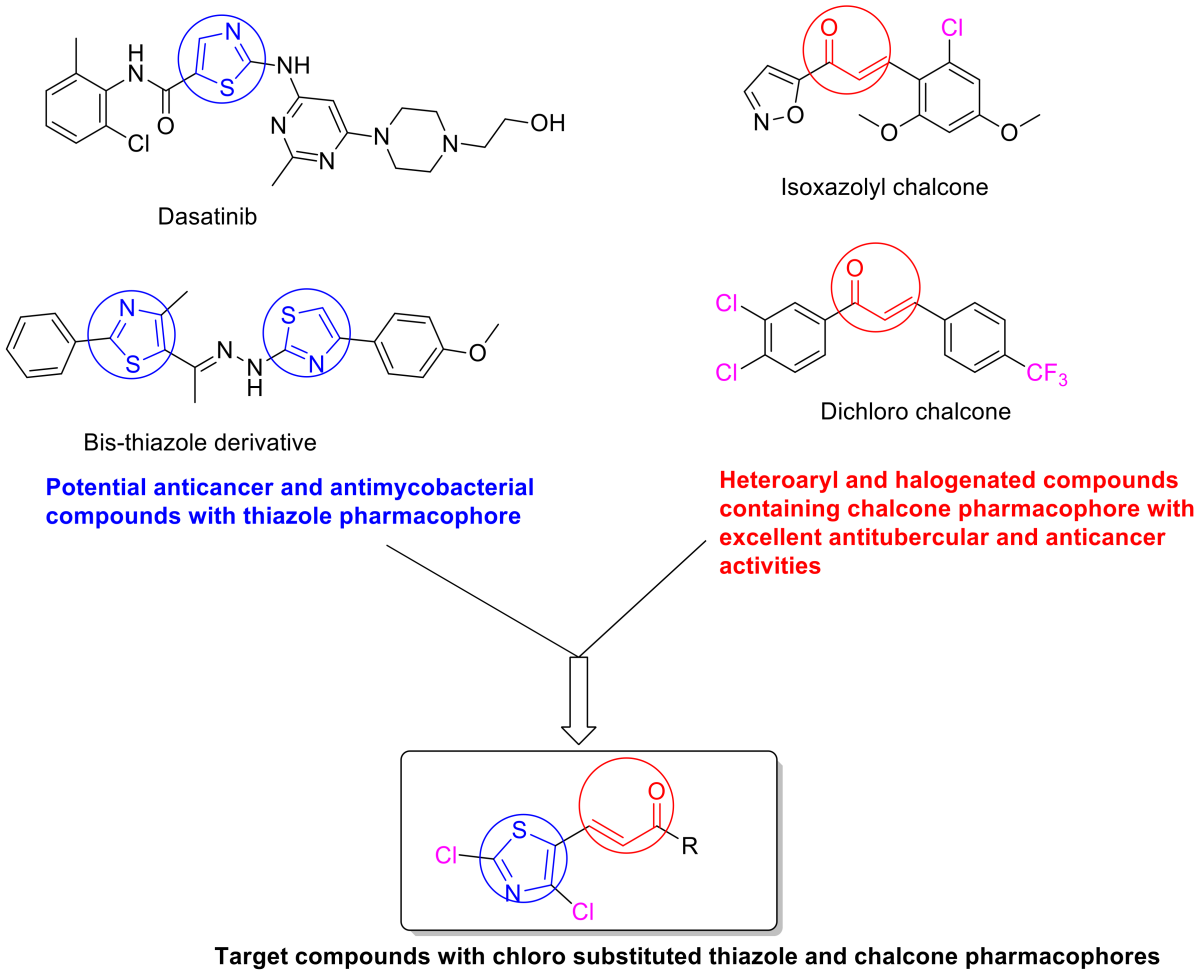
Design of thiazole–chalcone hybrids.

Thiazole–chalcone hybrids have been previously reported with promising antimicrobial [58], anticancer [24] and lipoxygenase (5-LOX) inhibitory [59] activities. In view of the above facts, we designed, synthesized and evaluated the antitubercular, antiproliferative and cytotoxic activities of 20 novel thiazole–chalcone hybrids (**1**–**20**) containing biologically active thiazole and chalcone pharmacophores in order to find novel lead molecules with improved biological activities (Figure 2).

## 2. Results and Discussion

### 2.1. Chemistry

The target thiazole–chalcone hybrids were synthesized by the condensing of different substituted aromatic ketones with 2,4-dichlorothiazole-5-carboxaldehyde in the presence of a glacial acetic acid and hydrochloric acid mixture to isolate the compounds (**1**–**20**) in 75–91% yields. The synthesized hybrids were pale yellow-colored compounds with solubility in chloroform, methanol and DMSO. All the compounds were characterized by FT-IR, ^1^H-NMR and mass spectrometry and the most potent compounds **7**, **12** and **20** were also characterized by ^13^C-NMR spectroscopy. In the FT-IR spectrum, two diagnostic stretching absorption bands of C=O and HC=CH were seen around the wave numbers 1651–1698 cm^−1^ and 1506–1520 cm^−1^, respectively. The ^1^H-NMR spectra showed two doublet signals characteristic of the α- and β-protons of the propenone linkage resonating between the chemical shift values 7.27–7.89 ppm and 7.66–8.16 ppm. The coupling constant value J, for these doublets ranged between 15–17 Hz. These large coupling constant values confirmed the *trans* isomer of the olefinic bond present in the hybrids. ^13^C-NMR spectrum of the compounds displayed the signals at *δ* 181.3–196.1 (C-1, C=O), 122.4–130.5 (C-2, O=C-CH=CH-), and 133.5–146.4 (C-3, O=C-CH=CH-) corresponding to the vinyl ketone portion of chalcone. The molecular ion peak in the mass spectrum further confirmed the formation of chalcones. Additionally, all the compounds showed an isotopic M + 2 peak of one-third intensity to the molecular ion peak. The FT-IR, ^1^H NMR, ^13^C NMR and Mass Spectra for compounds can be found in the Supplementary Materials.

### 2.2. Biological Studies

All the compounds were evaluated for their antitubercular, antiproliferative and cytotoxicity activities by MABA and MTT assays and the results are portrayed in Table 1 and Table 2, respectively. The target compounds (**1**–**20**) were classified into three series (Table 1): monosubstituted phenyl based chalcones (**1**–**3** and **9**–**11**), disubstituted phenyl based chalcones (**4**–**7** and **12**–**16**) and unsubstituted heteroaryl derivatives (bioisosteres of phenyl ring **17**–**20**). The electro withdrawing groups Cl or F were located at ortho (**1**, **9**), meta (**2**, **10**) and para (**3**, **11**) position whereas, in the disubstituted series, Cl or F were substituted at positions 2, 3; 2, 6; 2, 5; 2, 4; 3, 4 or 3, 5 on the phenyl ring.

#### 2.2.1. Antitubercular Activity

The minimum inhibitory concentration (MIC) values for antitubercular activity ranged from 2.43 ± 2 to 701.40 ± 1 µM. Among the twenty compounds, five compounds **7**, **9**, **11**, **12** and **14** displayed more activity than the standard, pyrazinamide (MIC = 25.34 µM). In the monosubstituted series, the ortho (**1**, **9**) and para (**3**, **9**) positions were found to be beneficial for activity over the meta (**2**, **10**) position Among the monosubstituted chalcones **1**–**3**, **9**–**11**, the *ortho* and *para*-F substituted compounds showed 3.79-fold better activity over the Cl-containing compounds (MIC 20.68 ± 2 µM (**9**, **11**) vs. 78.46 ± 1 µM (**1**, **3**)). The MIC value was further improved when the phenyl ring was disubstituted with F over Cl (MIC = 2.43 ± 1 µM (**12**), 4.41 ± 2 µM (**7**)). Compounds **12** and **7** bearing halogen atoms in both ortho and para positions, i.e., 2,4-difluorophenyl (MIC = 2.43 ± 1 µM) and 2,4-dichlorophenyl (MIC = 4.41 ± 2 µM) showed potencies 10.42 and 5.74 times more than the standard (MIC 25.34 ± 2 µM) whereas the monofluorinated compounds **9** and **11** bearing fluorine atoms at ortho and para positions, showed activity at MIC 20.68 µM which was 0.81 times greater than pyrazinamide (25.34 ± 2 µM). The chalcone **14** (MIC = 9.74 ± 2 µM) containing 2,6-difluorophenyl scaffold was 2.6-times more active than the standard. This suggests that the degree of electronegativity played a key role in modulating the physicochemical properties of the phenyl ring thereby influencing the antitubercular activity. Among the bioisosteres **17**–**20**, the activity ranged from 343.45 ± 1 to 701.40 ± 1 µM. No improvement in activity was observed over the standard pyrazinamide.

#### 2.2.2. Antiproliferative and Cytotoxic Activities

With respect to antiproliferative activity, the IC_50′s_ ranged from 6.86 ± 1 to 2900 ± 2 µM. Methotrexate was used as standard, having IC_50_ of 11 ± 1 µM. The disubstituted phenyl based chalcones **5** and **14** fared better over the monosubstituted series (IC_50s_ 90.64 ± 1 µM (**5**), 24.98 ± 2 µM (**14**) vs. 100.43 ± 2 µM to 1607 ± 2 µM (**1**–**3**) and 423.64 ± 2 µM (**10**) to 847.28 ± 2 µM (**11**)). Surprisingly, ortho F phenyl substituted chalcone **9** showed IC_50_ of 52.95 ± 2 µM and this activity was further enhanced two-fold by having F on 2 and 6 position of the phenyl ring (IC_50_ 24.98 ± 2 µM **14**). Replacing the phenyl ring with bioisosteres proved to be beneficial for antiproliferative activity with IC_50s_ ranging from 6.86 ± 1 to 28.05 ± 1 µM (**17**–**20**). The five membered heterocycle 2-thiazolyl based chalcone (**20**) showed the most potent activity with IC_50_ of 6.86 ± 1 µM and was 1.6-fold better than methotrexate. Compounds **17** and **19** containing 2-pyridinyl and 4-pyridinyl scaffolds exhibited IC_50s_ closer to standard. Overall, it can be seen that halogen substituted chalcones showed potential antitubercular activity whereas compounds having unsubstituted heteroaryl scaffold exhibited greater antiproliferative activity (Table 2). Additionally, the cytotoxic activity of the target compounds was found to have IC_50_ above 70 µg/mL on human normal cell lines (L02). It indicated that all the compounds were less selective against L02 than DU-145 and *Mycobacterium tuberculosis* H37Rv strain.

**Figure 3:**
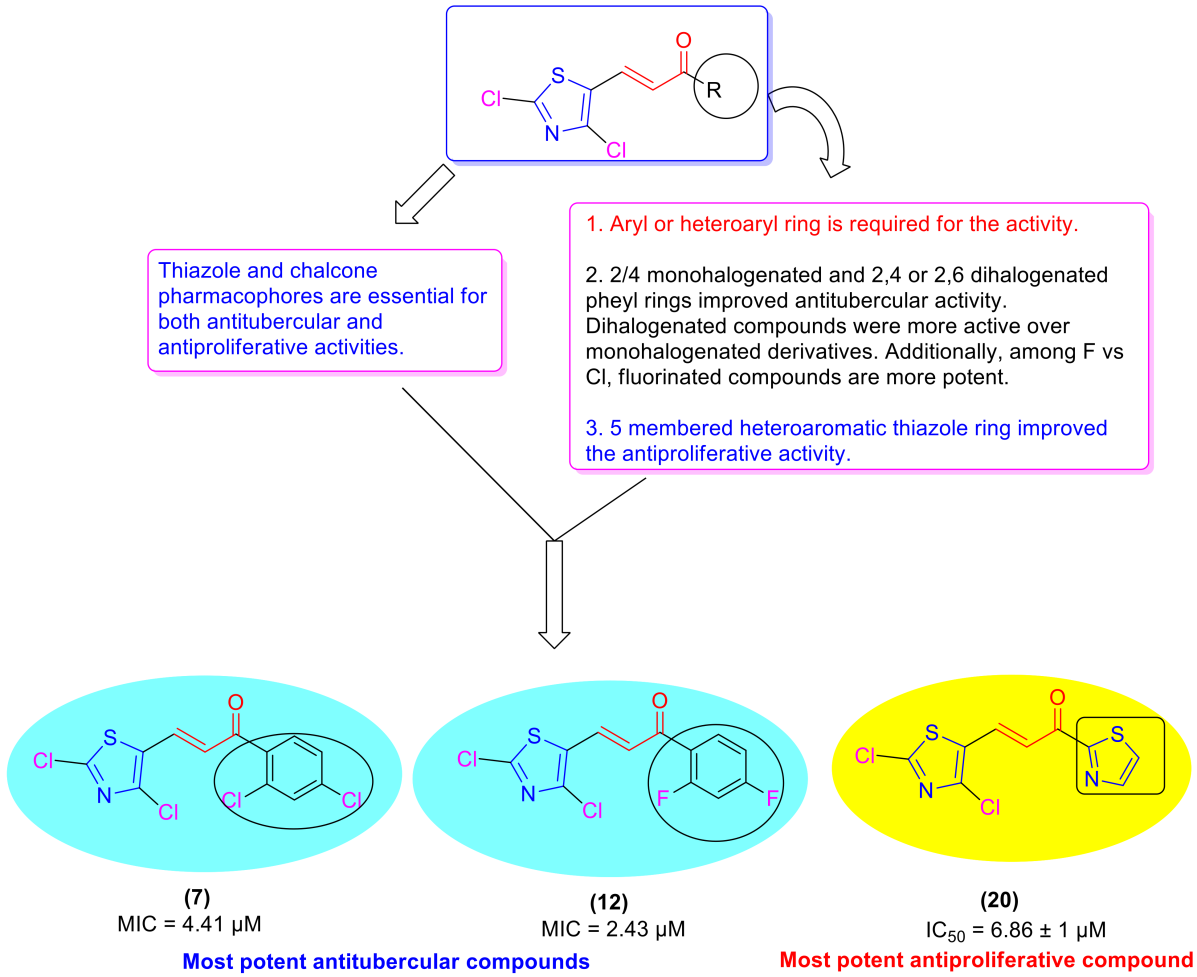
Structure–activity relationship (SAR) of thiazole–chalcone hybrids and structures of most potent antitubercular and antiproliferative compounds.

A summary of the structure–activity relationships (SAR) of novel thiazole–chalcone hybrids and the most potent antitubercular and antiproliferative compounds retrieved from this study are depicted in Figure 3.

### 2.3. Computational Studies

#### 2.3.1. Molecular Docking Studies

Molecular docking was carried out for selected compounds against the potential antitubercular and anticancer targets—Isocitrate Lyase and Topoisomerase IIa ATPase, respectively. The in silico antitubercular and anticancer activity results of selected ligands against Isocitrate Lyase and Topoisomerase IIa ATPase were reported in terms of binding energy and ligand interactions with amino acid residues at active pocket of proteins. The in silico antitubercular results indicated that all compounds (**1**–**20**) showed strong binding affinity (ranges from −5.7 to −7.3, given in Table 3) towards the amino acid residues in active pocket Isocitrate Lyase protein through H-bond and hydrophobic interactions, compared to standard drug Pyrazinamide. The selected compounds having the electron withdrawing group substitution on the aromatic ring in place of the amine group may be the reason for the high affinity of these compounds compared to the standard drug pyrazinamide. Compounds **2** and **16** showed the highest binding affinity (−7.3).

**Figure 4:**
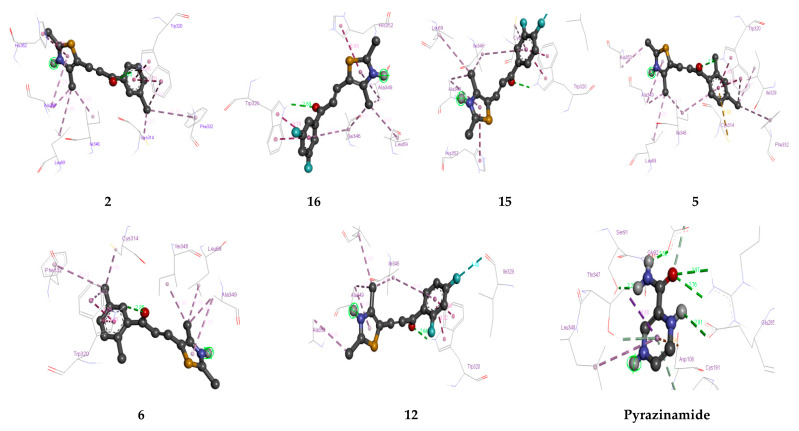
Three-dimensional interactions of selected compounds with Isocitrate Lyase protein amino acid residues.

**Figure 5:**
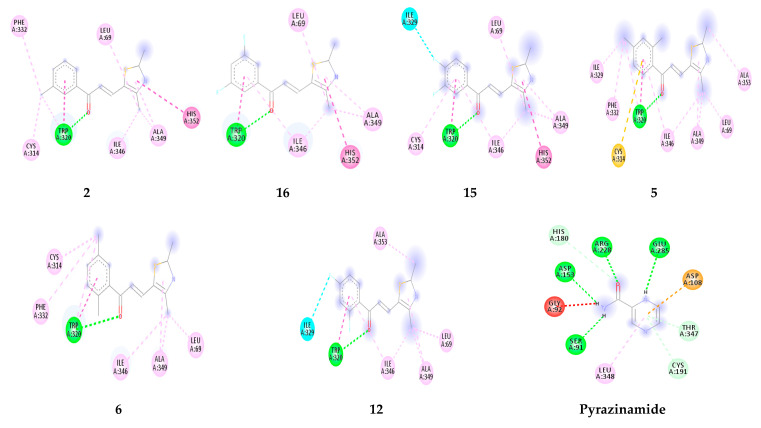
Two-dimensional interactions of selected compounds with Isocitrate Lyase protein amino acid residues.

Among all the compounds, compound **2** interacted with Trp320 amino acid residue through the H-bond and with Leu69, Cys314, Phe332, Ile346, Ala349, His352 amino acid residues through hydrophobic interaction. Similarly compounds **16**, **15**, **5**, **6**, **12** and other compounds also showed H-bond interactions with Trp320 and hydrophobic interaction but the standard drug pyrazinamide has H-bond interaction with Asp153, Arg228, Glu285, Asp108, Ser91, Leu348 amino acid residues and has hydrophobic interaction with Cys191, Thr347, His180 amino acid residues (Figure 4 and Figure 5; Table 4). Moreover, the hydrophobic interactions between the functional groups of compounds and amino acid residues are more, compared to the hydrophobic interactions between the functional groups of standard drug pyrazinamide functional groups and amino acid residues of target protein. The compounds having the electron withdrawing group substituted on the aromatic ring in place of the amine group, and compounds having H-bond interaction with Trp320 amino acid residues and a greater number of hydrophobic interactions present between compounds and amino acid residues in the active site of the target protein may be the possible reason for the high affinity of the compounds when compared to standard drug pyrazinamide.

**Figure 6:**
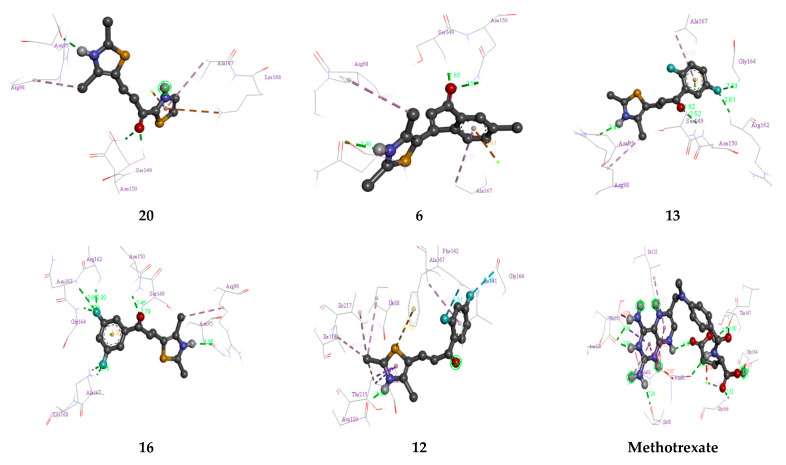
Three-dimensional interactions of selected compounds with Topoisomerase IIa ATPase protein amino acid residues.

**Figure 7:**
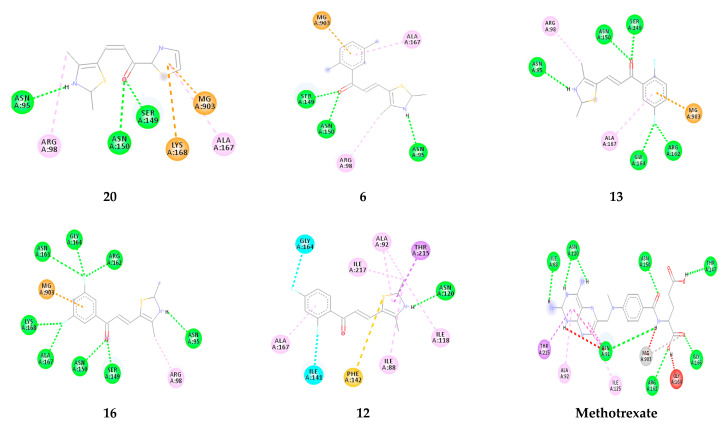
Two-dimensional interactions of selected compounds with Topoisomerase IIa ATPase protein amino acid residues.

The in silico anticancer results of compounds (**1**–**20**) against Topoisomerase IIa ATPase revealed that none of the compounds showed a good binding affinity (ranging from −8.2 to −9.3 as given in Table 5) towards the amino acid residues when compared to the binding affinity of the standard drug Methotrexate (−9.5). The standard drug Methotrexate possessed H-bond interaction with Ile88, Asn120, Thr147, Asn150, Arg162, Gly164, Gly166 amino acid residues and hydrophobic interaction with Asn91, Ala92, Ile125, and Thr215 amino acid residues, given in Table 5 and depicted in Figure 6 and Figure 7. However, compound **20**, having a 2-thiazole substitution, showed the least binding affinity (−9.3) among the all compounds (**1**–**20**), having H-bond interaction with Asn95, Ser149, Asn150, Arg168 and hydrophobic interaction with Arg98, Ala167 amino acid residues. Moreover, compounds with 2,5 dichlorophenyl substitution (**6**), 2, 5-difluoro phenyl substitution (**13**), 3,5-difluoro phenyl substitution (**16**), 2,4-difluoro phenyl substitution (**12**) had moderate binding affinity as -8.4, -8.3 and -8.2, respectively, towards amino acid residues like Asn95, Ser149, asn150, Arg162, Asn163, Gly164, Ala167, Ays168, through the H-bond and amino acid residues like Arg98, Ala167, through hydrophobic interaction. Compounds with electron withdrawing groups at ortho and meta positions of phenyl substituent displayed moderate binding affinities, in addition to the H-bond between the compounds and Asn150 amino acid residue in the active site of target protein which may be highly essential for the good binding affinity of these compounds. The results also indicated that the compounds with 2-thiazoyl substitution had a similar binding affinity to that of the methotrexate.

#### 2.3.2. In Silico Drug Likeliness Studies

Some selected compounds **7**, **14** and **20** which showed the best activity in antitubercular and antiproliferative activity were computed for certain properties using web-based SwissADME software (Table 6). It can be observed that they inhibited CYP2C19, but did not inhibit CYP2D6. However, they showed high GI absorption and passed the Lipinski Rule of five. Hence, these molecules have good drug-like properties and they can be taken as leads for further in vivo investigation.

## 3. Materials and Methods

### 3.1. Chemistry

**Scheme 1:**
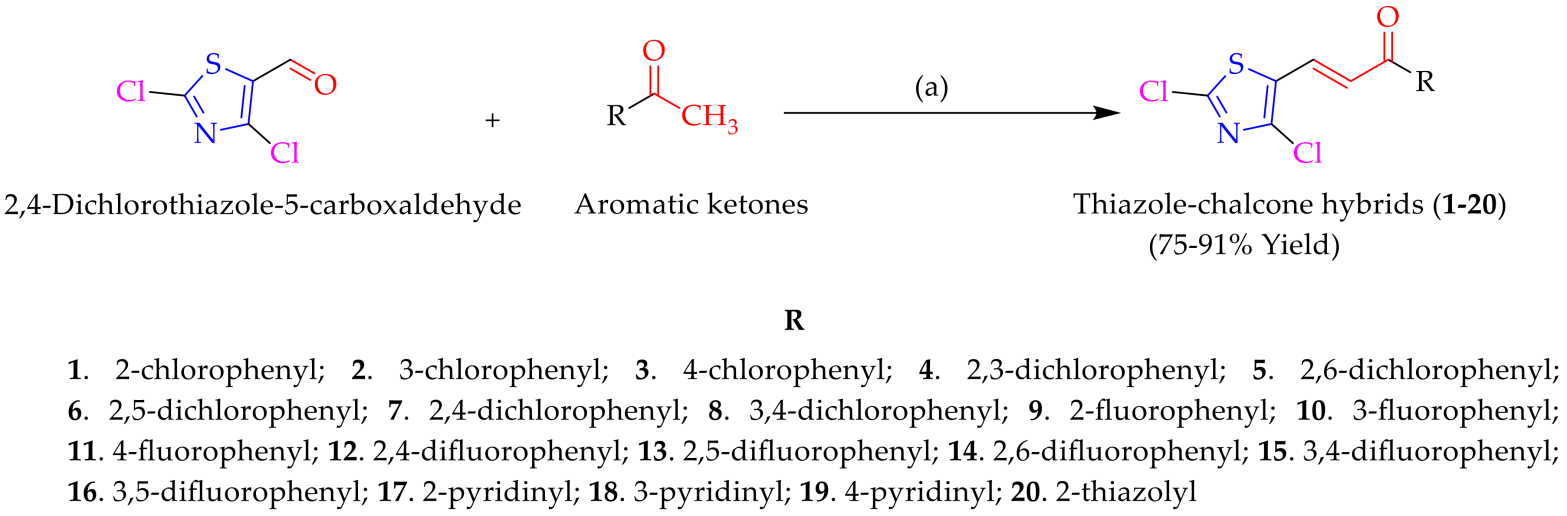
Synthetic scheme for the preparation of novel thiazole–chalcone hybrids: (a) mixture of glacial acetic acid and hydrochloric acid, ethanol, refluxed for 4–6 h.

General protocol for the synthesis of thiazole–chalcone hybrids (**1**–**20**): Initially, 1 mmol of 2,4-dichlorothiazole-5-carboxaldehyde was dissolved in a mixture of glacial acetic acid (4 mL) and concentrated hydrochloric acid (2 mL) [60]. To the above solution, 1 mmol of corresponding aromatic ketone dissolved in 10 mL of ethanol was transferred and refluxed for 4–6 h. After completion of the reaction, the precipitate of the target compound was separated by filtration and washed thoroughly with cold water (50 mL × 2) and dried in a desiccator (Scheme 1). The crude precipitate was purified by column chromatography on silica gel using a mixture of hexane and ethyl acetate (2–25%).

(*E*)-1-(2-chlorophenyl)-3-(2,4-dichlorothiazol-5-yl)prop-2-en-1-one (**1**): Yield: 75%, m.p. 80 °C; FT-IR (KBr, cm^−1^): 1656 (intense conjugated C=O band), 1506 (str, CH=CH, conjugated), 850.91, (C–Cl); ^1^H-NMR spectrum (400 MHz, CDCl_3_, ppm) *δ* (ppm): 7.28 (d, 1H, *J* = 16 Hz), 7.32–7.77 (m, 4H, Ar-H), 7.69 (d, 1H, *J* = 16 Hz); MS (*m*/*z*, %): 318.60 (M^+^, 99.91) 320.60 (M + 2, 33.30).

(*E*)-1-(3-chlorophenyl)-3-(2,4-dichlorothiazol-5-yl)prop-2-en-1-one (**2**): Yield: 79%, m.p. 88 °C; FT-IR (KBr, cm^−1^): 1651 (intense conjugated C=O band), 1508 (str, CH=CH, conjugated); ^1^H-NMR spectrum (400 MHz, CDCl_3_, ppm) *δ* (ppm): 7.27 (d, 1H, *J* = 16 Hz), 7.31–7.81 (m, 4H, Ar-H), 8.01 (d, 1H, *J* = 16 Hz); MS (*m*/*z*, %): 318.60 (M^+^, 99.91) 320.60 (M + 2, 33.30).

(*E*)-1-(4-chlorophenyl)-3-(2,4-dichlorothiazol-5-yl)prop-2-en-1-one (**3**): Yield: 84%, m.p. 104 °C; FT-IR (KBr, cm^−1^): 1656 (intense conjugated C=O band), 1519 (str, CH=CH, conjugated); ^1^H-NMR spectrum (400 MHz, CDCl_3_, ppm) *δ* (ppm): 7.32 (d, 1H, *J* = 16 Hz), 7.36–7.85 (m, 4H, Ar-H), 8.06 (d, 1H, *J* = 16 Hz); MS (*m*/*z*, %): 318.60 (M^+^, 99.91) 320.60 (M + 2, 33.30).

(*E*)-1-(2,3-dichlorophenyl)-3-(2,4-dichlorothiazol-5-yl)prop-2-en-1-one (**4**): Yield: 76%, m.p. 89 °C; FT-IR (KBr, cm^−1^): 1682 (intense conjugated C=O band), 1514 (str, CH=CH, conjugated); ^1^H-NMR spectrum (400 MHz, CDCl_3_, ppm) *δ* (ppm): 7.36 (d, 1H, *J* = 15 Hz), 7.41–7.59 (m, 4H, Ar-H), 7.96 (d, 1H, *J* = 16 Hz); MS (*m*/*z*, %): 353.04 (M^+^, 99.91) 355.04 (M + 2, 33.30).

(*E*)-1-(2,4-dichlorophenyl)-3-(2,4-dichlorothiazol-5-yl)prop-2-en-1-one (**5**): Yield: 88%, m.p. 101 °C; FT-IR (KBr, cm^−1^): 1698 (intense conjugated C=O band), 1515 (str, CH=CH, conjugated); ^1^H-NMR spectrum (400 MHz, CDCl_3_, ppm) *δ* (ppm): 7.38 (d, 1H, *J* = 16 Hz), 7.48–7.67 (m, 4H, Ar-H), 8.16 (d, 1H, *J* = 16 Hz); MS (*m*/*z*, %): 353.04 (M^+^, 99.91) 355.04 (M + 2, 33.30).

(*E*)-1-(2,5-dichlorophenyl)-3-(2,4-dichlorothiazol-5-yl)prop-2-en-1-one (**6**): Yield: 79%, m.p. 84 °C; FT-IR (KBr, cm^−1^): 1677 (intense conjugated C=O band), 1509 (str, CH=CH, conjugated); ^1^H-NMR spectrum (400 MHz, CDCl_3_, ppm) *δ* (ppm): 7.41 (d, 1H, *J* = 16 Hz), 7.56–7.85 (m, 4H, Ar-H), 8.12 (d, 1H, *J* = 16 Hz); MS (*m*/*z*, %): 353.04 (M^+^, 99.91) 355.04 (M + 2, 33.30). 

(*E*)-1-(2,6-dichlorophenyl)-3-(2,4-dichlorothiazol-5-yl)prop-2-en-1-one (**7**): Yield: 83%, m.p. 112 °C; FT-IR (KBr, cm^−1^): 1692 (intense conjugated C=O band), 1520 (str, CH=CH, conjugated); ^1^H-NMR spectrum (400 MHz, CDCl_3_, ppm) *δ* (ppm): 7.89 (d, 1H, *J* = 16 Hz), 7.54–7.75 (m, 4H, Ar-H), 8.09 (d, 1H, *J* = 16 Hz). ^13^C-NMR spectrum (100 MHz, CDCl_3_, ppm): 181.3 (C-1), 124.5 (C-2), 139.6 (C-3), 127.2, 131.1, 132.4, 134.5, 135.1, 135.9, 136.3, 146.3, 152.1 (Ar-C); MS (*m*/*z*, %): 353.04 (M^+^, 99.91) 355.04 (M + 2, 33.30).

(*E*)-1-(3,4-dichlorophenyl)-3-(2,4-dichlorothiazol-5-yl)prop-2-en-1-one (**8**): Yield: 86%, m.p. 106 °C; FT-IR (KBr, cm^−1^): 1675 (intense conjugated C=O band), 1516 (str, CH=CH, conjugated); ^1^H-NMR spectrum (400 MHz, CDCl_3_, ppm) *δ* (ppm): 7.66 (d, 1H, *J* = 16 Hz), 7.19–7.51 (m, 4H, Ar-H), 8.11 (d, 1H, *J* = 16 Hz); MS (*m*/*z*, %): 353.04 (M^+^, 99.91) 355.04 (M + 2, 33.30).

(*E*)-1-(2-fluorophenyl)-3-(2,4-dichlorothiazol-5-yl)prop-2-en-1-one (**9**): Yield: 77%, m.p. 91 °C; FT-IR (KBr, cm^−1^): 1679 (intense conjugated C=O band), 1508 (str, CH=CH, conjugated); ^1^H-NMR spectrum (400 MHz, CDCl_3_, ppm) *δ* (ppm): 7.45 (d, 1H, *J* = 16 Hz), 7.59–7.84 (m, 4H, Ar-H), 8.15 (d, 1H, *J* = 17 Hz); MS (*m*/*z*, %): 302.14 (M^+^, 99.91) 304.14 (M + 2, 33.30).

(*E*)-1-(3-fluorophenyl)-3-(2,4-dichlorothiazol-5-yl)prop-2-en-1-one (**10**): Yield: 76%, m.p. 112 °C; FT-IR (KBr, cm^−1^): 1688 (intense conjugated C=O band), 1516 (str, CH=CH, conjugated); ^1^H-NMR spectrum (400 MHz, CDCl_3_, ppm) *δ* (ppm): 7.33 (d, 1H, *J* = 16 Hz), 7.45–7.84 (m, 4H, Ar-H), 8.14 (d, 1H, *J* = 16 Hz); MS (*m*/*z*, %): 302.14 (M^+^, 99.91) 304.14 (M + 2, 33.30).

(*E*)-1-(4-fluorophenyl)-3-(2,4-dichlorothiazol-5-yl)prop-2-en-1-one (**11**): Yield: 83%, m.p. 126 °C; FT-IR (KBr, cm^−1^): 1681 (intense conjugated C=O band), 1518 (str, CH=CH, conjugated); ^1^H-NMR spectrum (400 MHz, CDCl_3_, ppm) *δ* (ppm): 7.42 (d, 1H, *J* = 16 Hz), 7.55–7.91 (m, 4H, Ar-H), 8.16 (d, 1H, *J* = 16 Hz); MS (*m*/*z*, %): 302.14 (M^+^, 99.91) 304.14 (M + 2, 33.30).

(*E*)-1-(2,4-difluorophenyl)-3-(2,4-dichlorothiazol-5-yl)prop-2-en-1-one (**12**): Yield: 91%, m.p. 134 °C; FT-IR (KBr, cm^−1^): 1656.38 (intense conjugated C=O band), 1497.38 (str, CH=CH, conjugated); ^1^H-NMR spectrum (400 MHz, CDCl_3_, ppm) *δ* (ppm): 7.51 (d, 1H, *J* = 16 Hz), 7.77–7.96 (m, 4H, Ar-H), 8.16 (d, 1H, *J* = 16 Hz). ^13^C-NMR spectrum (100 MHz, CDCl_3_, ppm): 196.1 (C-1), 130.5 (C-2), 146.4 (C-3), 128.6, 132.3, 133.8, 135.7, 136.4, 137.3, 138.5, 148.6, 154.6 (Ar-C); MS (*m*/*z*, %): 320.13 (M^+^, 99.91) 322.13 (M + 2, 33.30).

(*E*)-1-(2,5-difluorophenyl)-3-(2,4-dichlorothiazol-5-yl)prop-2-en-1-one (**13**): Yield: 77%, m.p. 129 °C; FT-IR (KBr, cm^−1^): 1674 (intense conjugated C=O band), 1512 (str, CH=CH, conjugated); ^1^H-NMR spectrum (400 MHz, CDCl_3_, ppm) *δ* (ppm): 7.55 (d, 1H, *J* = 16 Hz), 7.81–7.98 (m, 4H, Ar-H), 8.15 (d, 1H, *J* = 16 Hz); MS (*m*/*z*, %): 320.13 (M^+^, 99.91) 322.13 (M + 2, 33.30).

(*E*)-1-(2,6-difluorophenyl)-3-(2,4-dichlorothiazol-5-yl)prop-2-en-1-one (**14**): Yield: 80%, m.p. 141 °C; FT-IR (KBr, cm^−1^): 1666 (intense conjugated C=O band), 1511 (str, CH=CH, conjugated); ^1^H-NMR spectrum (400 MHz, CDCl_3_, ppm) *δ* (ppm): 7.69 (d, 1H, *J* = 16 Hz), 7.85–7.99 (m, 4H, Ar-H), 8.18 (d, 1H, *J* = 16 Hz); MS (*m*/*z*, %): 320.13 (M^+^, 99.91) 322.13 (M + 2, 33.30).

(*E*)-1-(3,4-difluorophenyl)-3-(2,4-dichlorothiazol-5-yl)prop-2-en-1-one (**15**): Yield: 88%, m.p. 145 °C; FT-IR (KBr, cm^−1^): 1675 (intense conjugated C=O band), 1516 (str, CH=CH, conjugated); ^1^H-NMR spectrum (400 MHz, CDCl_3_, ppm) *δ* (ppm): 7.33 (d, 1H, *J* = 16 Hz), 7.51–7.91 (m, 4H, Ar-H), 8.16 (d, 1H, *J* = 16 Hz); MS (*m*/*z*, %): 320.13 (M^+^, 99.91) 322.13 (M + 2, 33.30).

(*E*)-1-(3,5-difluorophenyl)-3-(2,4-dichlorothiazol-5-yl)prop-2-en-1-one (**16**): Yield: 78%, m.p. 109 °C; FT-IR (KBr, cm^−1^): 1688 (intense conjugated C=O band), 1519 (str, CH=CH, conjugated); ^1^H-NMR spectrum (400 MHz, CDCl_3_, ppm) *δ* (ppm): 7.36 (d, 1H, *J* = 16 Hz), 7.55–7.89 (m, 4H, Ar-H), 8.05 (d, 1H, *J* = 16 Hz); MS (*m*/*z*, %): 320.13 (M^+^, 99.91) 322.13 (M + 2, 33.30).

(*E*)-3-(2,4-dichlorothiazol-5-yl)-1-(pyridin-2-yl)prop-2-en-1-one (**17**): Yield: 76%, m.p. 156 °C; FT-IR (KBr, cm^−1^): 1658 (intense conjugated C=O band), 1509 (str, CH=CH, conjugated); ^1^H-NMR spectrum (400 MHz, CDCl_3_, ppm) *δ* (ppm): 7.32 (d, 1H, *J* = 16 Hz), 7.42–7.67 (m, 4H, Ar-H), 7.99 (d, 1H, *J* = 16 Hz); MS (*m*/*z*, %): 285.14 (M^+^, 99.91) 287.14 (M + 2, 33.30).

(*E*)-3-(2,4-dichlorothiazol-5-yl)-1-(pyridin-3-yl)prop-2-en-1-one (**18**): Yield: 81%, m.p. 162 °C; FT-IR (KBr, cm^−1^): 1662 (intense conjugated C=O band), 1506 (str, CH=CH, conjugated); ^1^H-NMR spectrum (400 MHz, CDCl_3_, ppm) *δ* (ppm): 7.38 (d, 1H, *J* = 16 Hz), 7.48–7.71 (m, 4H, Ar-H), 7.94 (d, 1H, *J* = 16 Hz); MS (*m*/*z*, %): 285.14 (M^+^, 99.91) 287.14 (M + 2, 33.30).

(*E*)-3-(2,4-dichlorothiazol-5-yl)-1-(pyridin-4-yl)prop-2-en-1-one (**19**): Yield: 78%, m.p. 177 °C; FT-IR (KBr, cm^−1^): 1660 (intense conjugated C=O band), 1509 (str, CH=CH, conjugated); ^1^H-NMR spectrum (400 MHz, CDCl_3_, ppm) *δ* (ppm): 7.34 (d, 1H, *J* = 16 Hz), 7.46–7.75 (m, 4H, Ar-H), 7.98 (d, 1H, *J* = 16 Hz); MS (*m*/*z*, %): 285.14 (M^+^, 99.91) 287.14 (M + 2, 33.30).

(*E*)-3-(2,4-dichlorothiazol-5-yl)-1-(thiazol-2-yl)prop-2-en-1-one (**20**): Yield: 76%, m.p. 127 °C; FT-IR (KBr, cm^−1^): 1657 (intense conjugated C=O band), 1507 (str, CH=CH, conjugated); ^1^H-NMR spectrum (400 MHz, CDCl_3_, ppm) *δ* (ppm): 7.36 (d, 1H, *J* = 16 Hz), 7.41–7.51 (m, 2H, Ar-H), 8.06 (d, 1H, *J* = 16 Hz). ^13^C-NMR spectrum (100 MHz, CDCl_3_, ppm): 190.6 (C-1), 122.4 (C-2), 133.5 (C-3), 127.8, 131.8, 135.9, 137.3, 136.2, 144.5, 151.2 (Ar-C); MS (*m*/*z*, %): 291.16 (M^+^, 99.91) 293.16 (M + 2, 33.30).

### 3.2. Biological Studies

#### 3.2.1. Antitubercular Activity

The antitubercular activity of all the target compounds (**1**–**20**) was tested against the *Mycobacterium tuberculosis H37Rv* strain. Pyrazinamide was used as the reference standard. In the present investigation, we employed the protocol described in the literature [61,62]. The icy culture of *Mycobacterium tuberculosis H37Rv* strain in Middlebrook 7H9 broth with the addition of 0.2% glycerol and 10% albumin-dextrose-catalase was defrosted and diluted in broth to 10^5^ CFU mL^−1^ (colony forming unit/mL) dilutions. Separately, test compounds were dissolved in Dimethyl sulfoxide (DMSO) and later diluted with broth to achieve a concentration that was two-times the required concentration. Throughout this experiment, the final concentration of DMSO was 1.3% in the assay. Each test-tube used was then inoculated with 0.05 mL of standardized culture and then incubated for 21 days at 37 °C. The growth in the test-tubes was compared with the positive control, pyrazinamide and negative control, i.e., without inoculum and the drug. The minimum inhibitory concentration (MIC) of the individual target compound was determined by broth dilution assay. The MIC values were obtained in µg/mL and further these values were converted to micromoles (µM) considering the structural diversity of standard drug and the target compounds to draw a more meaningful conclusion.

#### 3.2.2. Antiproliferative and Cytotoxic Activity

The in vitro antiproliferative and cytotoxicity of compounds **1**–**20** was evaluated by Mosmann’s MTT (3-(4,5-dimethylthiazol-2-yl)-2,5-diphenyl tetrazolium bromide) assay method, as described in the literature [63,64,65] on prostate cancer cell lines (DU-145). In both the assays, the IC_50_ values of the tested compounds were compared with the positive control Methotrexate (Mtx). The reduction of the soluble MTT to blue-color formazan is the principle underlined in MTT assay and such change is chiefly due to the action of intracellular mitochondrial reductase in the living cells. The prostate cancer cell lines (DU-145), were cultured in Dulbecco’s Modified Eagle Medium (DMEM**)** media at 37 °C and humidified at 5% CO_2_ was used to culture prostate cancer cell lines (DU-145). The compounds (**1**–**20**) were initially dissolved in 0.1% DMSO to prepare their stock solutions. Later, the desired concentrations of the compounds were achieved by dissolving in sterile water. The cells were transferred on to 96-well plates at 100 µL total volume and with a density of 1 × 10^4^ cells per well. The cells were permitted to adhere for a period of 24 h and then the assay medium was replaced with a fresh medium containing target compounds and incubated in DMEM with 10% fetal bovine serum (FBS) medium at 37 °C for an additional 48 h. Later, the medium was replaced with 90 μL of fresh DMEM without FBS. The above wells were treated with 10 μL of MTT reagent (5 mg/mL of stock solution in DMEM without FBS) and incubated for 3–4 h at 37 °C. The formed blue formazan crystals were dissolved in 200 μL of DMSO. The optical density was then determined at 570 nm using a micro plate reader. The assay was executed in triplicate for three independent experiments. The same trialing was also performed to confirm that the negative control-DMSO had no effect in the study. The results had good reproducibility between replicate wells with standard errors below 10%. The IC_50_ values measured in µg/mL for the antiproliferative were converted and expressed in µM. Additionally, all the compounds were also assessed for their cytotoxic activity on the normal human liver cell lines (L02) using the same protocol discussed above.

### 3.3. Computational Studies

#### 3.3.1. Molecular Docking Studies

The X-ray crystal structures of Isocitrate Lyase (PDB ID: 1F8M) and Topoisomerase IIa ATPase (PDB ID: 1ZXM) were taken from protein data bank (rcsb.com/pdb database (accessed on 7 April 2021)). Using PyMOL 2.3.4 the water molecules were removed and hydrogens were added as well as co-crystal ligands were extracted and saved in mol2 format. The mol2 format file of protein was loaded and then converted to pdbqt format using Autodock module Macromolecule tool in PyRx Virtual screening software 0.8 [66]. The 2D-structures of the target compounds (**1–20**) and that of the standard drugs-pyrazinamide and methotrexate were drawn in ChemDraw ultra 12.0 and saved as sdf file. The ligand files were subjected to energy minimization (force field-uff) through Open babel tool and then conformers for the selected ligands were generated through AutoDock pdbqt files in PyRx Virtual screening software 0.8. The docking was then performed through PyRx Virtual screening software 0.8 combined with AutoDock Vina, Open babel, Python shell tools. The prepared protein file and ligand files were selected through Vina module and the grid box was selected according to the previously reported amino acid residues by adjusting the x, y, z coordinates of grid box, then run the Vina. The results were analyzed by using DS visualizer software to visualize the interactions between ligands and amino acid residues of active site of protein (Kwofie et al., 2018).

#### 3.3.2. In Silico Drug Likeliness Studies

To meet the requirements of the drug-likeliness, the properties of the most potent compounds **7**, **14** and **20** target compounds were evaluated for their in silico parameters including GI absorption, Lipinski rule of five as well as CYP2C19 CYP2D6 inhibition using SwissADME web (http://www.swissadme.ch/ (accessed on 24 March 2021)) [67].

## 4. Conclusions

In the present study we designed and synthesized 20 new thiazole–chalcone hybrids and tested all the compounds for antitubercular, antiproliferative and cytotoxic activities against *Mycobacterium tuberculosis* H37Rv strain, DU-145 (prostate cancer cell line) and human liver normal cells—LO2, respectively. We identified five potential antitubercular chalcones and one promising antiproliferative chalcone as they displayed better activity than the standard drugs. Among the five potent hybrids, the compounds **12** and **7** exhibited the strongest antitubercular activity with MIC values 2.43 and 4.41 µM correspondingly. The top most active antiproliferative hybrid **20** showed activity at IC_50_ value 6.86 ± 1 µM. All the compounds were subjected to molecular docking studies against antitubercular and anticancer drug targets and a good correlation was observed between the in vitro and docking results. Additionally, the calculated SwissADME properties of the most potent compounds **7**, **14** and **20** were in agreement with the required drug-like properties. All these results clearly confirm the usefulness of the active compounds for the furtherance of drug discovery and development against tuberculosis and prostate cancer.

## Figures and Tables

**Table 1:**
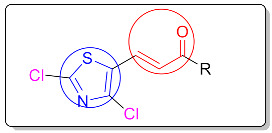
Antitubercular results of thiazole–chalcone hybrids (**1**–**20**).

Entry	R	Mtb (H37Rv Strain) (MIC in µM) ^a^
**1**	2-chlorophenyl	78.46 ± 1
**2**	3-chlorophenyl	313.87 ± 1
**3**	4-chlorophenyl	78.46 ± 1
**4**	2,3-dichlorophenyl	141.62 ± 2
**5**	2,6-dichlorophenyl	35.40 ± 2
**6**	2,5-dichlorophenyl	141.62 ± 1
**7**	2,4-dichlorophenyl	**4.41** ± 2
**8**	3,4-dichlorophenyl	141.62 ± 1
**9**	2-fluorophenyl	**20.68** ± 2
**10**	3-fluorophenyl	165.48 ± 1
**11**	4-fluorophenyl	**20.68** ± 1
**12**	2,4-difluorophenyl	**2.43** ± 1
**13**	2,5-difluorophenyl	39.04 ± 1
**14**	2,6-difluorophenyl	**9.74** ± 2
**15**	3,4-difluorophenyl	39.04 ± 1
**16**	3,5-difluorophenyl	156.18 ± 2
**17**	2-pyridinyl	350.70 ± 2
**18**	3-pyridinyl	701.40 ± 1
**19**	4-pyridinyl	350.70 ± 2
**20**	2-thiazolyl	343.45 ± 1
**Pyrazinamide**		25.34 ± 2

^a^ MICs are mean of three independent experiments. Bold numerical values in third column represent compounds with highest activity.

**Table 2:**
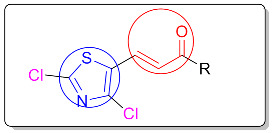
Antiproliferative and cytotoxic activity results of thiazole–chalcone hybrids (**1**–**20**).

Entry	R	Prostate Cancer Cell Line (DU-145) (IC_50_ in µM) ^b^	Normal Liver Cell Line (L02) (IC_50_ in µg/mL) ^b^
**1**	2-chlorophenyl	100.43 ± 2	>70
**2**	3-chlorophenyl	1607.03 ± 2	>70
**3**	4-chlorophenyl	401.75 ± 1	>70
**4**	2,3-dichlorophenyl	181.28 ± 1	>70
**5**	2,6-dichlorophenyl	90.64 ± 1	>70
**6**	2,5-dichlorophenyl	2900.52 ± 2	>70
**7**	2,4-dichlorophenyl	181.28 ± 1	>70
**8**	3,4-dichlorophenyl	1450.26 ± 1	>70
**9**	2-fluorophenyl	52.95 ± 2	>70
**10**	3-fluorophenyl	847.28 ± 2	>70
**11**	4-fluorophenyl	423.64 ± 2	>70
**12**	2,4-difluorophenyl	99.95 ± 1	>70
**13**	2,5-difluorophenyl	3198.70 ± 2	>70
**14**	2,6-difluorophenyl	24.98 ± 2	>70
**15**	3,4-difluorophenyl	799.67 ± 1	>70
**16**	3,5-difluorophenyl	3198.70 ± 1	>70
**17**	2-pyridinyl	14.02 ± 1	>70
**18**	3-pyridinyl	28.05 ± 1	>70
**19**	4-pyridinyl	14.02 ± 1	>70
**20**	2-thiazolyl	**6.86 ± 1**	>70
**Methotrexate**		11 ± 1	>70

^b^ Data presented as mean ± SD (*n* = 3). All the compounds and the standard dissolved in DMSO, diluted with culture medium containing 0.1% DMSO. The control cells were treated with culture medium containing 0.1% DMSO. Bold numerical value in third column represents compound with highest activity.

**Table 3:** Docking scores of thiazole–chalcone hybrids against Isocitrate Lyase protein and Topoisomerase IIa ATPase.

Compound	Binding Affinity		Compound	Binding Affinity	
	1F8M	1ZXM		1F8M	1ZXM
**1**	−6.7	−8.1	**11**	−7	−7.8
**2**	−7.3	−8.1	**12**	−7.1	−8.2
**3**	−7	−7.7	**13**	−7.2	−8.4
**4**	−6.9	−7.9	**14**	−6.8	−8.2
**5**	−7.2	−7.9	**15**	−7.2	−8.2
**6**	−7.2	−8.1	**16**	−7.3	−8.3
**7**	−6.6	−8.1	**17**	−6.3	−7.6
**8**	−6.8	−8.1	**18**	−6.2	−7.7
**9**	−6.9	−8.1	**19**	−6.2	−7.7
**10**	−7.1	−8.1	**20**	−5.7	−9.3
**Methotrexate**	-	−9.5	**Pyrazinamide**	−5.4	-

**Table 4:** Molecular interactions of thiazole–chalcone hybrids with highest docking score against Isocitrate Lyase protein.

Compound	Docking Score	Amino Acids Interacted Through	
		H-Bond	Hydrophobic
**2**	−7.3	Trp320	Leu69, Cys314, Phe332, Ile346, Ala349, His352
**16**	−7.3	Trp320	Leu69, Ile346, Ala349, His352
**15**	−7.2	Trp320	Lue69, Ile329, Cys314, Ile346, Ala349, His352
**5**	−7.2	Trp320	Leu69, Ile329, Phe332, Ile346, Ala349, Ala353
**6**	−7.2	Trp320	Leu69, Lys315, Phe332, Ile346, Ala349
**12**	−7.1	Trp320	Leu69, Ile329, Ile346, Ala349, Ala353
**Pyrazinamide**	−5.4	Asp153, Arg228, Glu285, Asp108, Ser91, Leu348	Cys191, Thr347, His180

**Table 5:** Molecular interactions of thiazole–chalcone hybrids with highest docking score against Topoisomerase IIa ATPase.

Compound	Docking Score	Amino Acids Interacted Through	
		H-Bond	Hydrophobic
**20**	−9.3	Asn95, Ser149, Asn150, Lys168	Arg98, Ala167
**6**	−8.5	Asn95, Ser149, Asn150	Arg98, Ala167
**13**	−8.4	Asn95, Ser149, asn150, Arg162, Gly164	Arg98, Ala167
**16**	−8.3	Asn95, Ser149, Asn150, Arg162, Asn163, Gly163, Ala167, Ays168	Arg98
**12**	−8.2	Asn120, Phe142	Ile88, Ala92, Ile118, Ile141, Gly164, Ala167, Thr215, Ile217
**Methotrexate**	−9.5	Ile88, Asn120, Thr147, Asn150, Arg162, Gly164, Gly166	Asn91, Ala92, Ile125, Thr215

**Table 6:** Computed properties using SWISSADME.

Compound #	GI Absorption	CYP2C19 Inhibitor	CYP2D6 Inhibitor	Lipinski #Violations
**7**	High	Yes	No	0
**14**	High	Yes	No	0
**20**	High	Yes	No	0

## Supplementary Materials

The following are available online: FT-IR, ^1^H NMR, ^13^C NMR and Mass Spectra for compounds.

## Sample Availability

Samples of the compounds are available from the authors.
