# Supplementary material for: Thiazole–Chalcone Hybrids as Prospective Antitubercular and Antiproliferative Agents: Design, Synthesis, Biological, Molecular Docking Studies and In Silico ADME Evaluation
Source: Molecules. 2021 May 11;26(10):2847. doi: 10.3390/molecules26102847 (PMC8151732; doi:10.3390/molecules26102847)
Supplement: Supplementary file 1 [file molecules-26-02847-s001.zip › molecules-1194574-supplementary.pdf]

Thiazole-Chalcone Hybrids as prospective antitubercular and antiproliferative agents: Design, synthesis, biological, molecular docking studies and in-silico ADME evaluation

Ashok Babu Kasetti, Indrajeet Singhvi, Ravindra Nagasuri, Richie R. Bhandare and Afzal B. Shaik

FT-IR, ^{1}H NMR, ^{13}C NMR & Mass Spectra

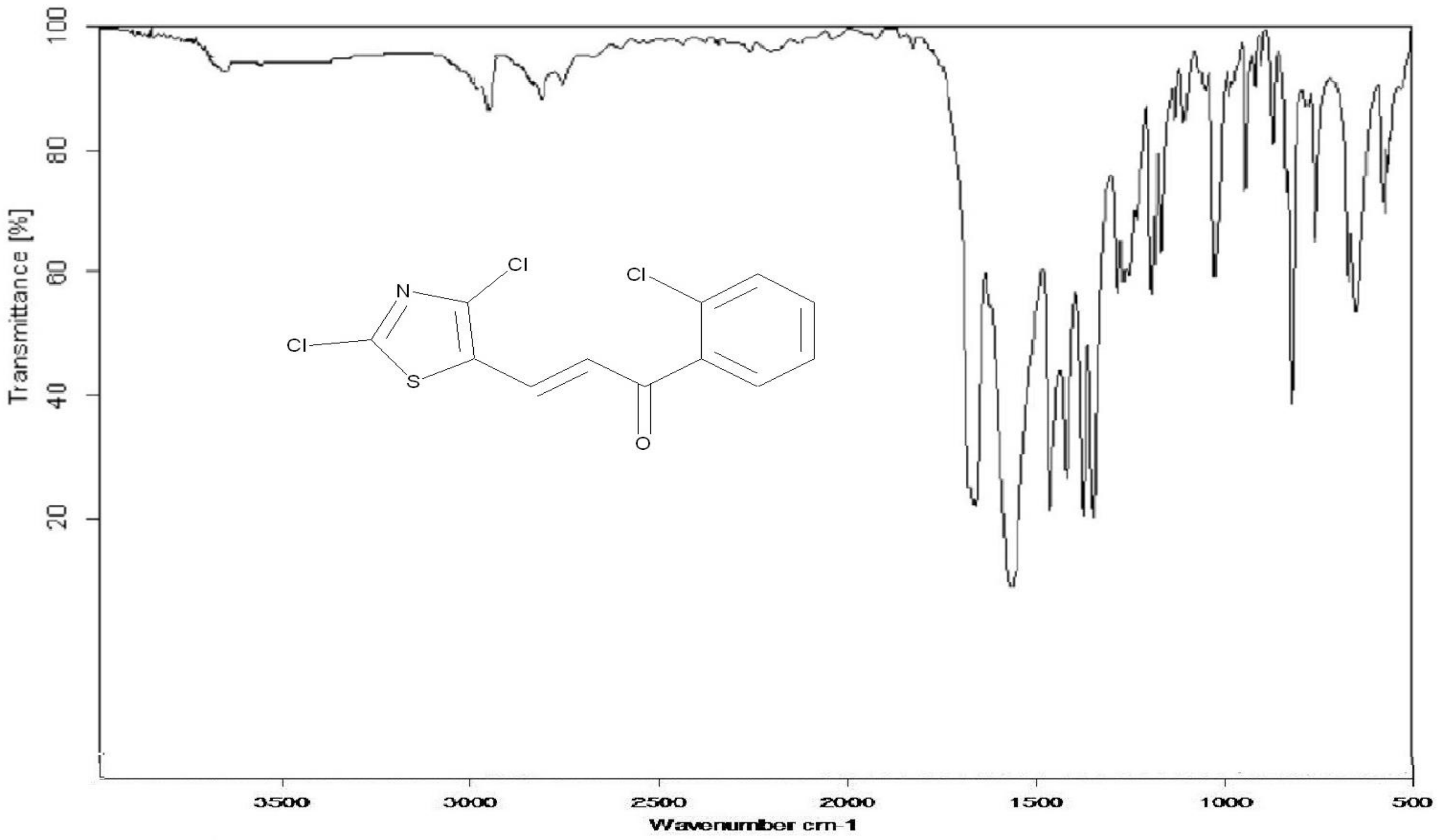

Figure S1. FT-IR Spectrum of compound 1

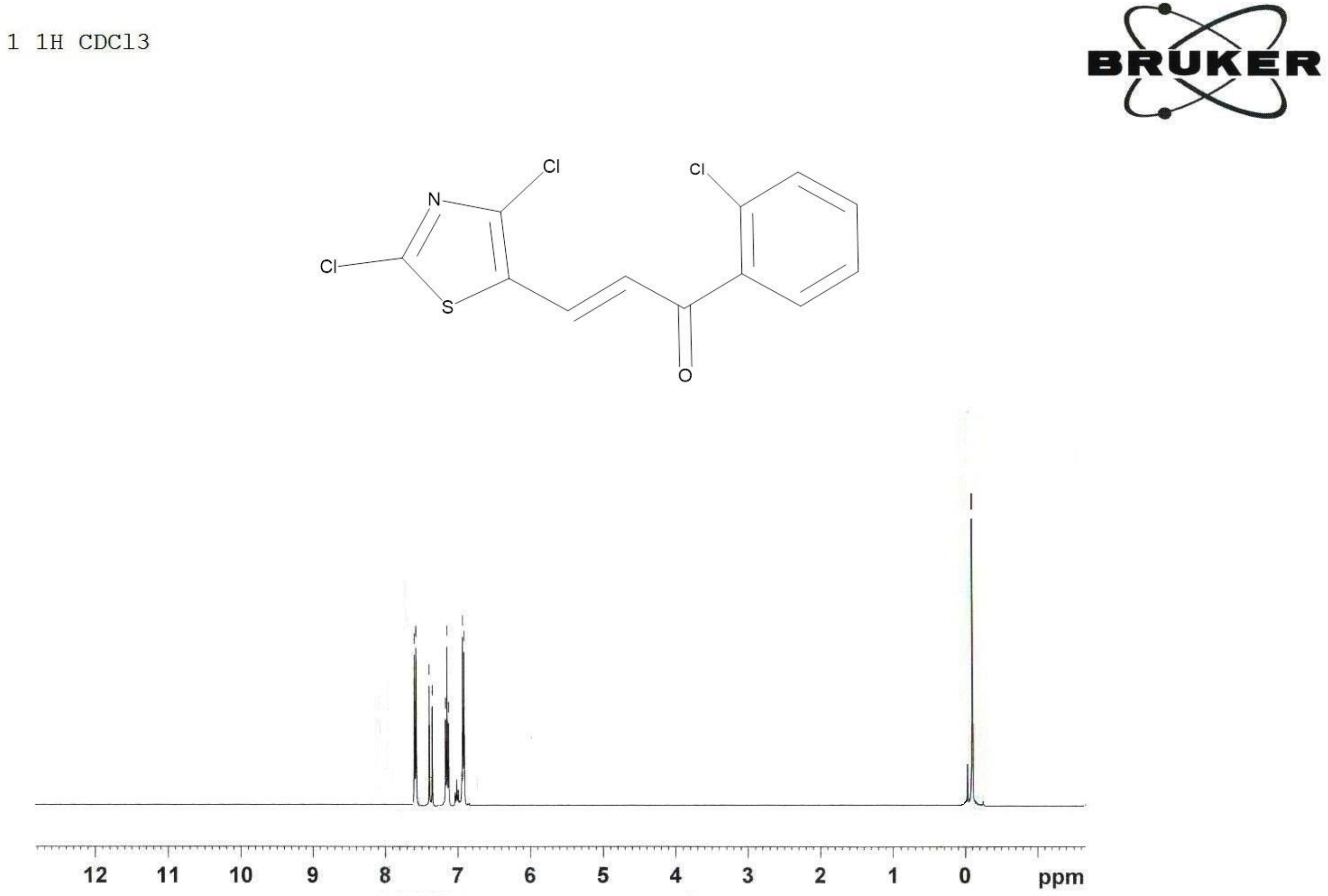

Figure S2. ^{1}H NMR Spectrum of compound 1

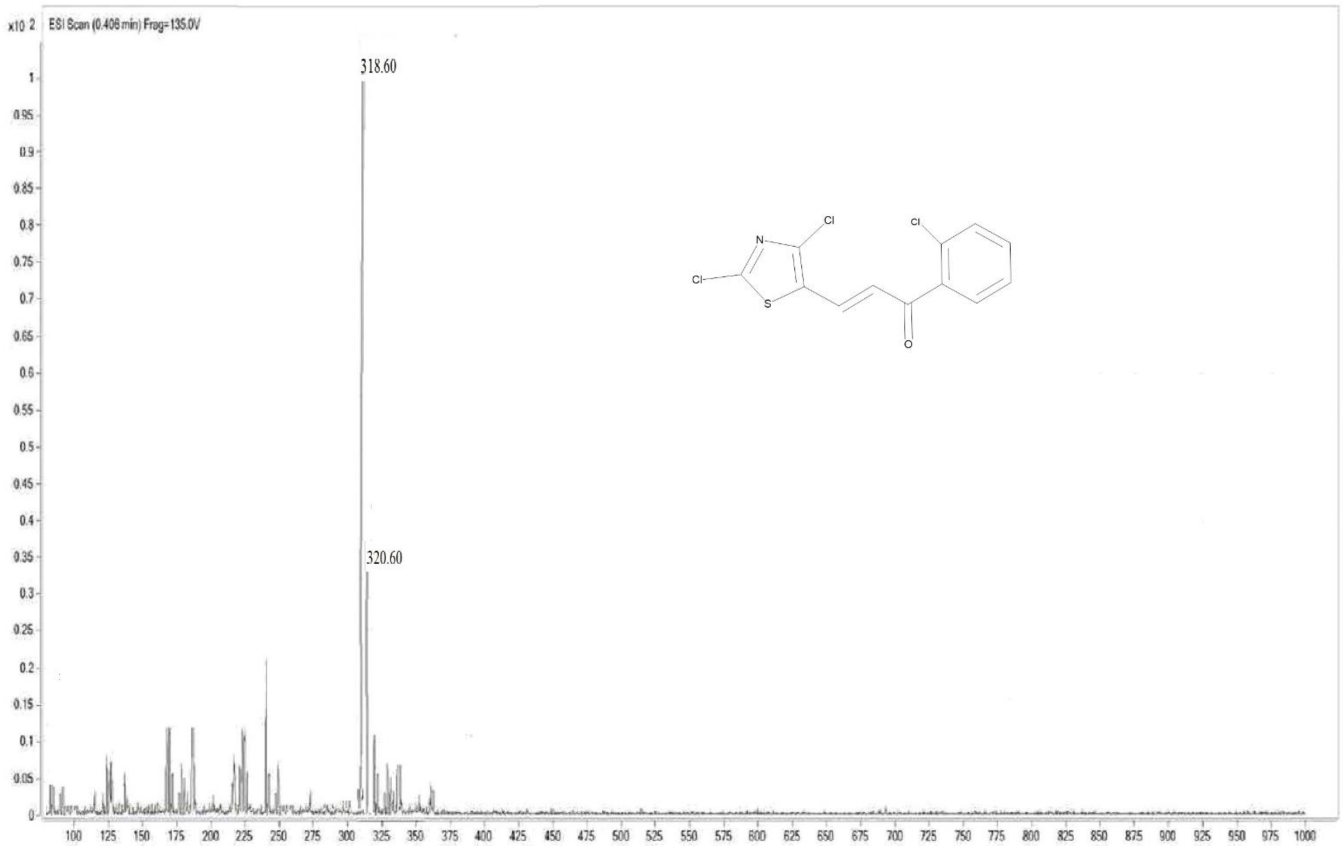

Figure S3. Mass Spectrum of compound 1

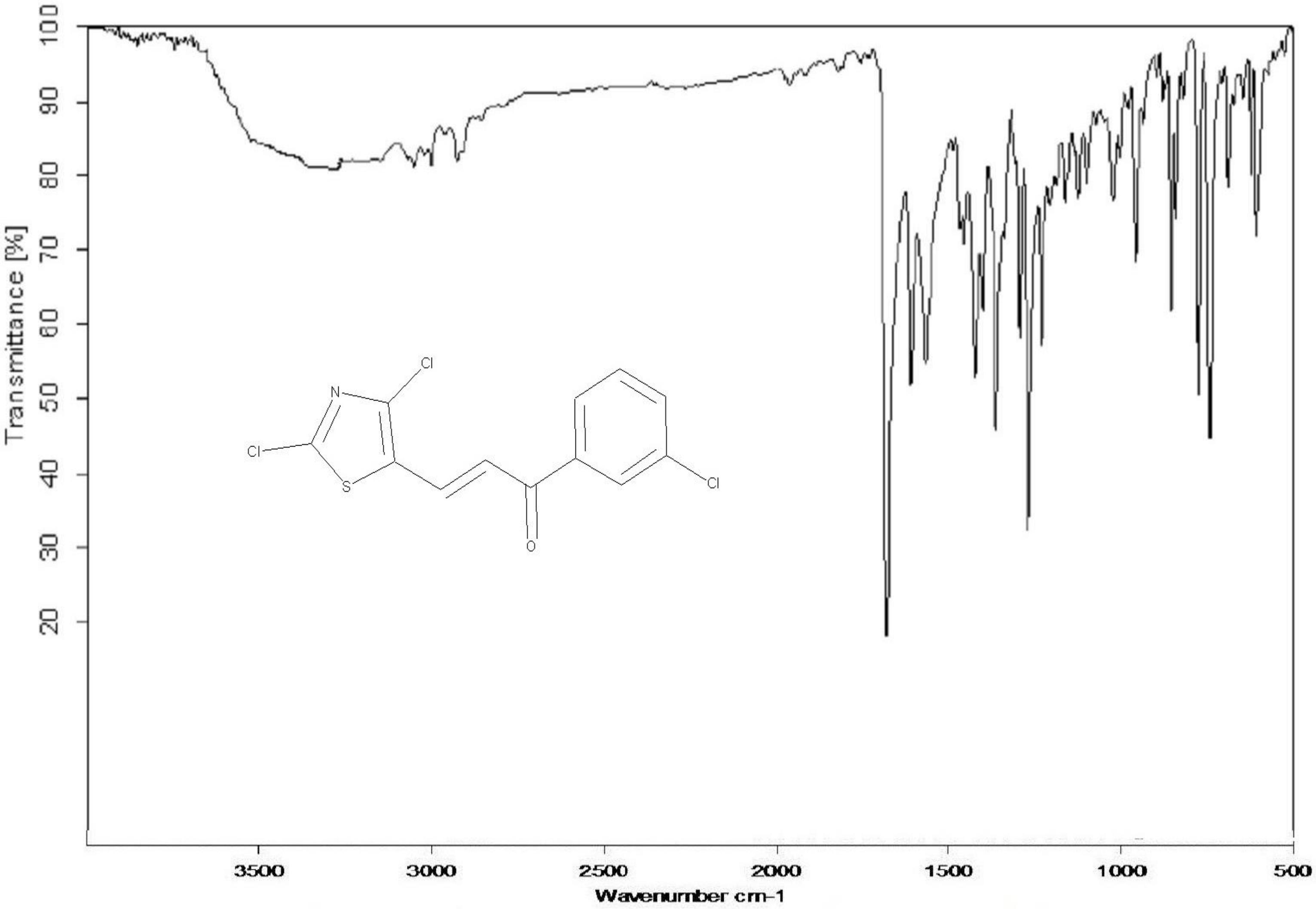

Figure S4. FT-IR Spectrum of compound 2

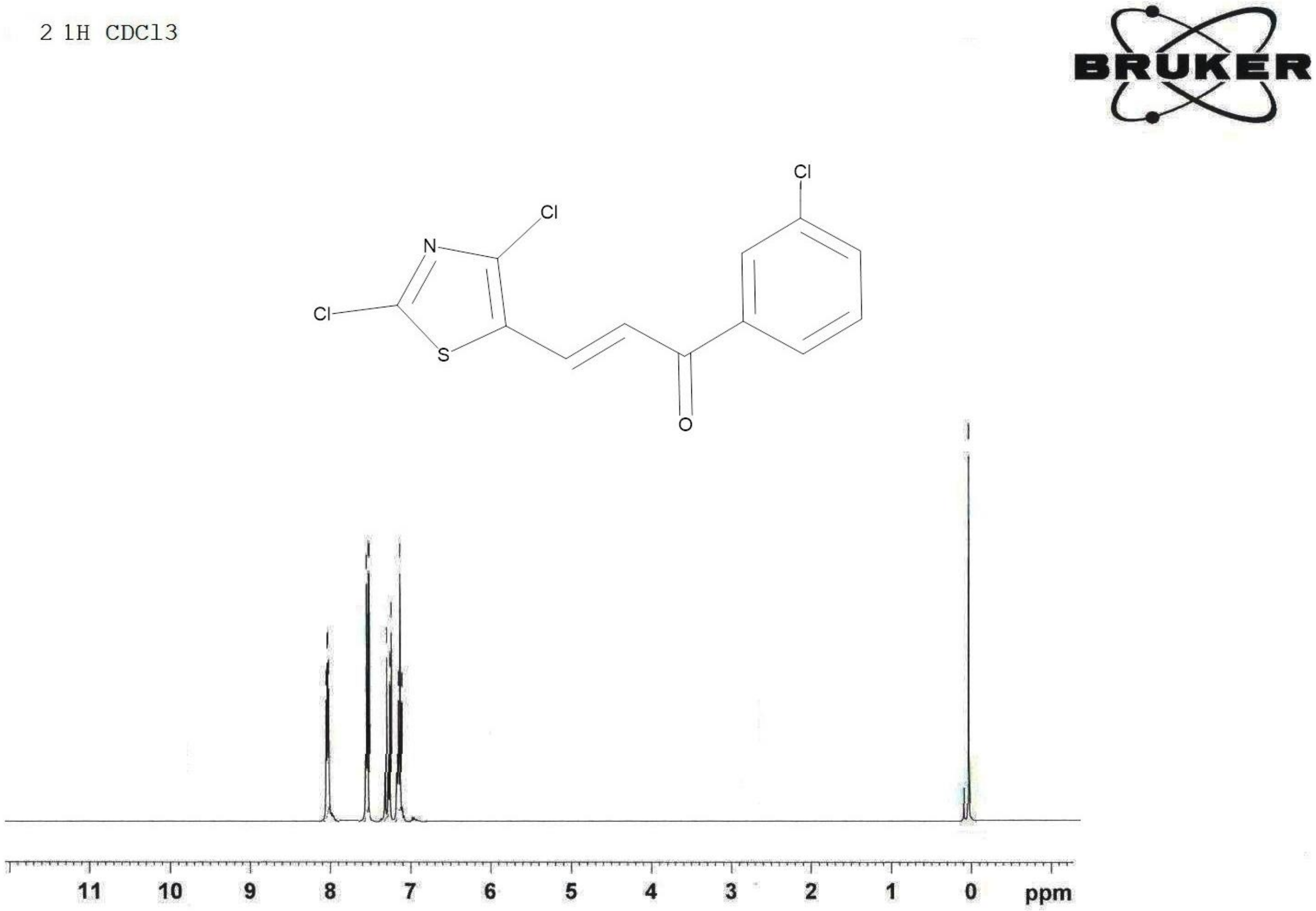

Figure S5. ^{1}H NMR Spectrum of compound 2

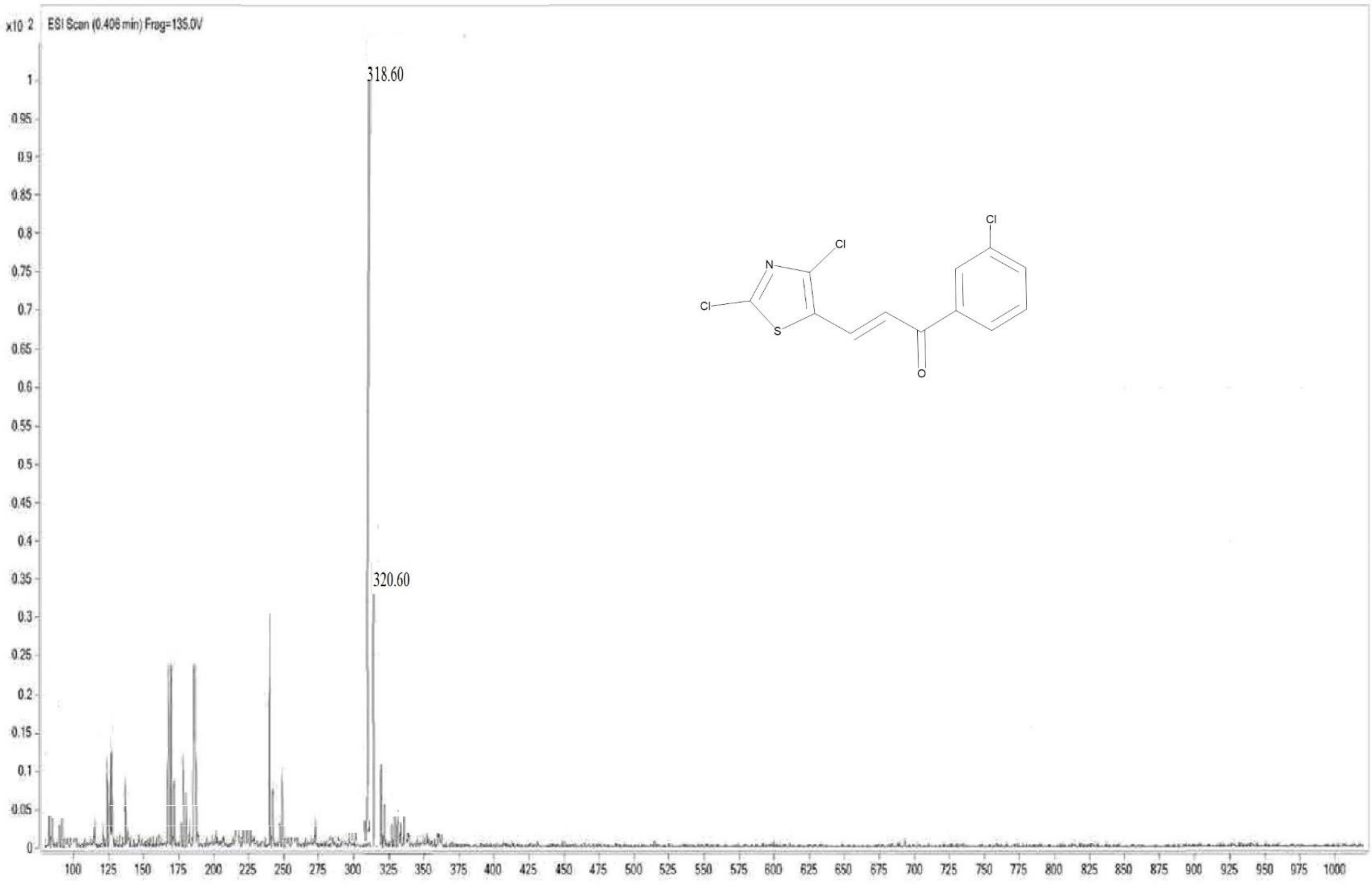

Figure S6. Mass Spectrum of compound 2

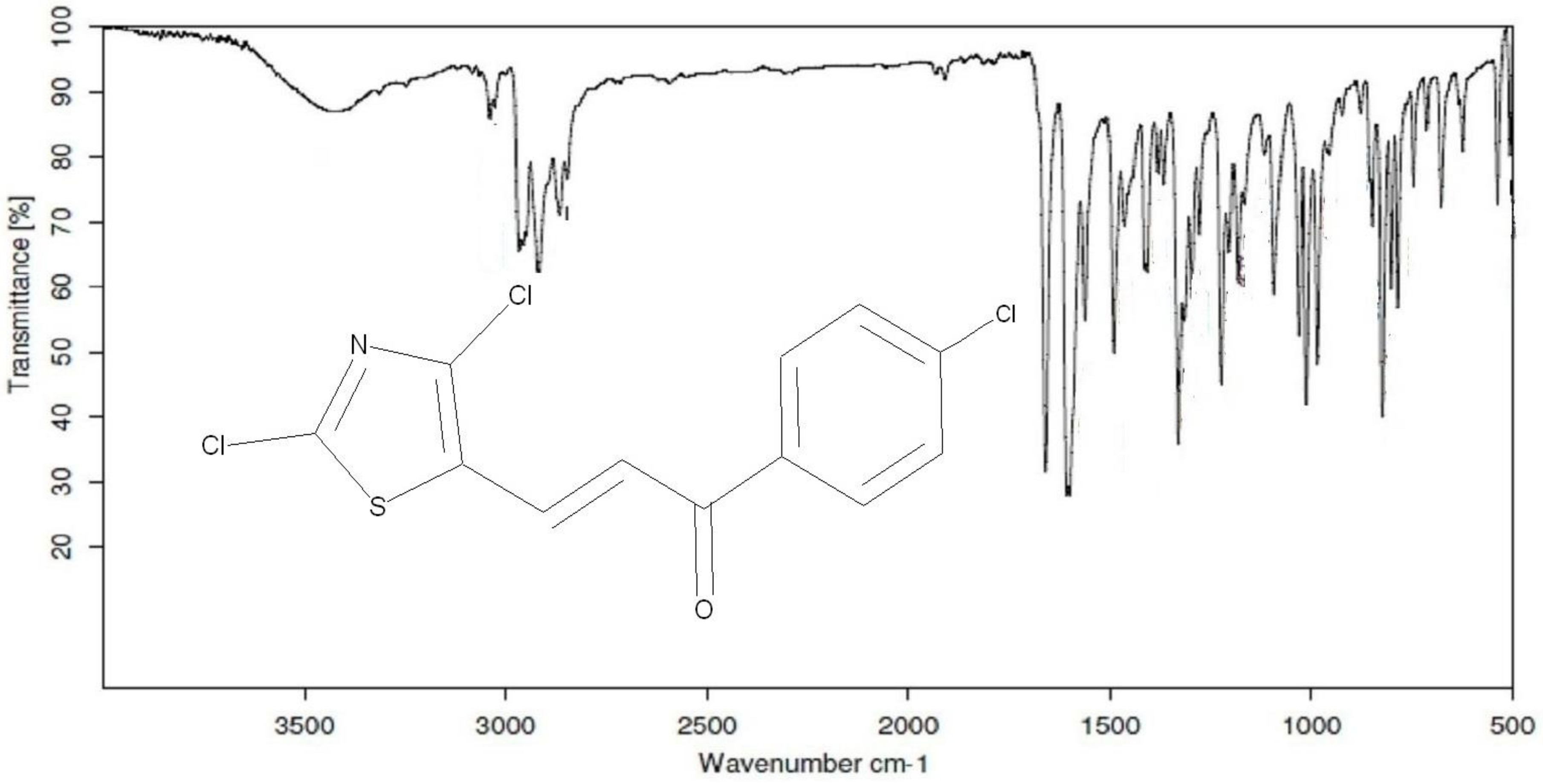

Figure S7. FT-IR Spectrum of compound 3

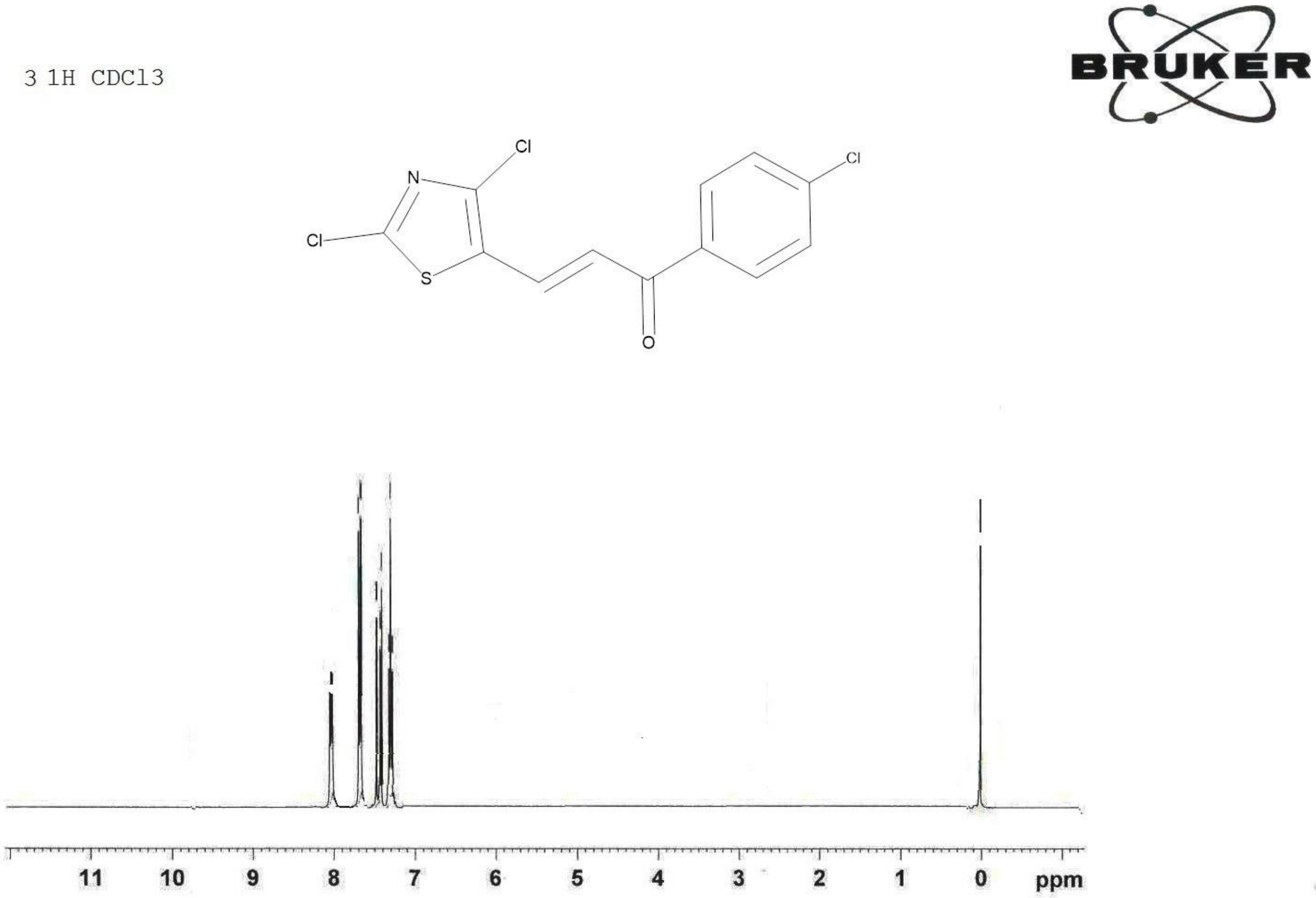

Figure S8. ^{1}H NMR Spectrum of compound 3

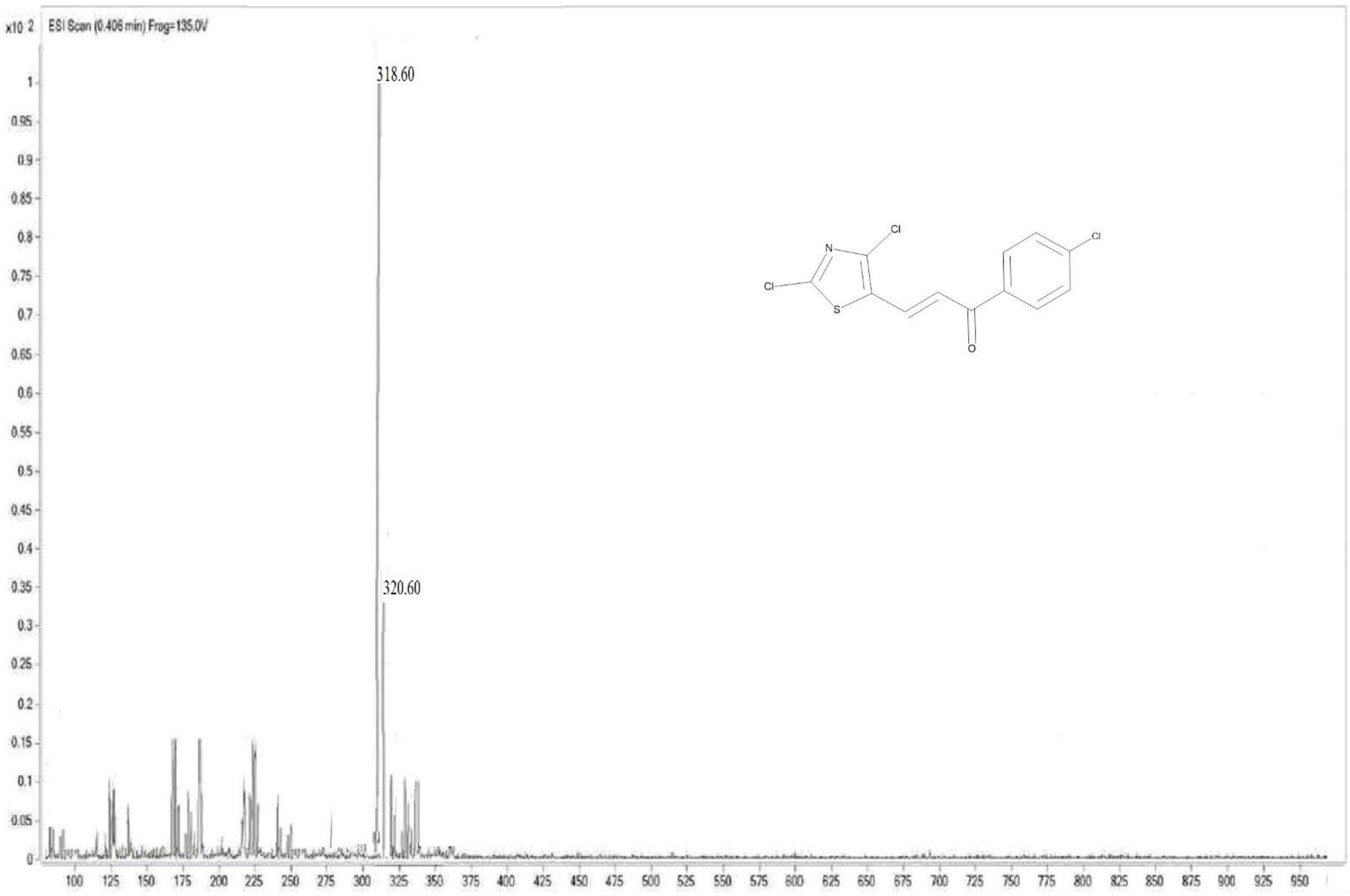

Figure S9. Mass Spectrum of compound 3

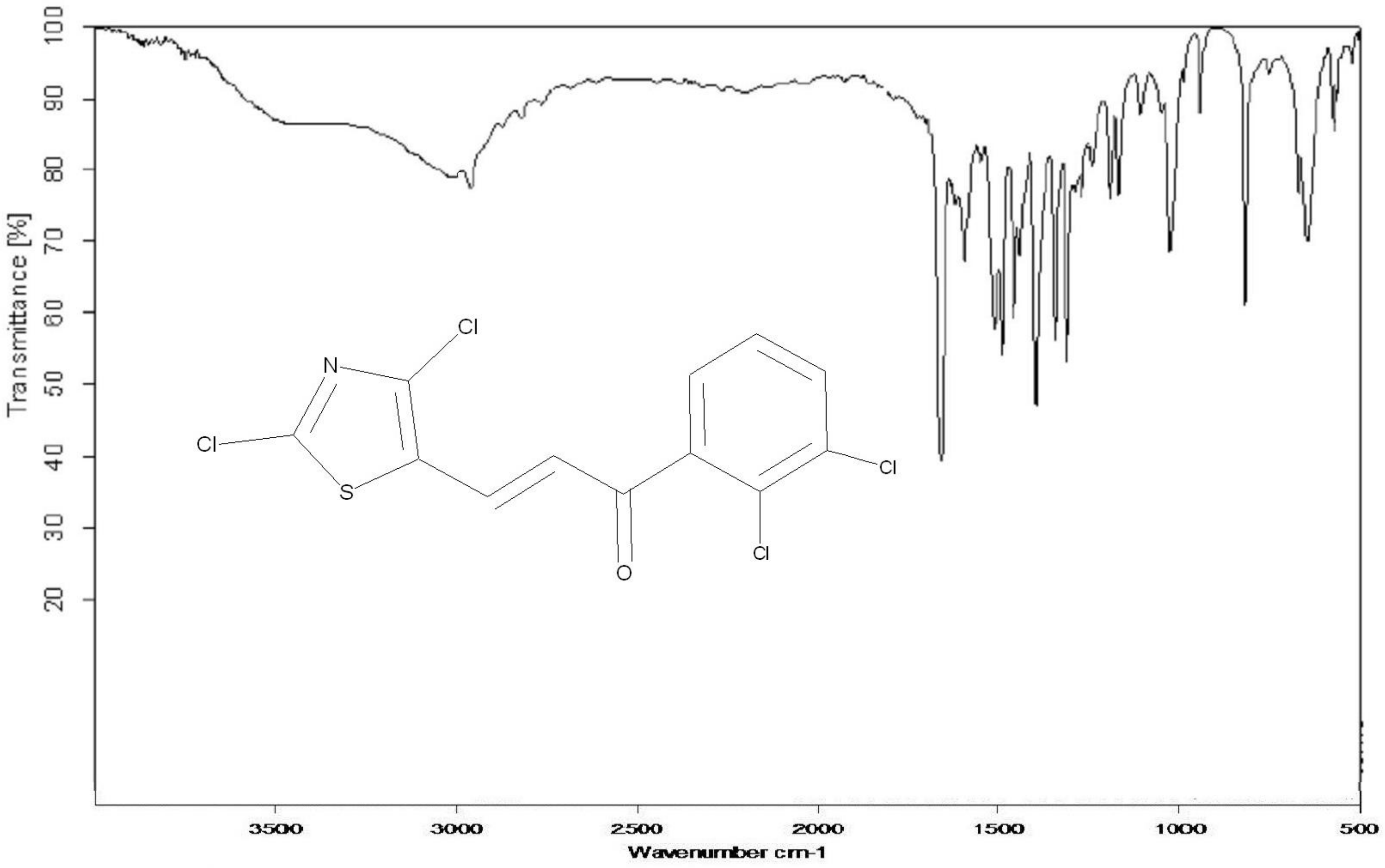

Figure S10. FT-IR Spectrum of compound 4

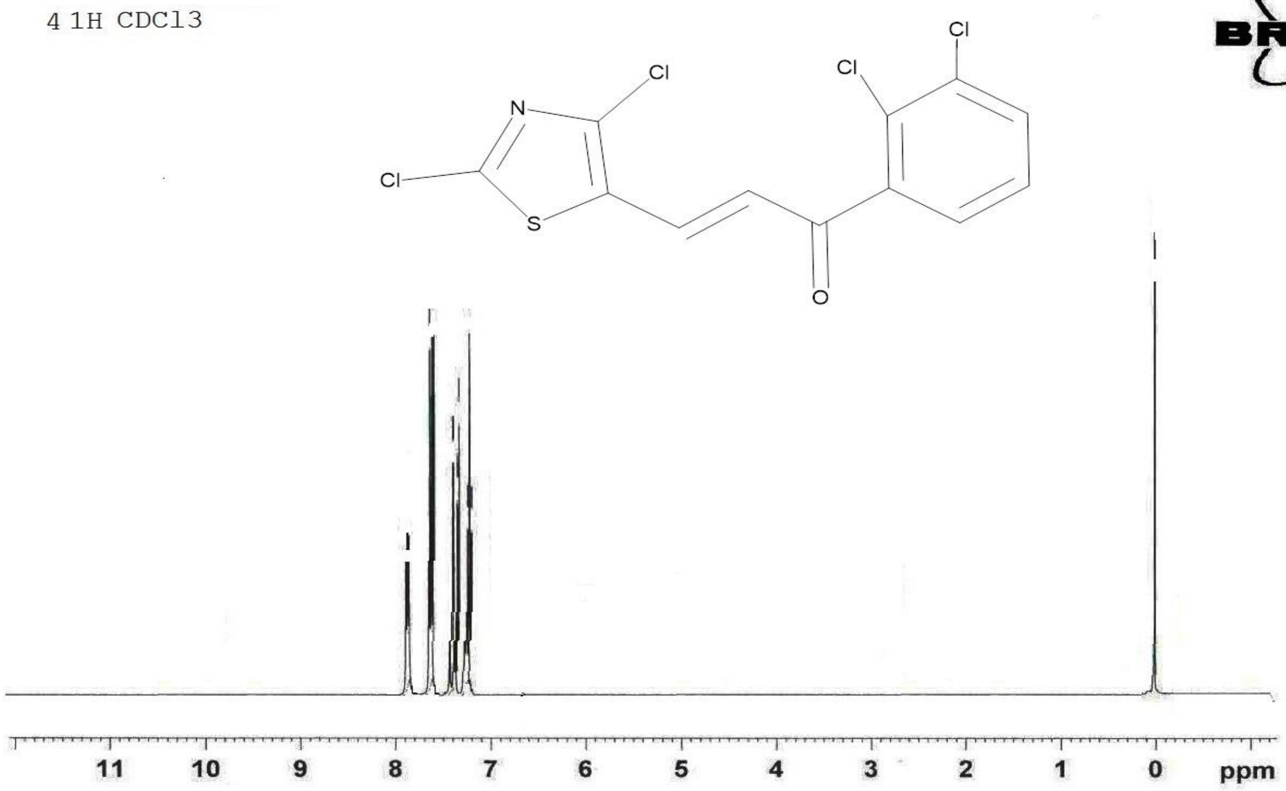

Figure S11. **¹H NMR Spectrum of compound 4**

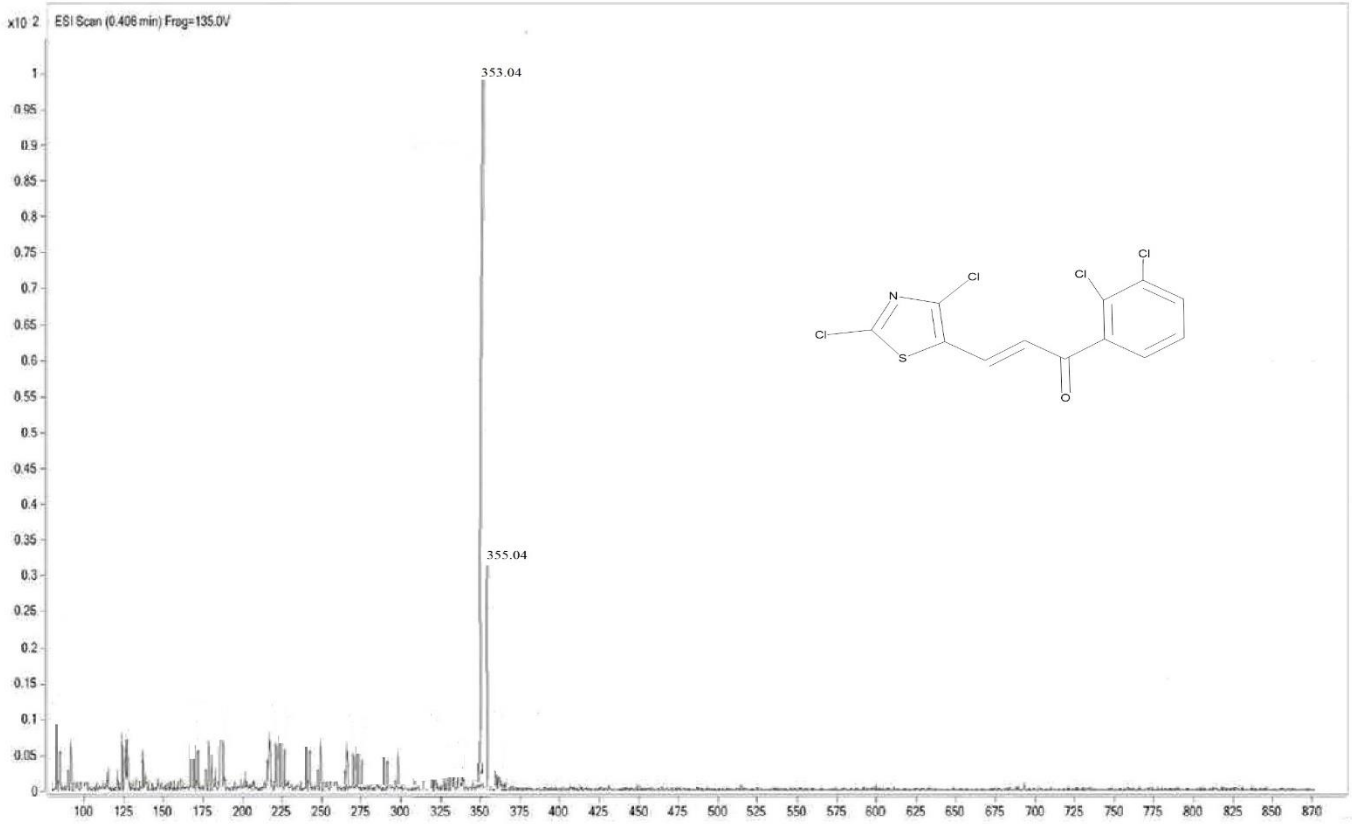

Figure S12. Mass Spectrum of compound 4

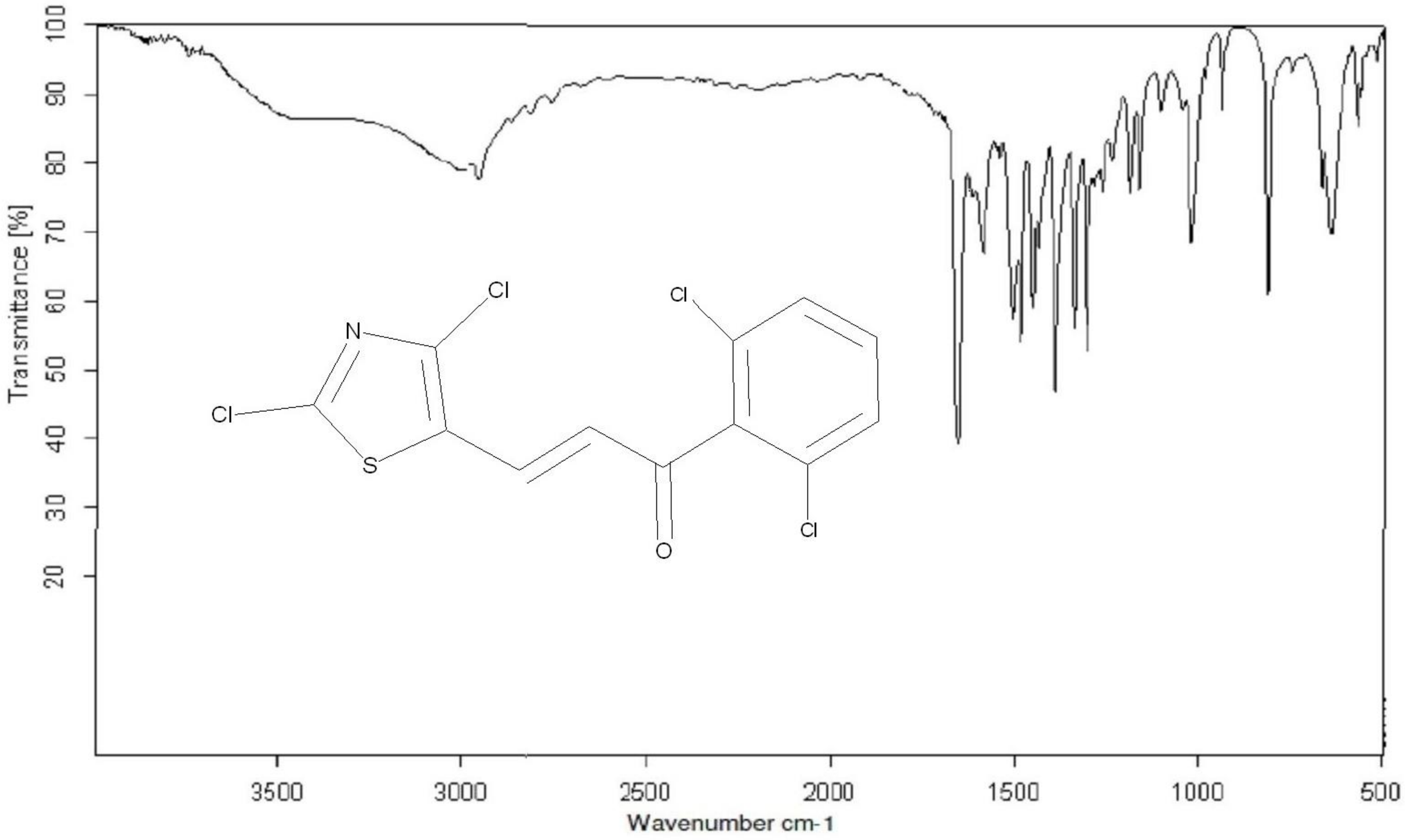

Figure S13. FT-IR Spectrum of compound 5

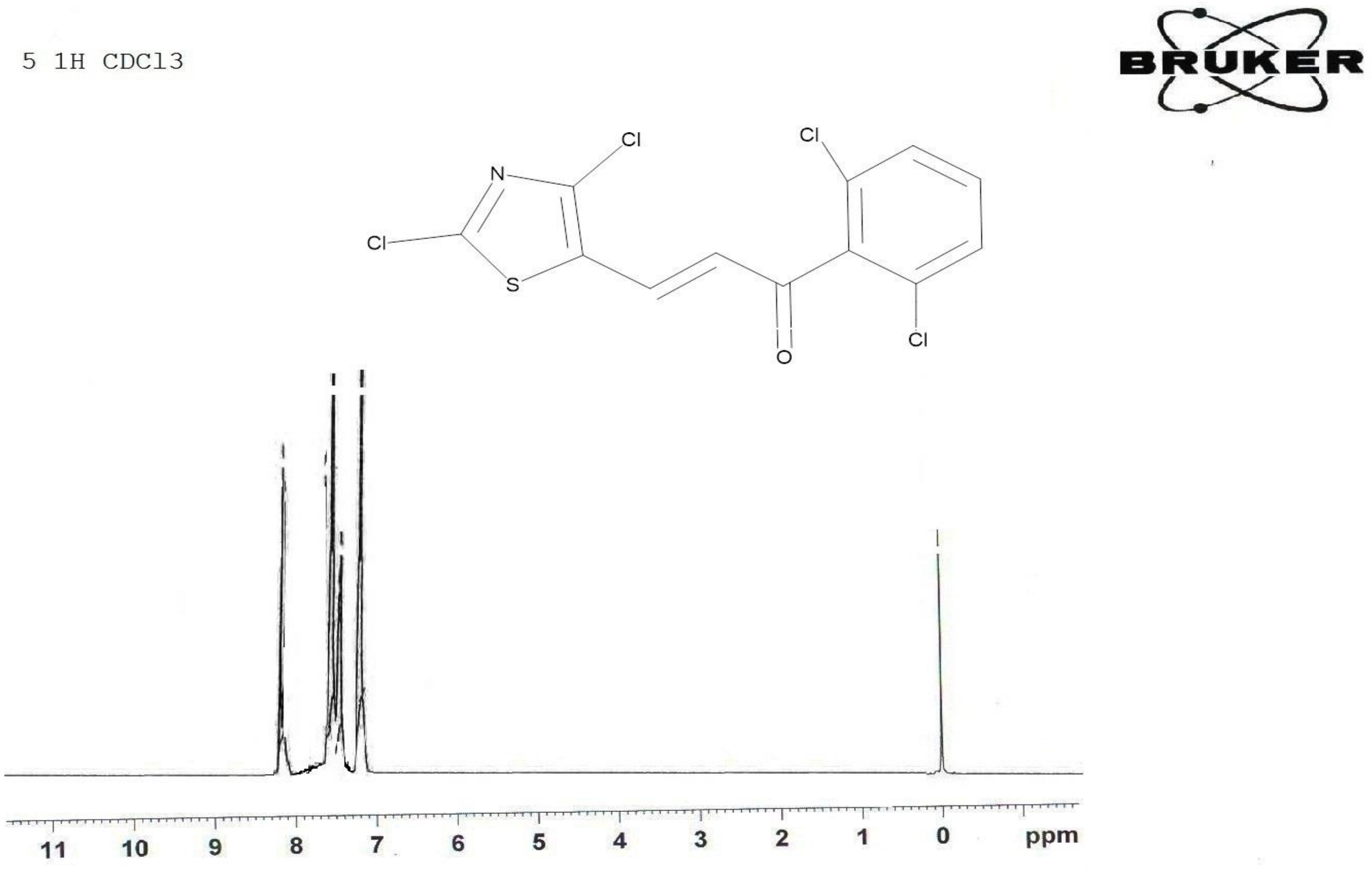

Figure S14. ^{1}H **NMR Spectrum of compound 5**

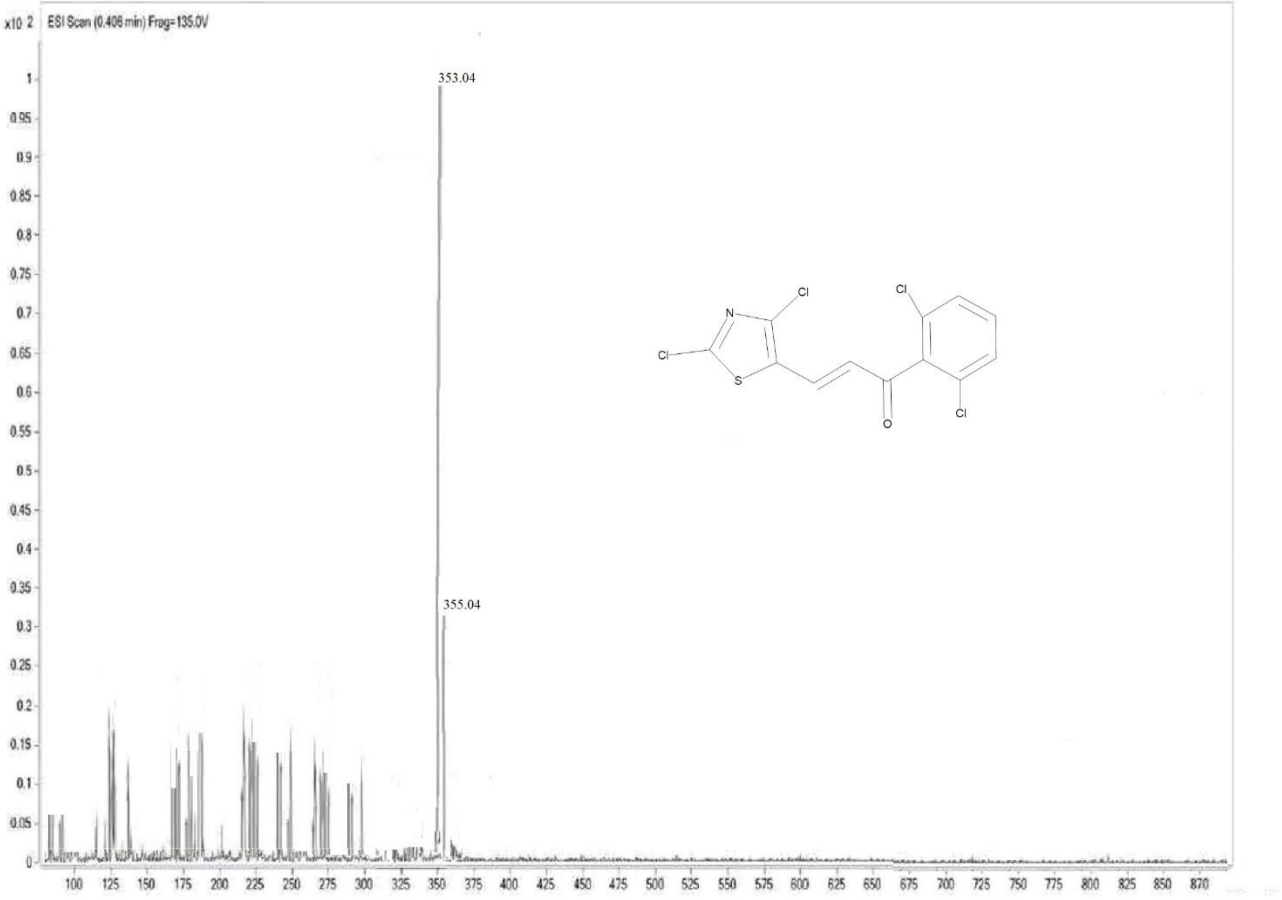

Figure S15. Mass Spectrum of compound 5

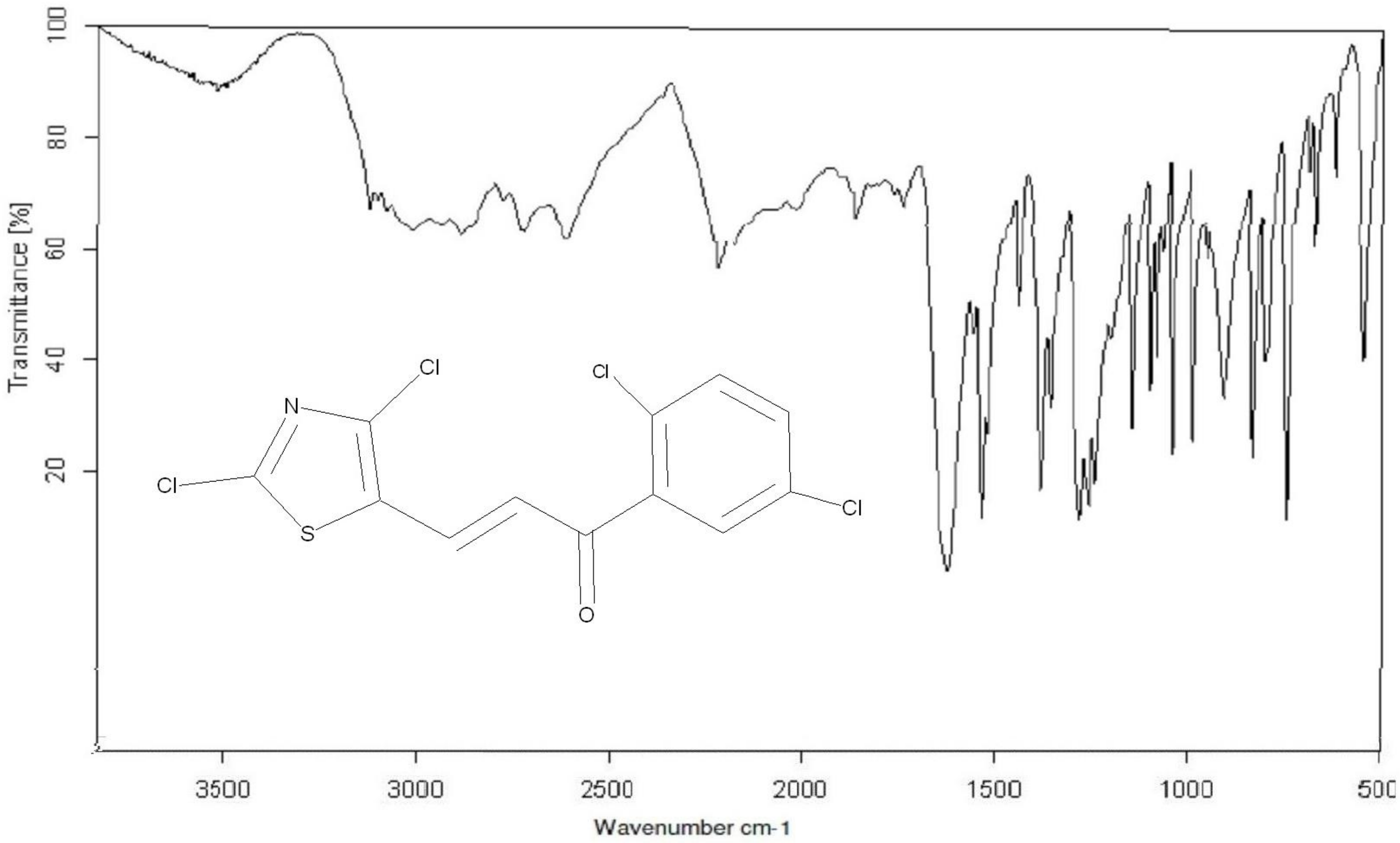

Figure S16. FT-IR Spectrum of compound 6

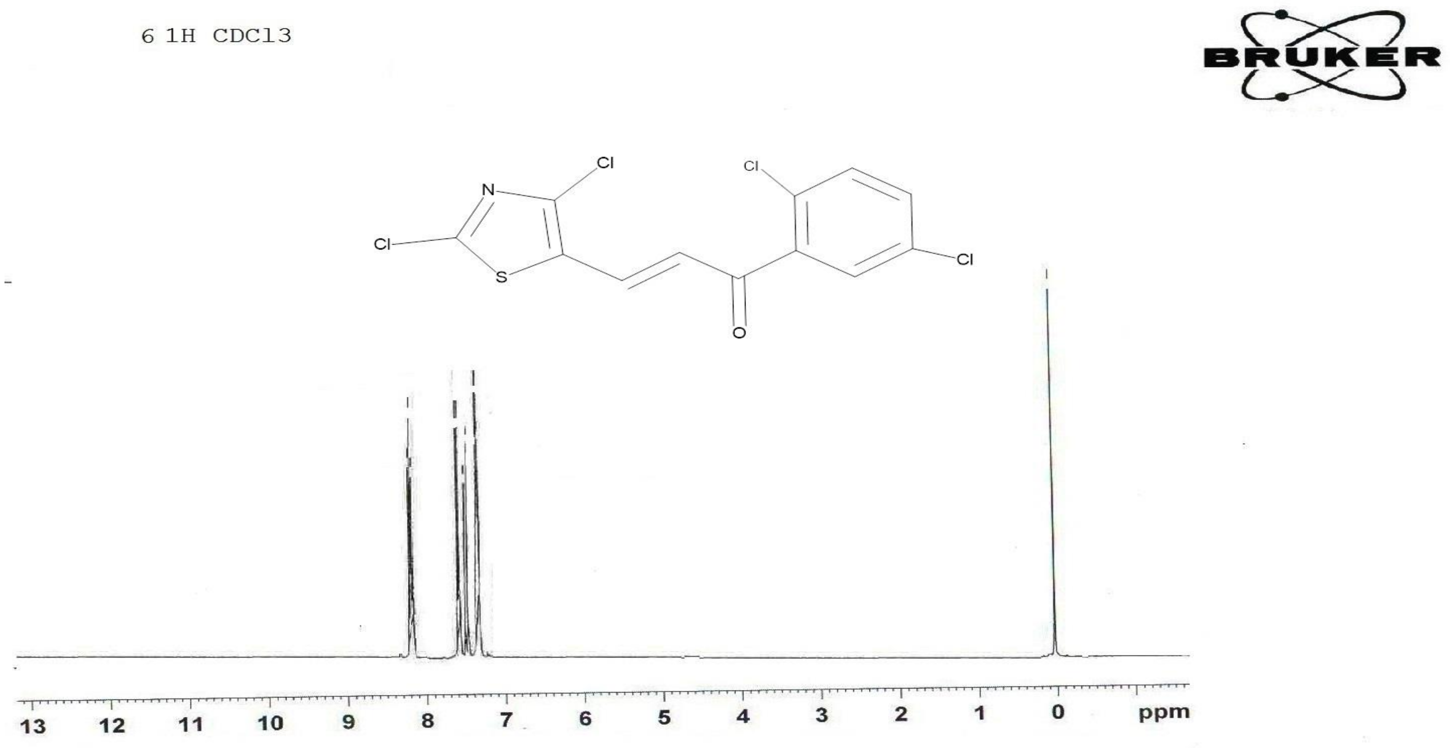

Figure S17. ^{1}H NMR Spectrum of compound 6

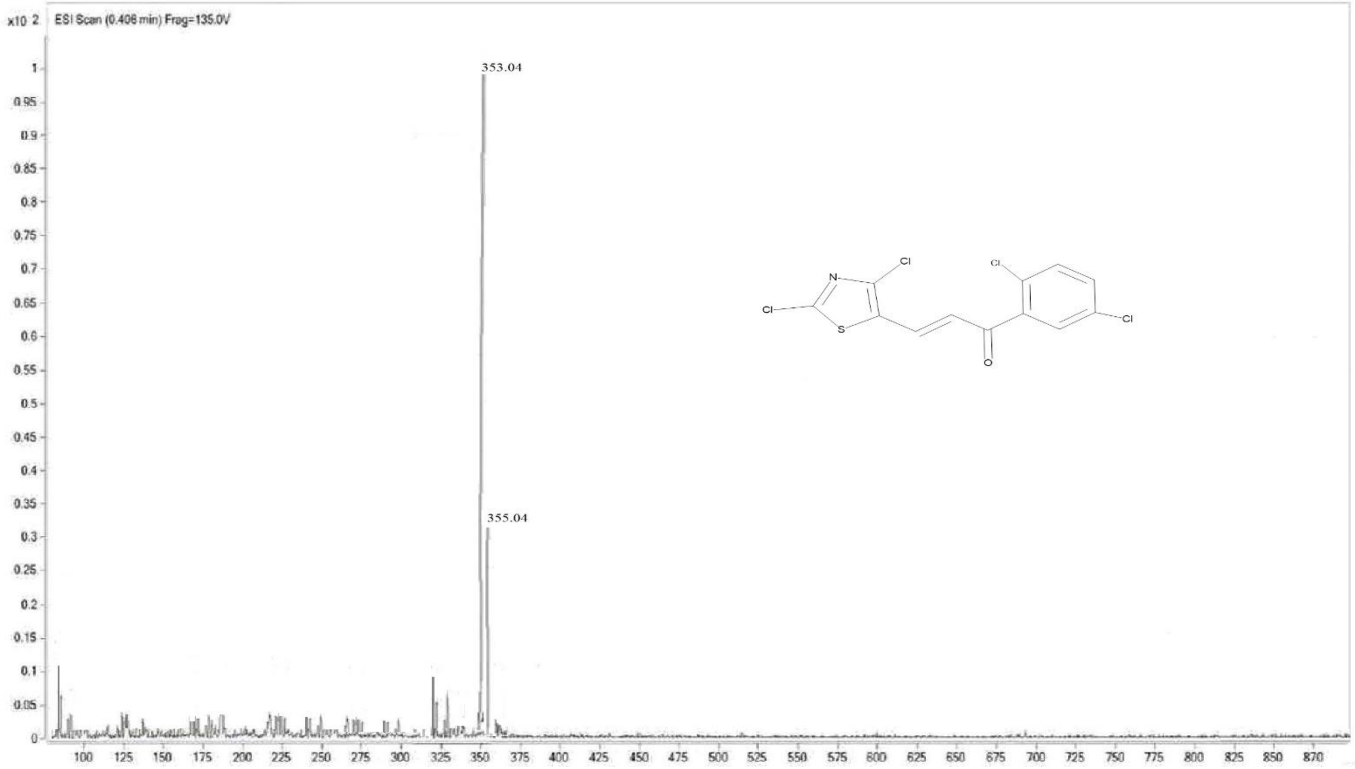

Figure S18. Mass Spectrum of compound 6

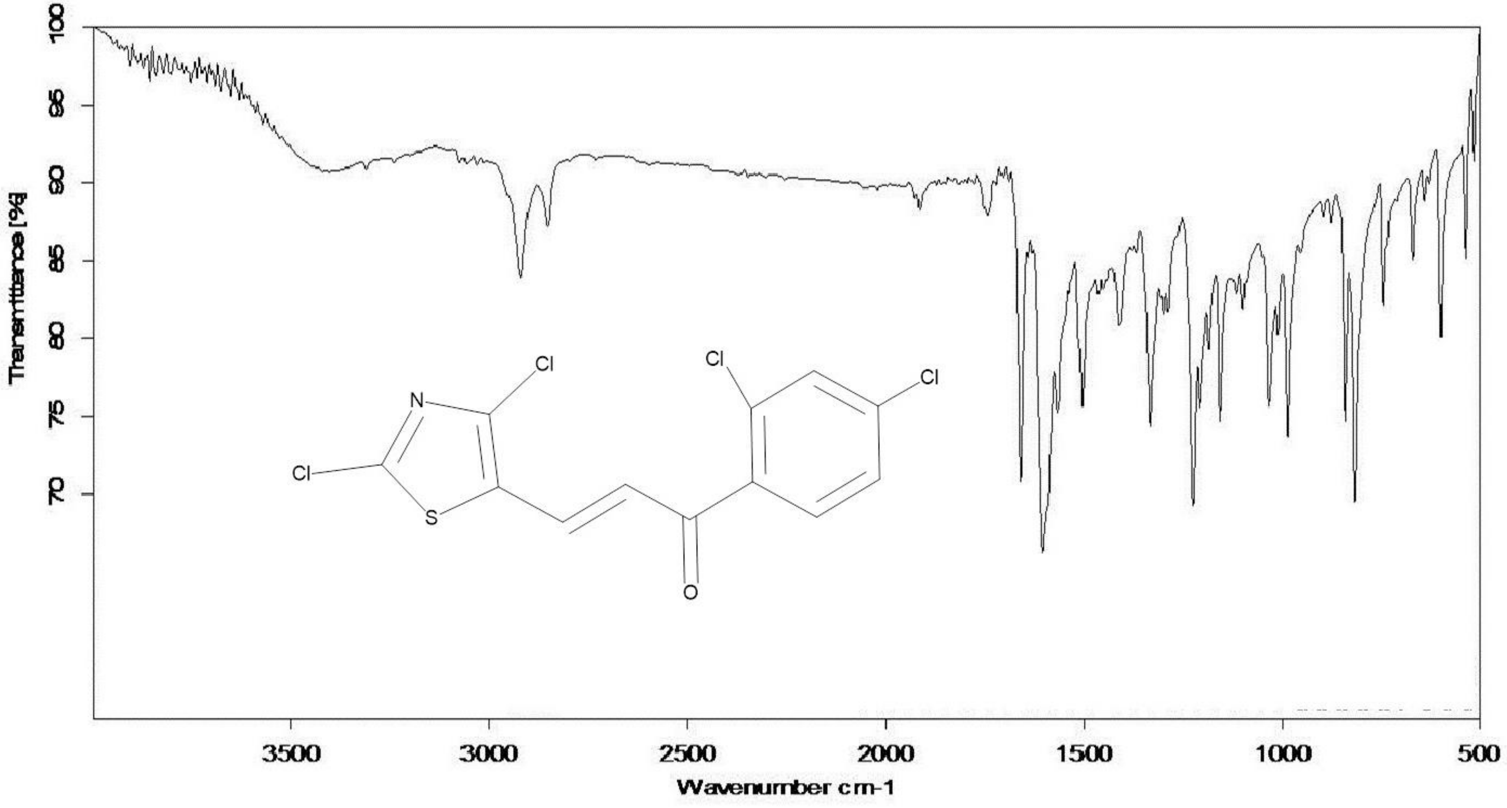

Figure S19. FT-IR Spectrum of compound 7

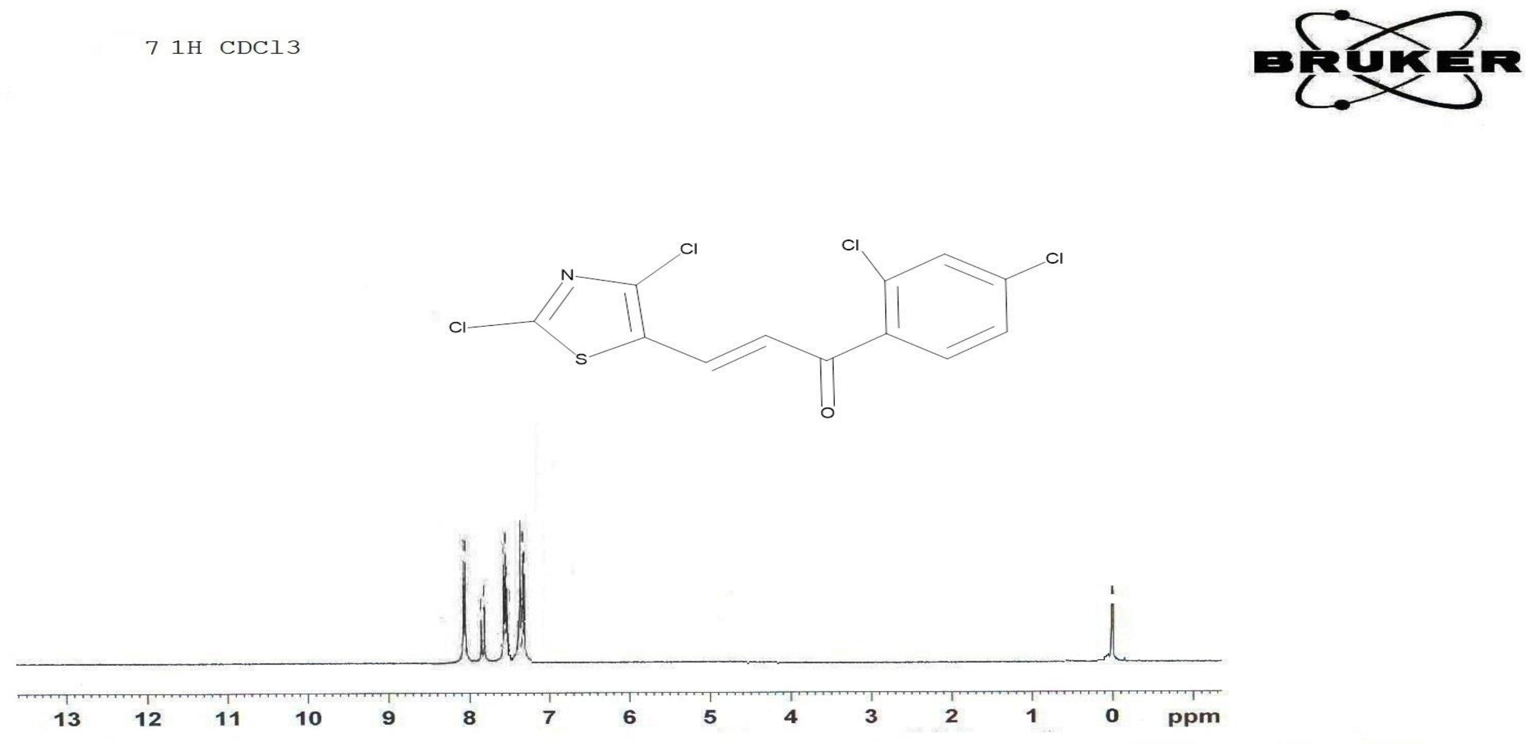

Figure S20. ^{1}H NMR Spectrum of compound 7

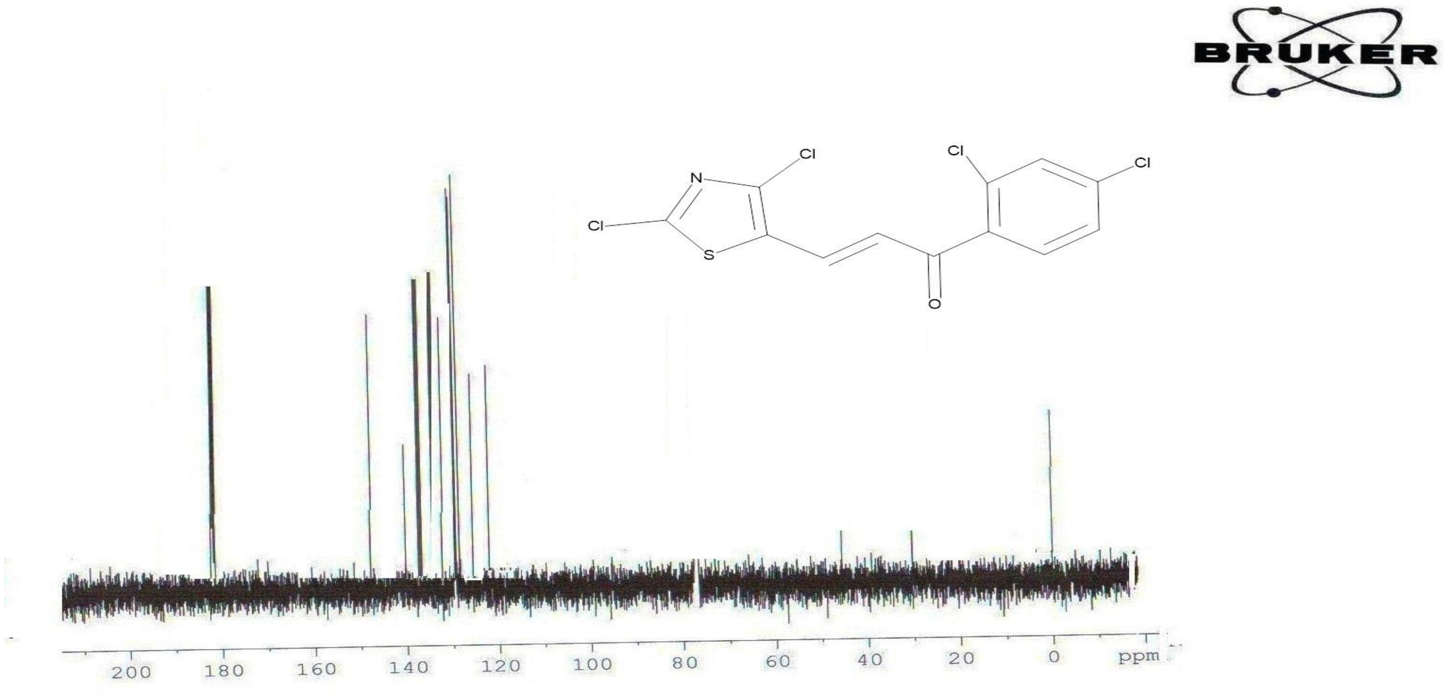

Figure S21. ^{13}C NMR Spectrum of compound 7

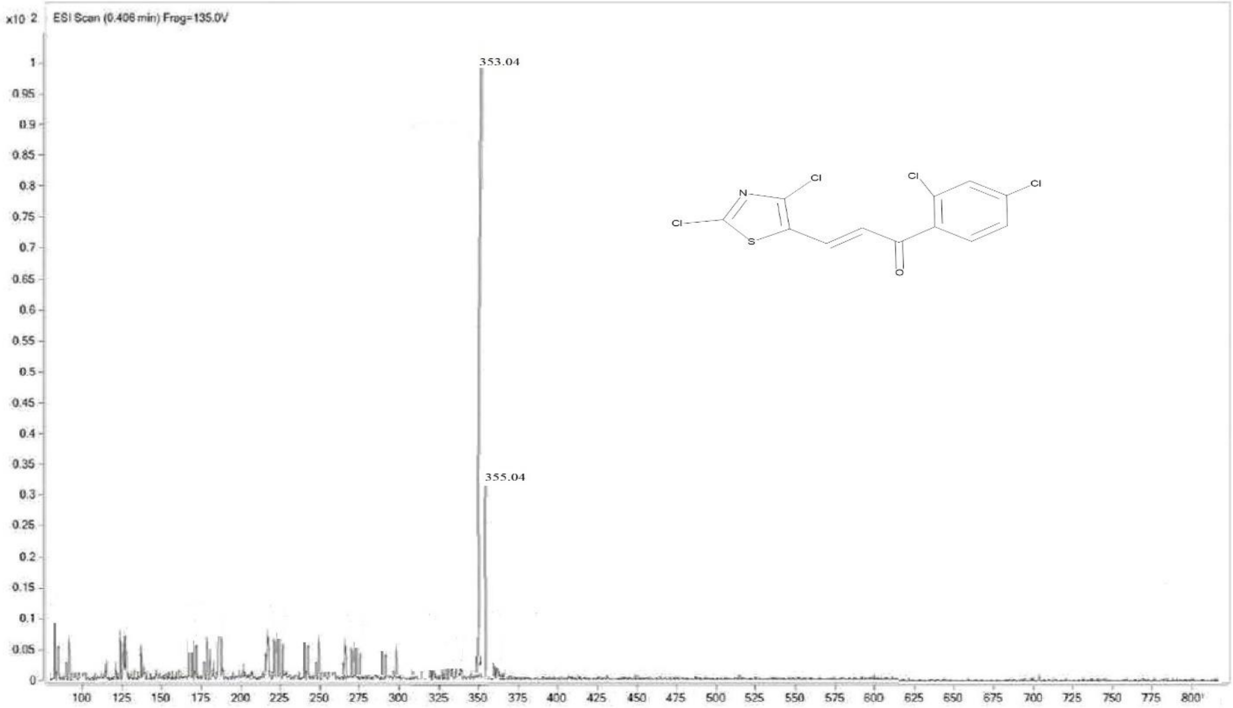

Figure S22. Mass Spectrum of compound 7

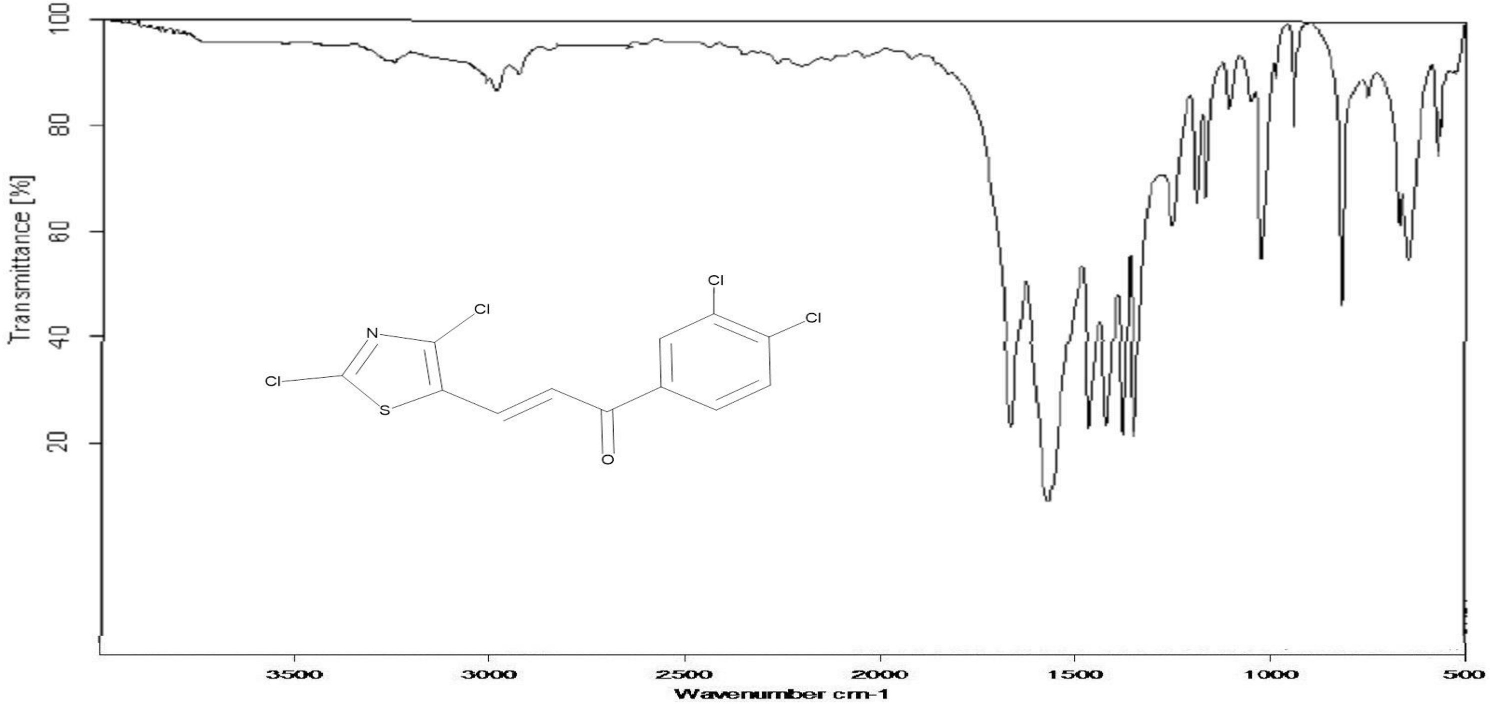

Figure 23. FT-IR Spectrum of compound 8

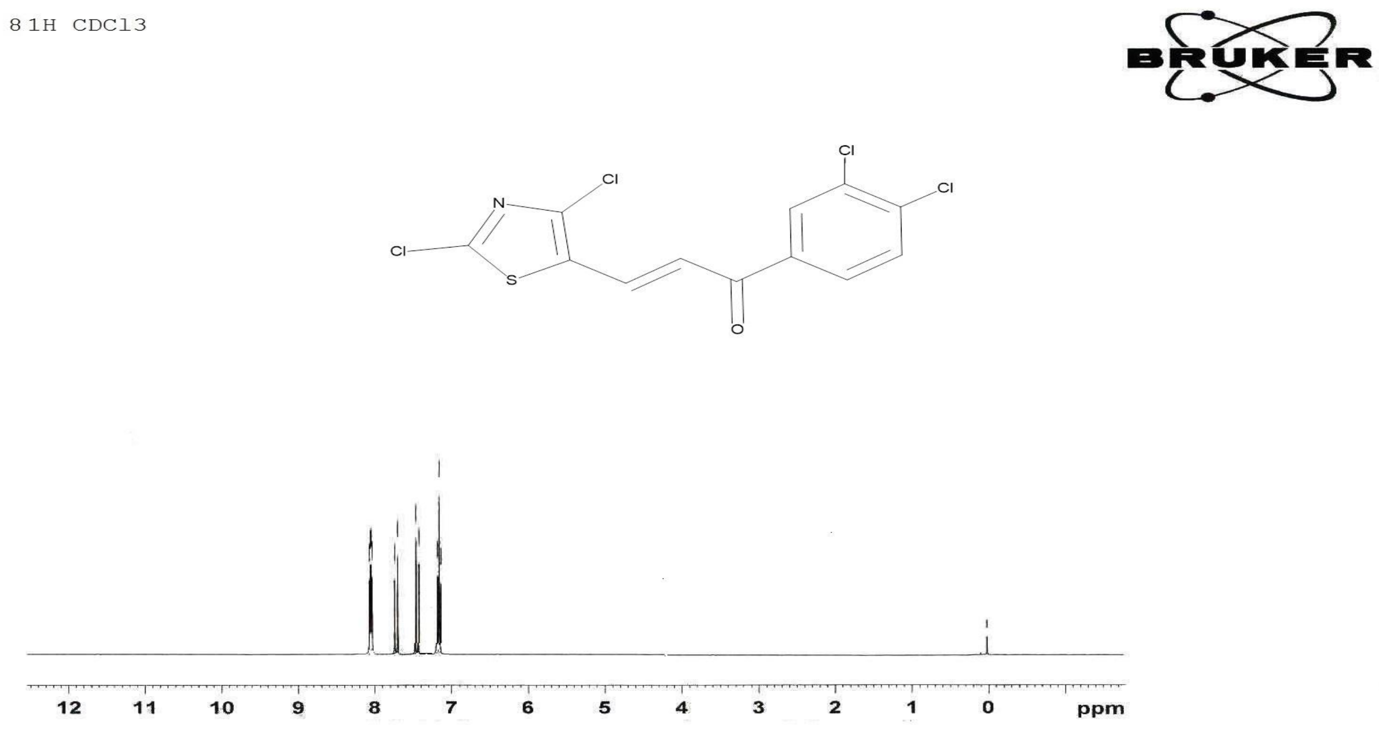

Figure S24. ^{1}H NMR Spectrum of compound 8

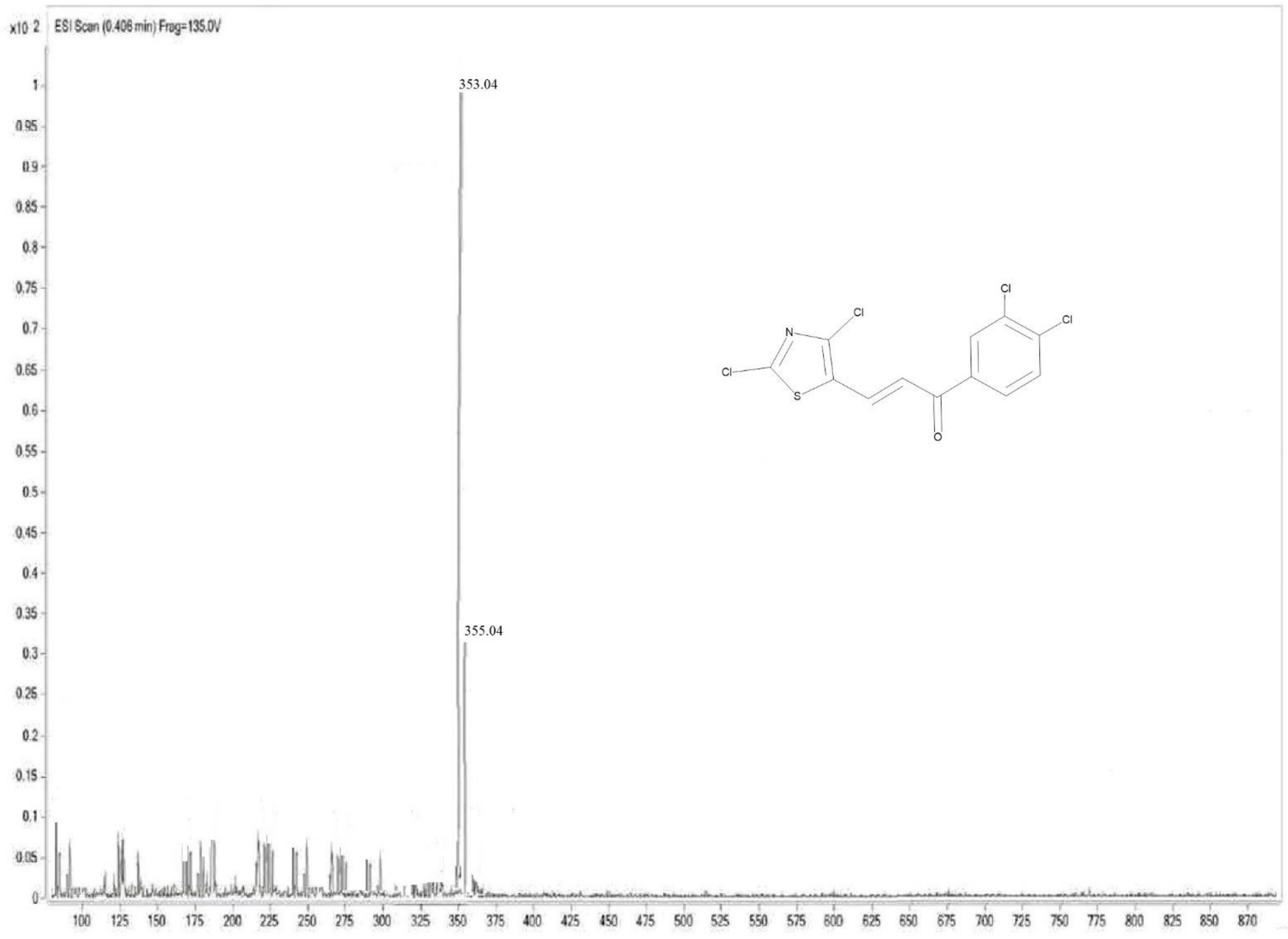

Figure S25. Mass Spectrum of compound 8

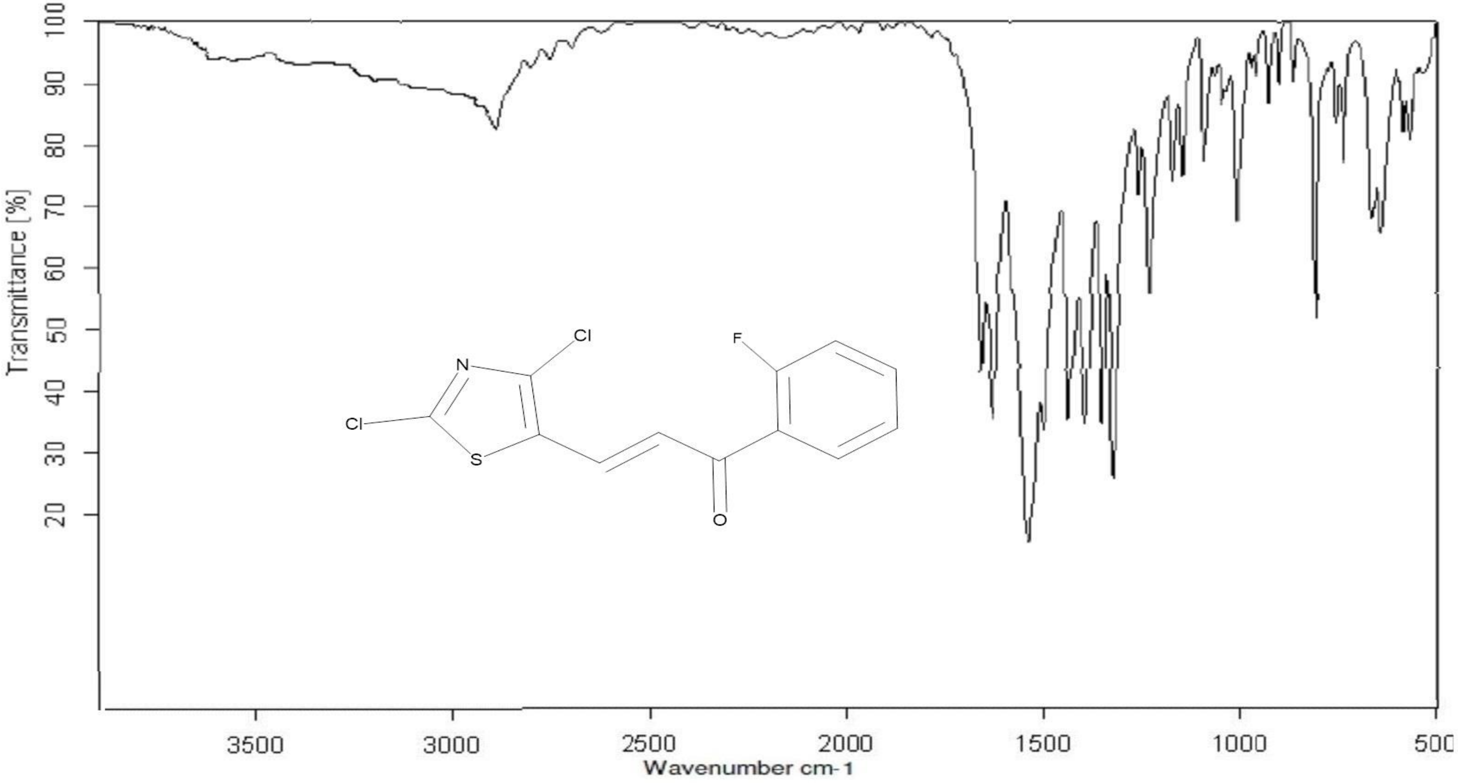

Figure S26. FT-IR Spectrum of compound 9

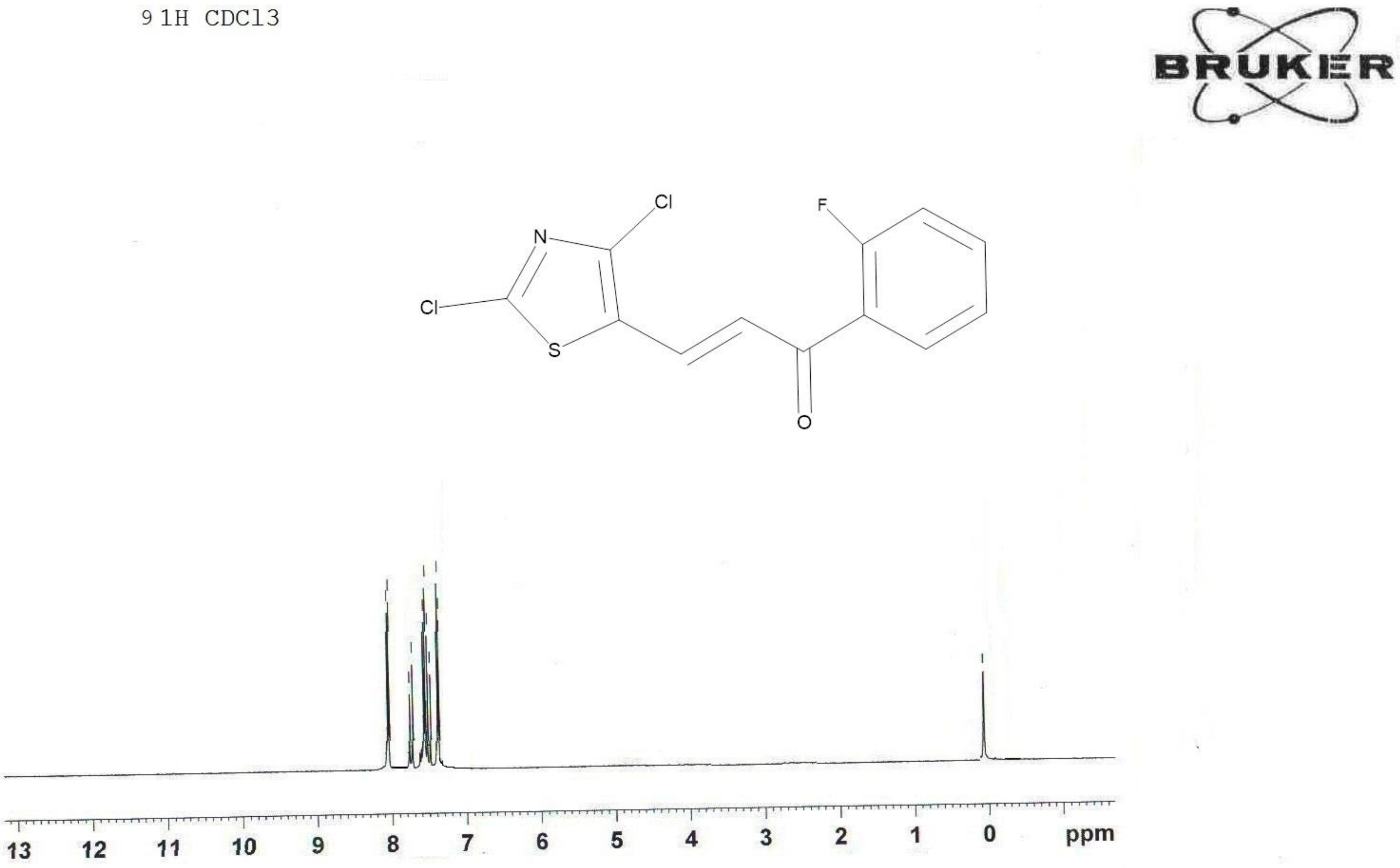

Figure S27. **^{1}H NMR Spectrum of compound 9**

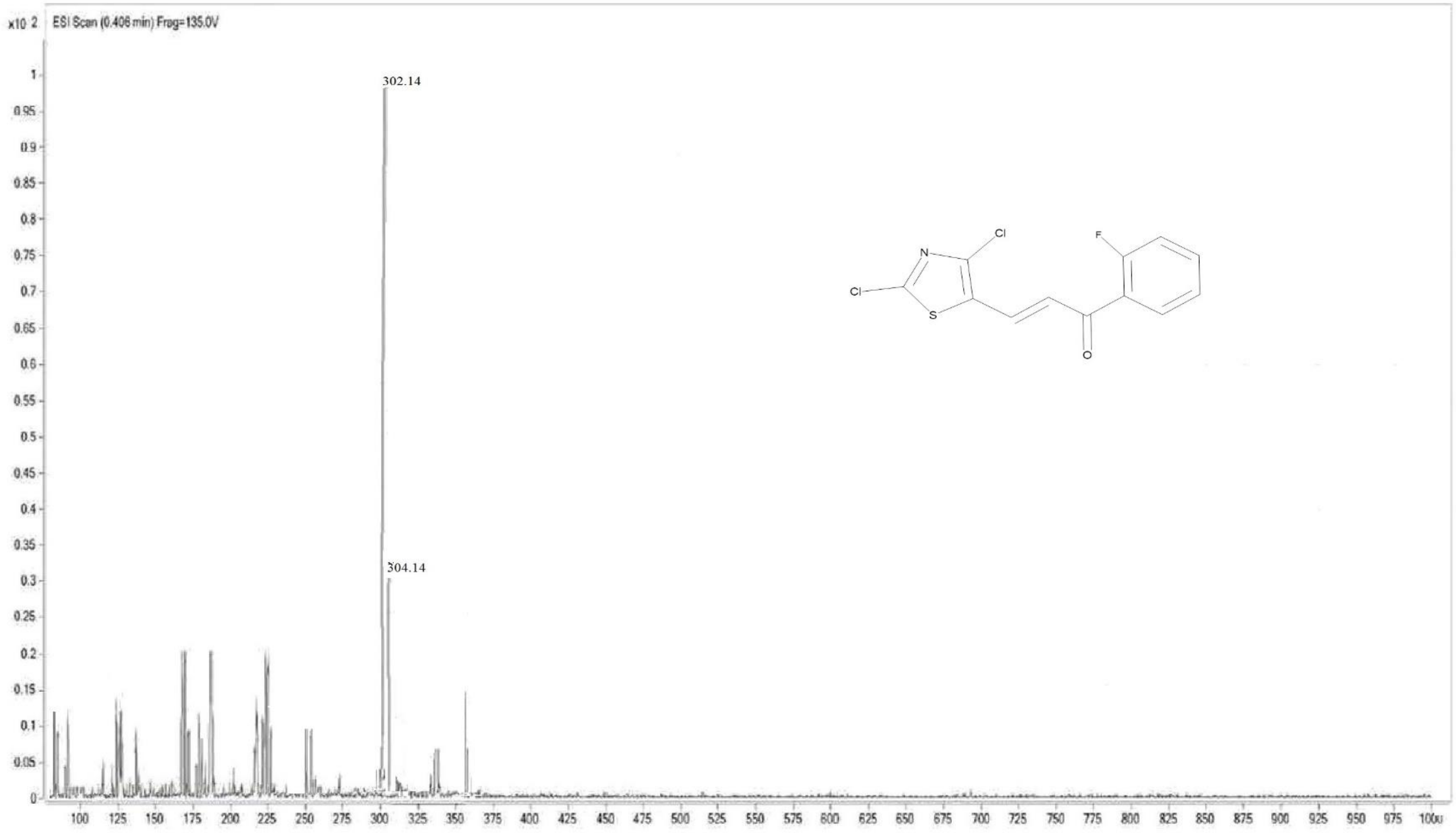

Figure S28. Mass Spectrum of compound 9

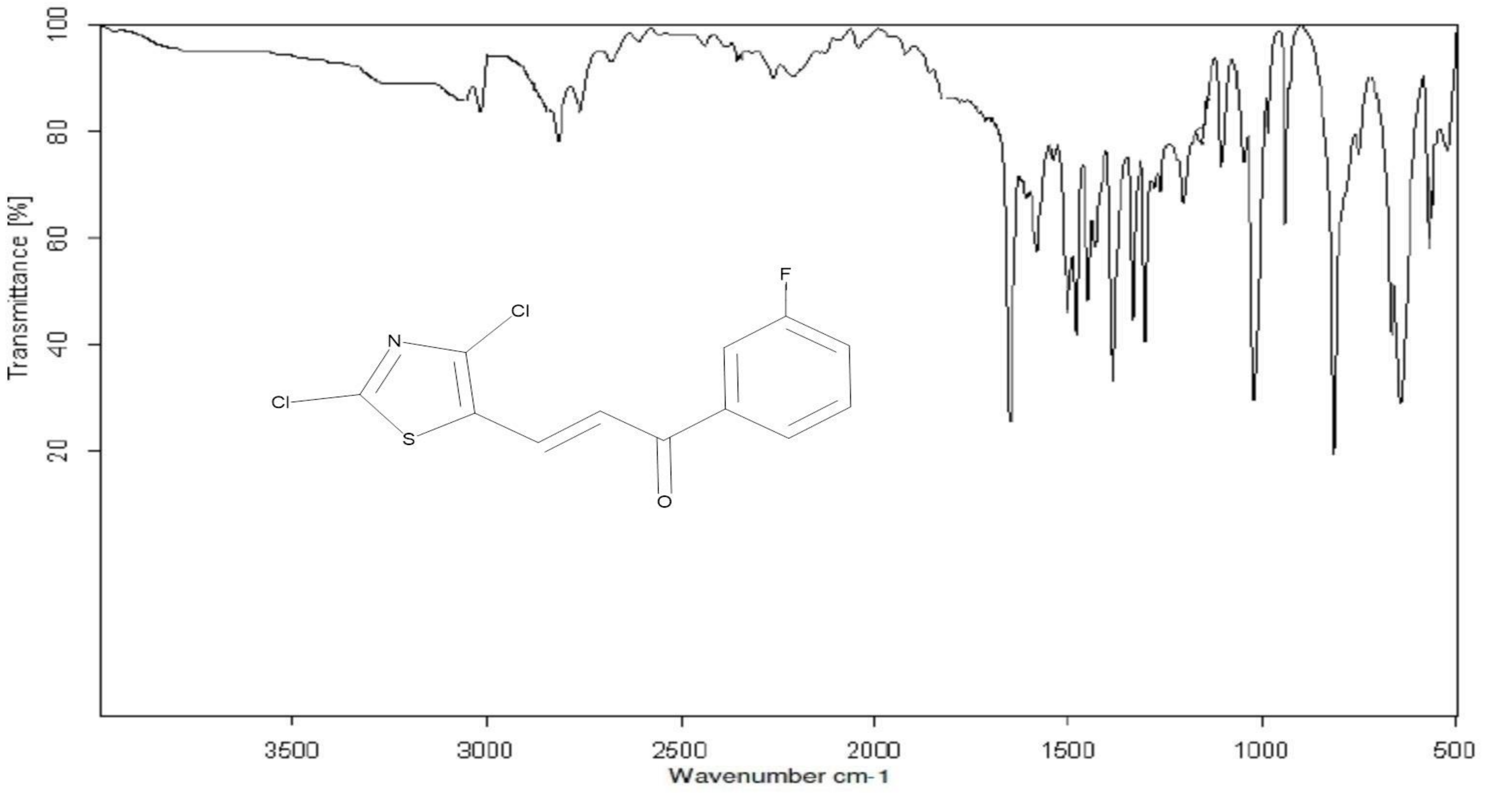

Figure S29. FT-IR Spectrum of compound 10

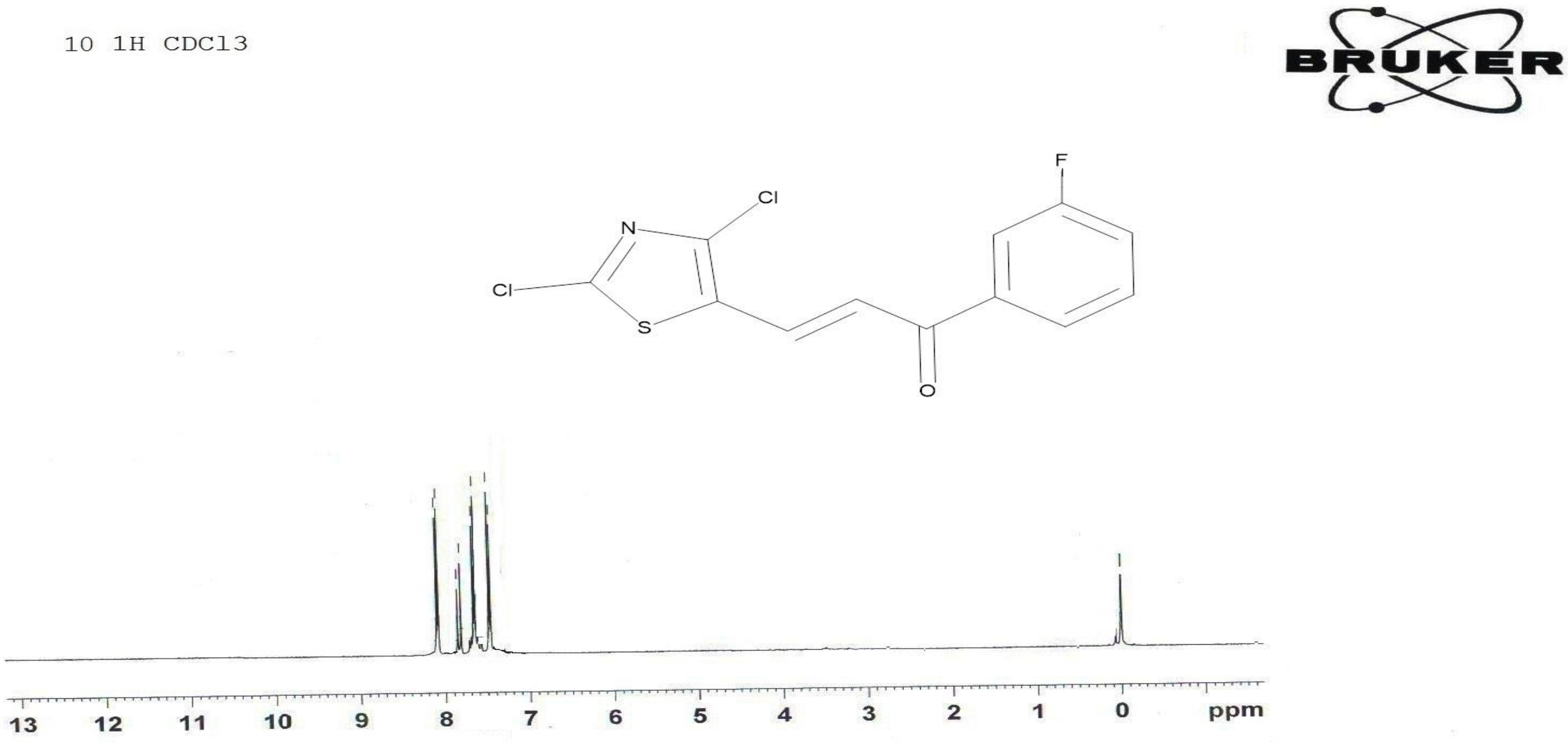

Figure S30. ¹H NMR Spectrum of compound 10

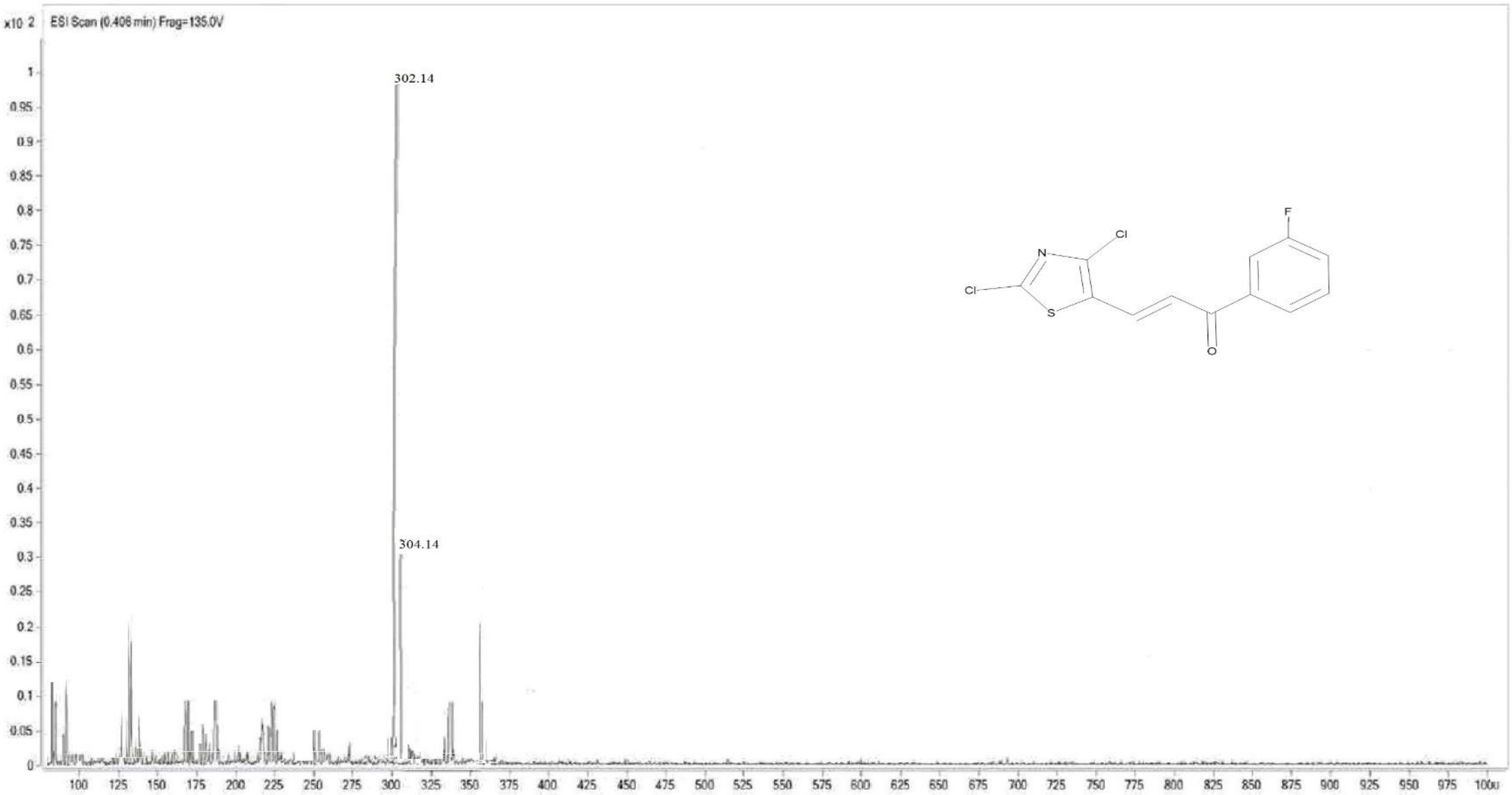

Figure S31. Mass Spectrum of compound 10

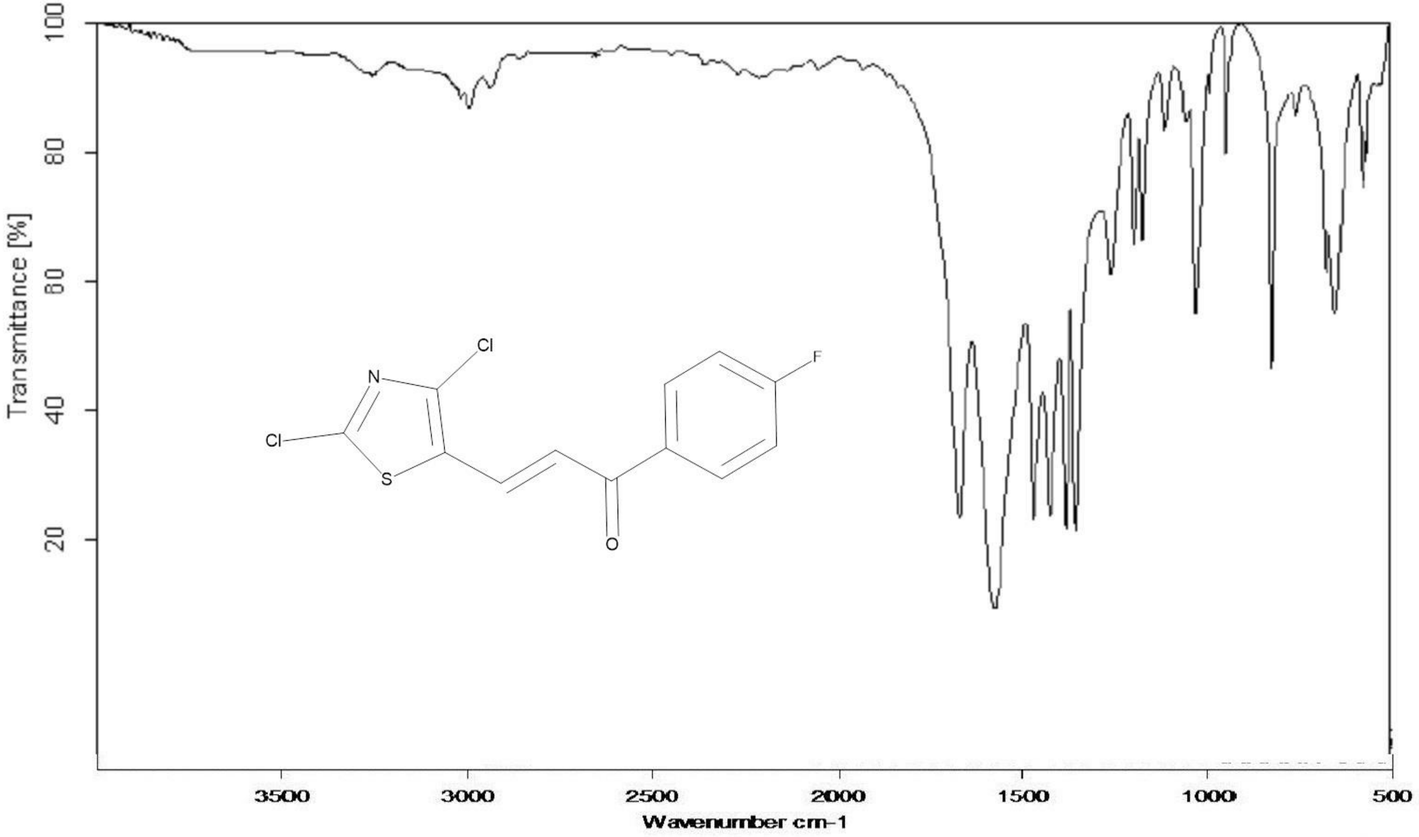

Figure S32. FT-IR Spectrum of compound 11

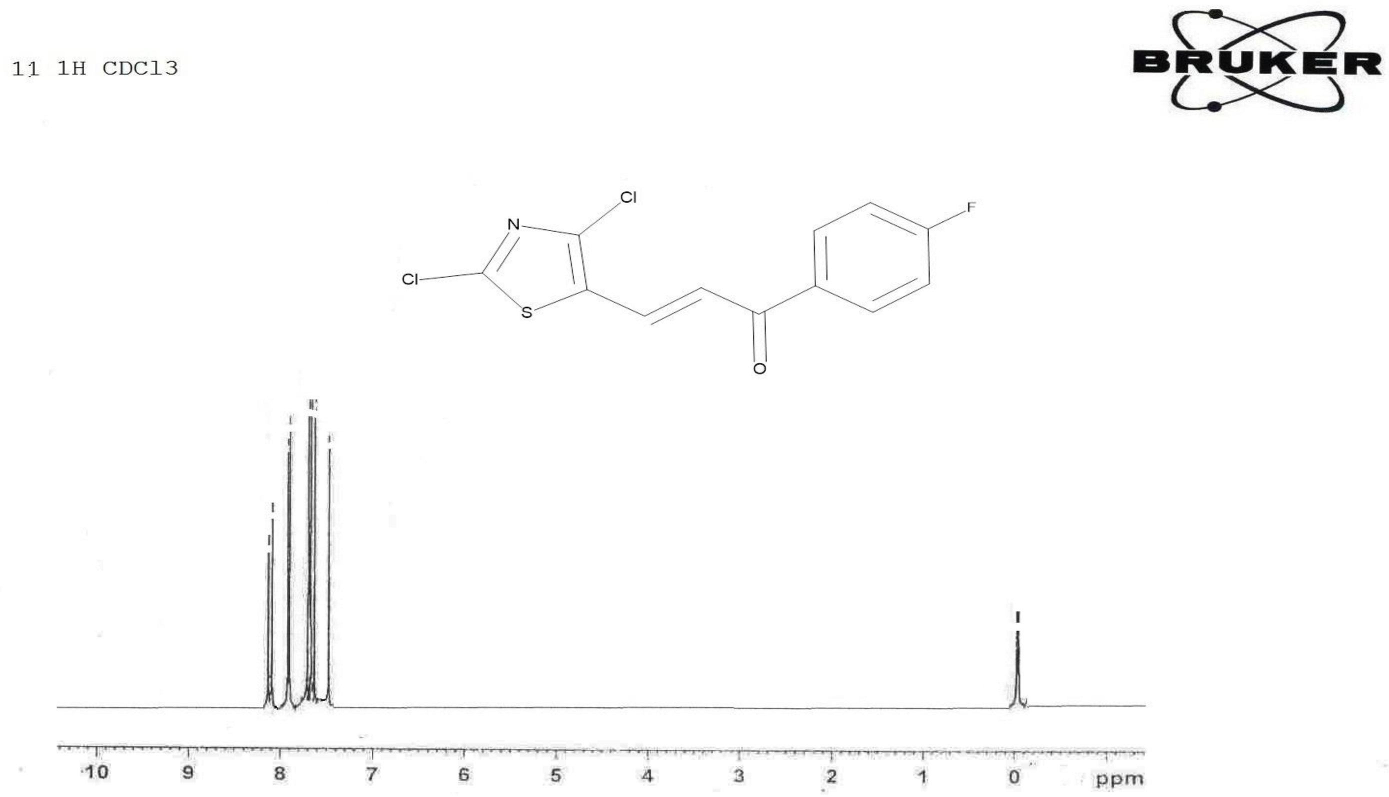

Figure S33. [1]**H NMR Spectrum of compound 11**

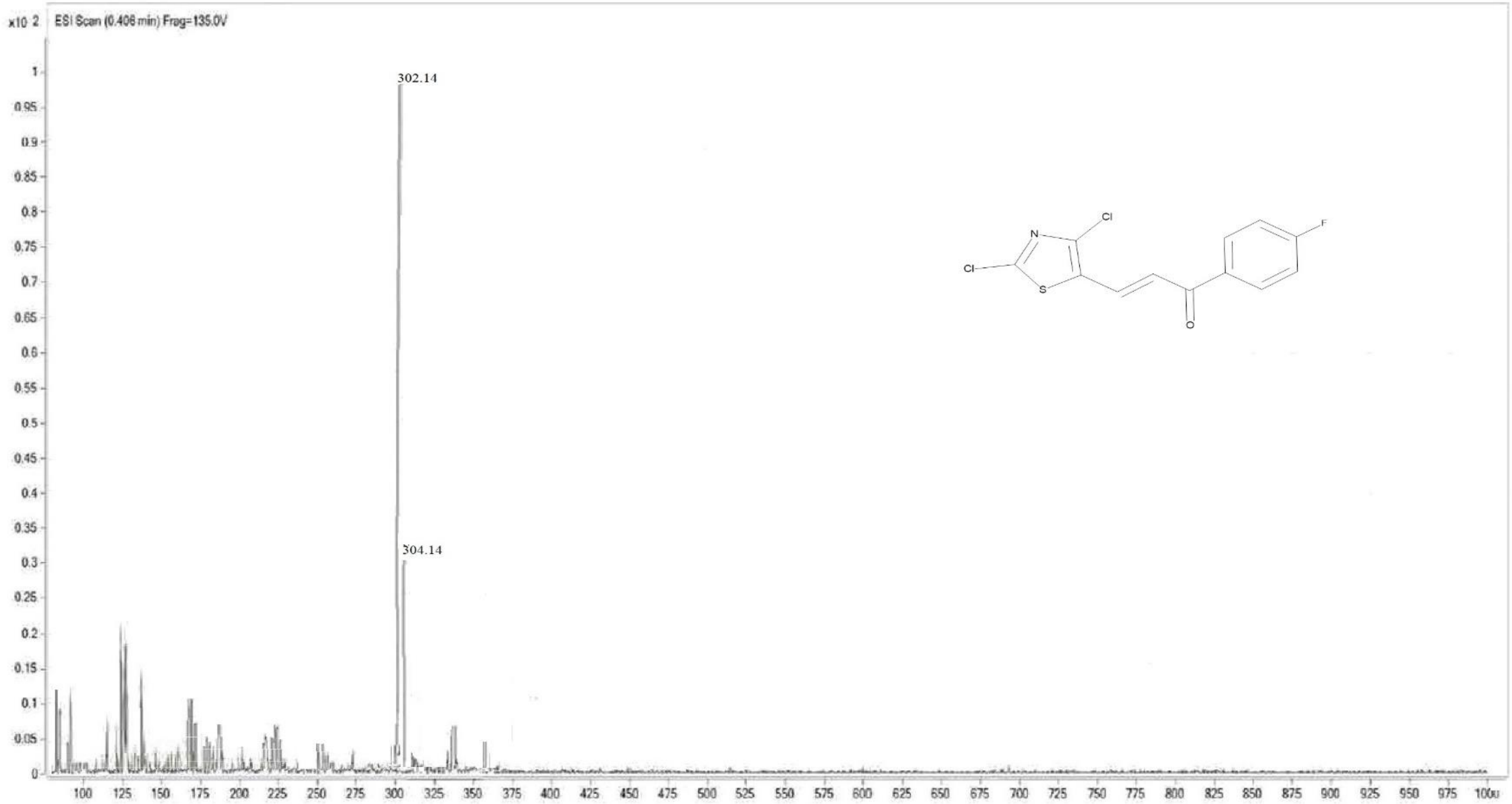

Figure S34. Mass Spectrum of compound 11

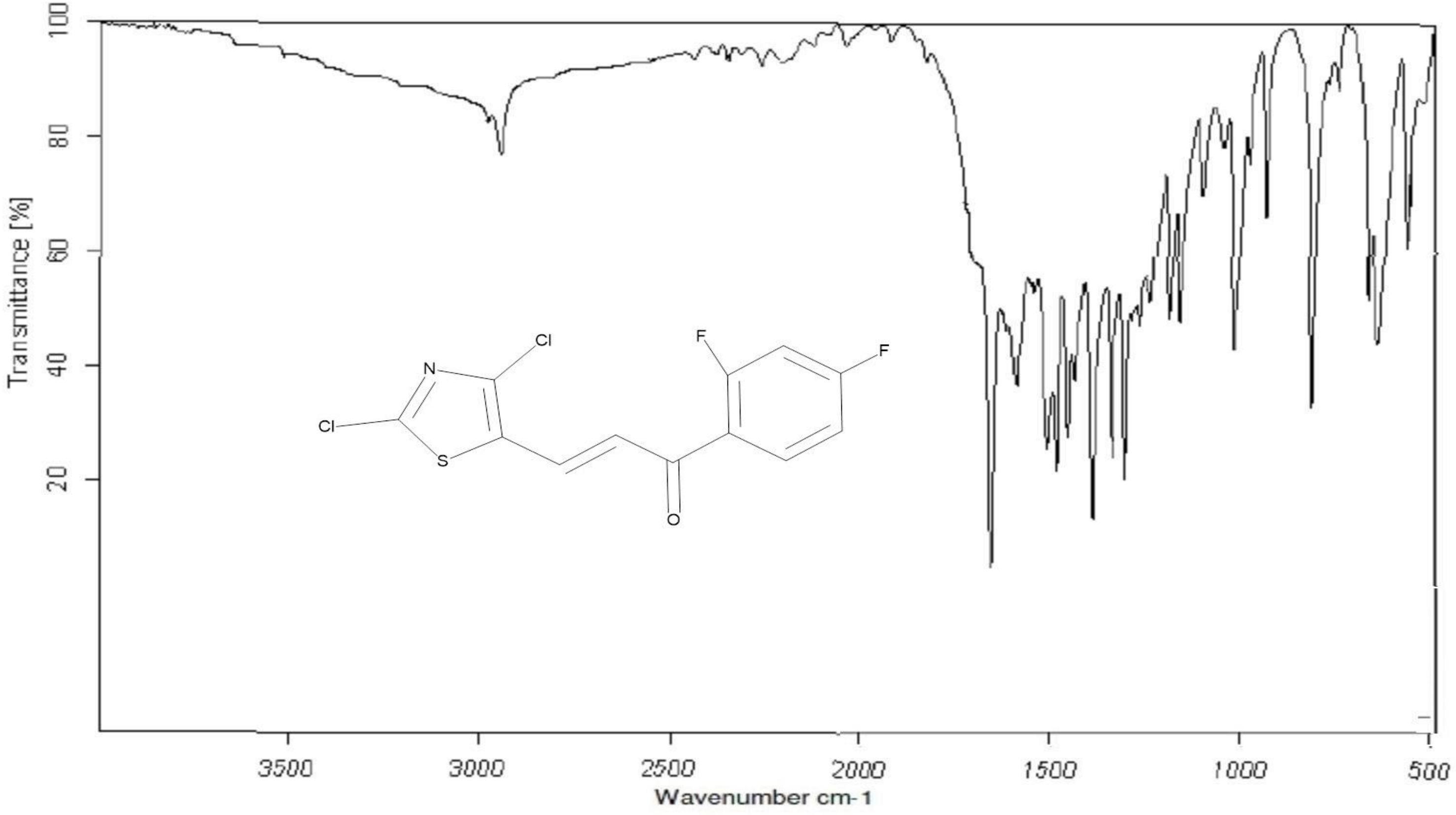

Figure S35. FT-IR Spectrum of compound 12

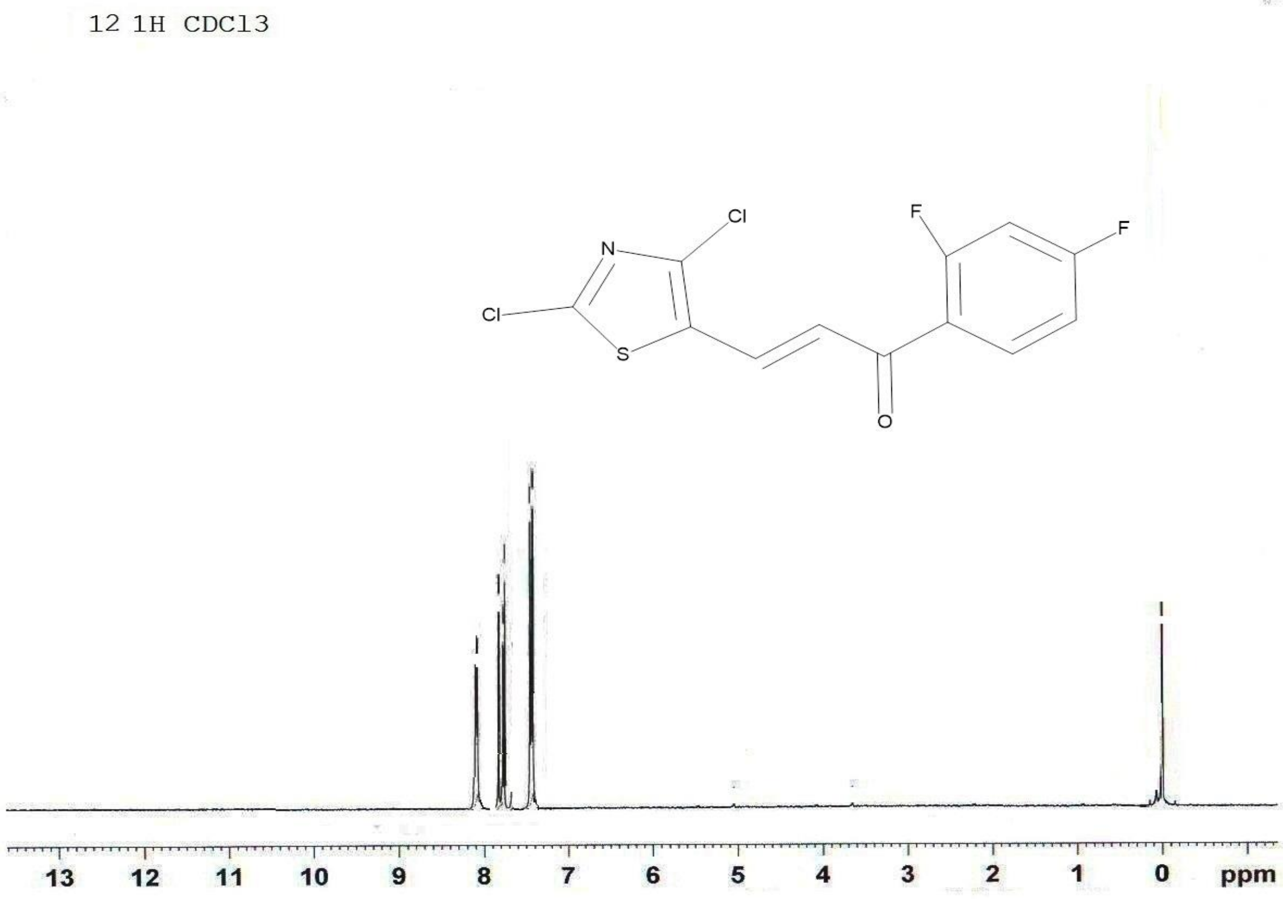

Figure S36. ¹H NMR Spectrum of compound 12

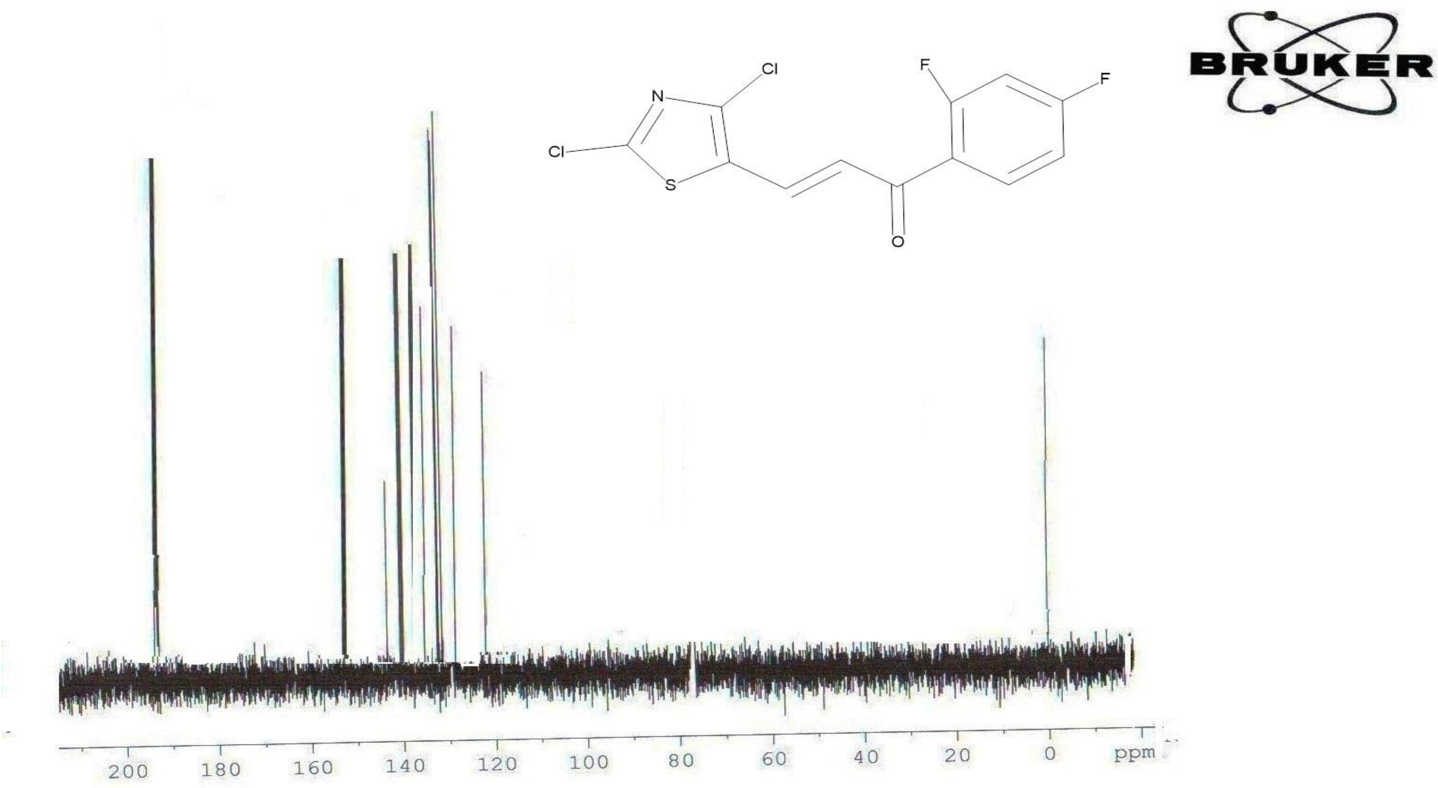

Figure S37. ^{13}C NMR Spectrum of compound 12

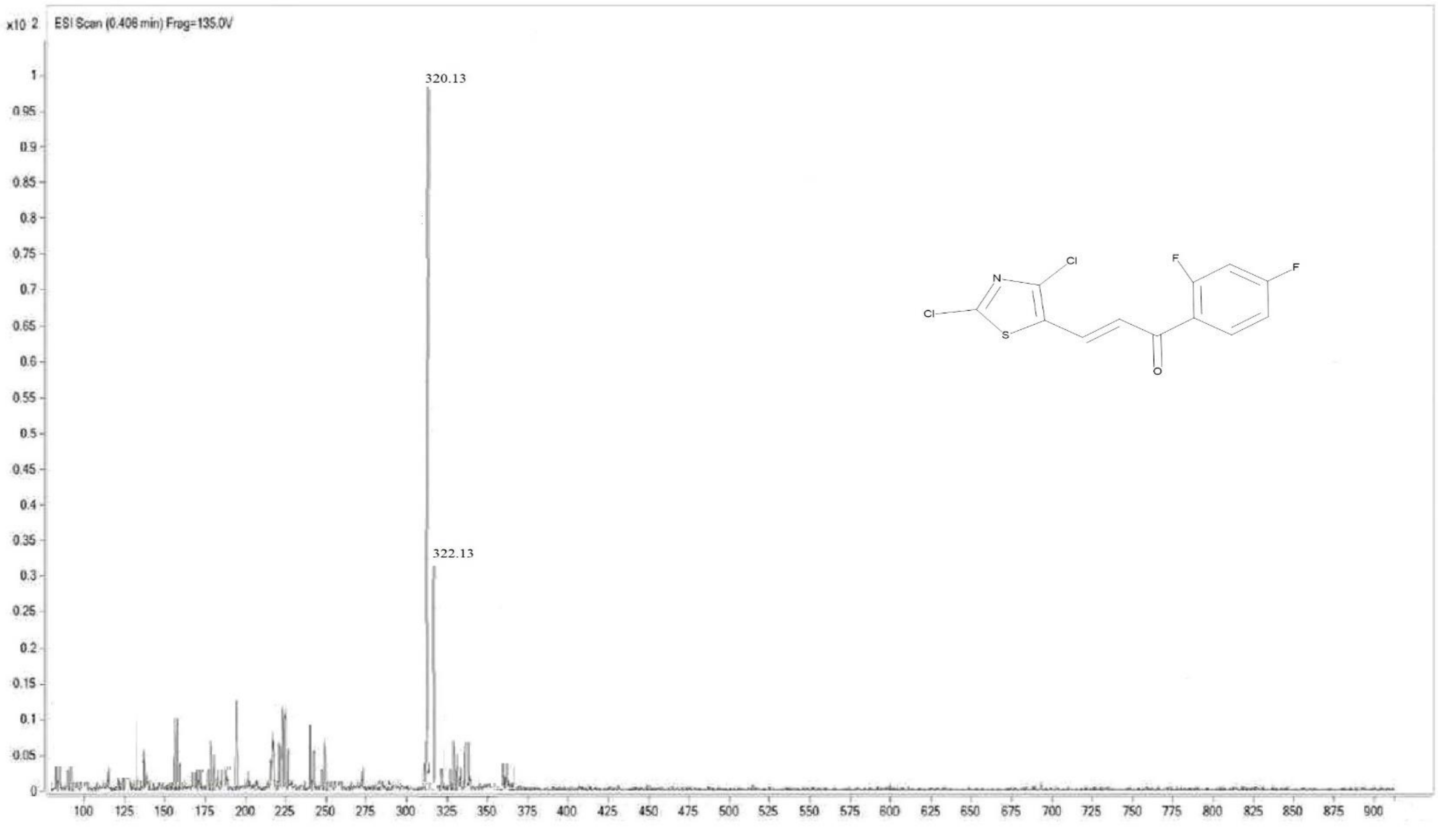

Figure S38. Mass Spectrum of compound 12

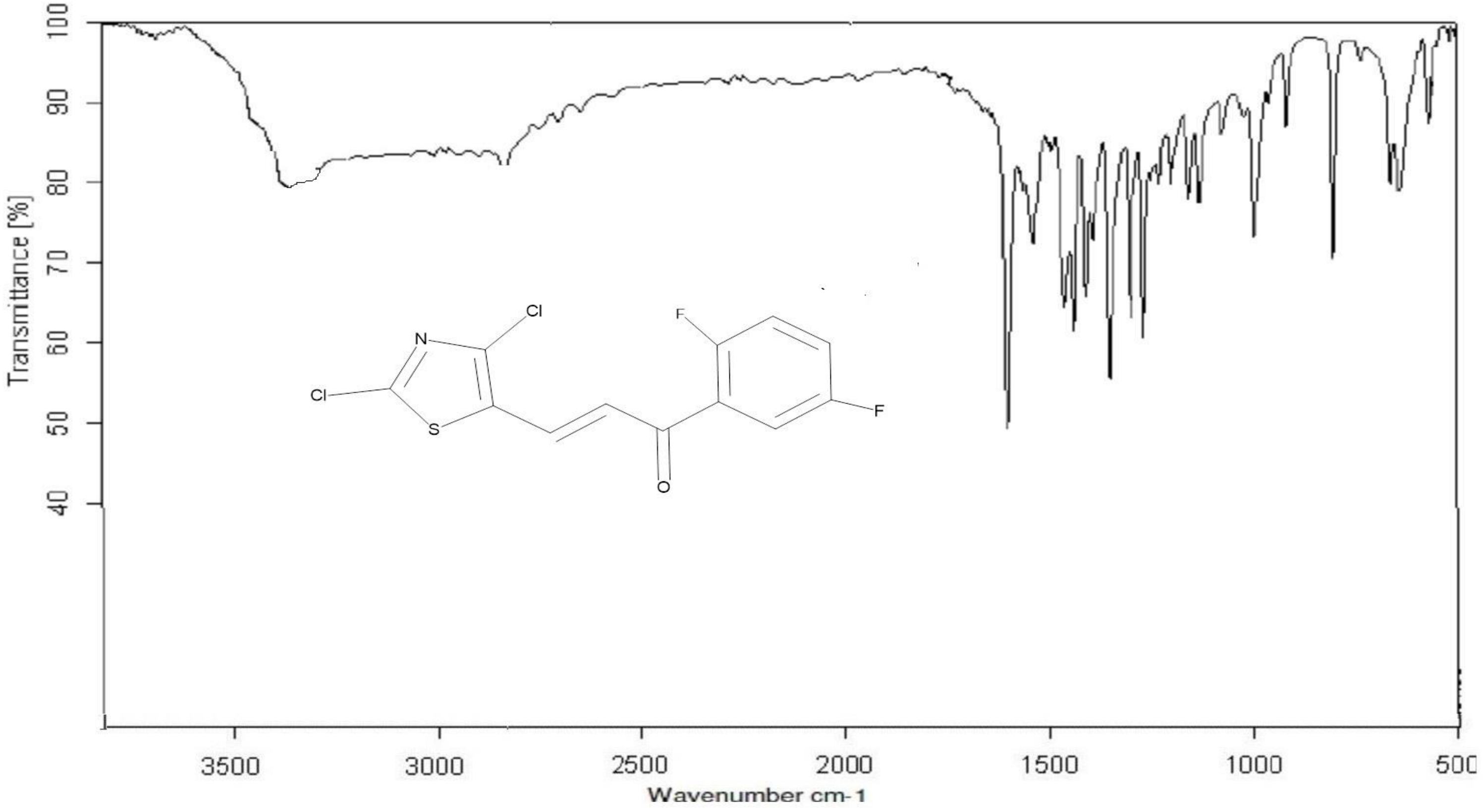

Figure S39. FT-IR Spectrum of compound 13

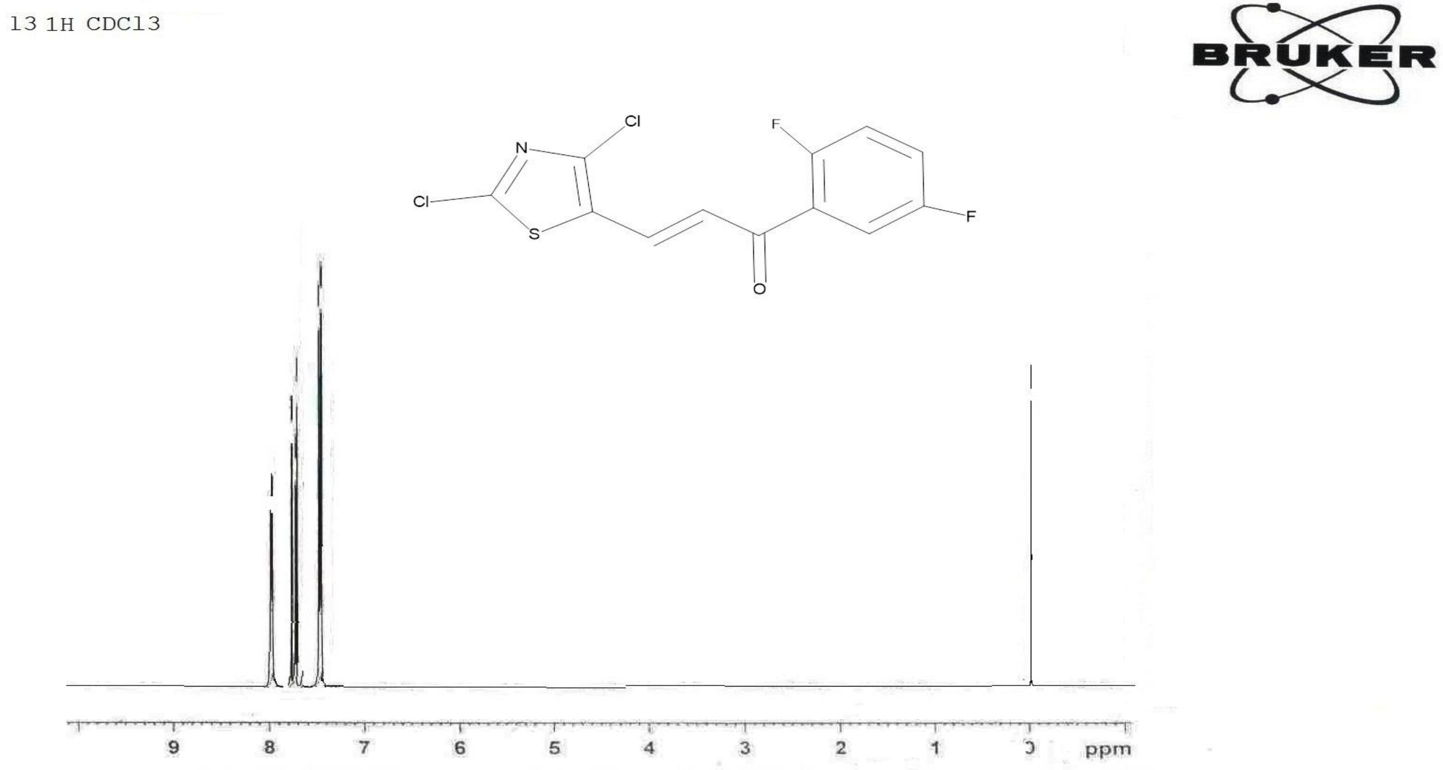

Figure S40. ^{1}H NMR Spectrum of compound 13

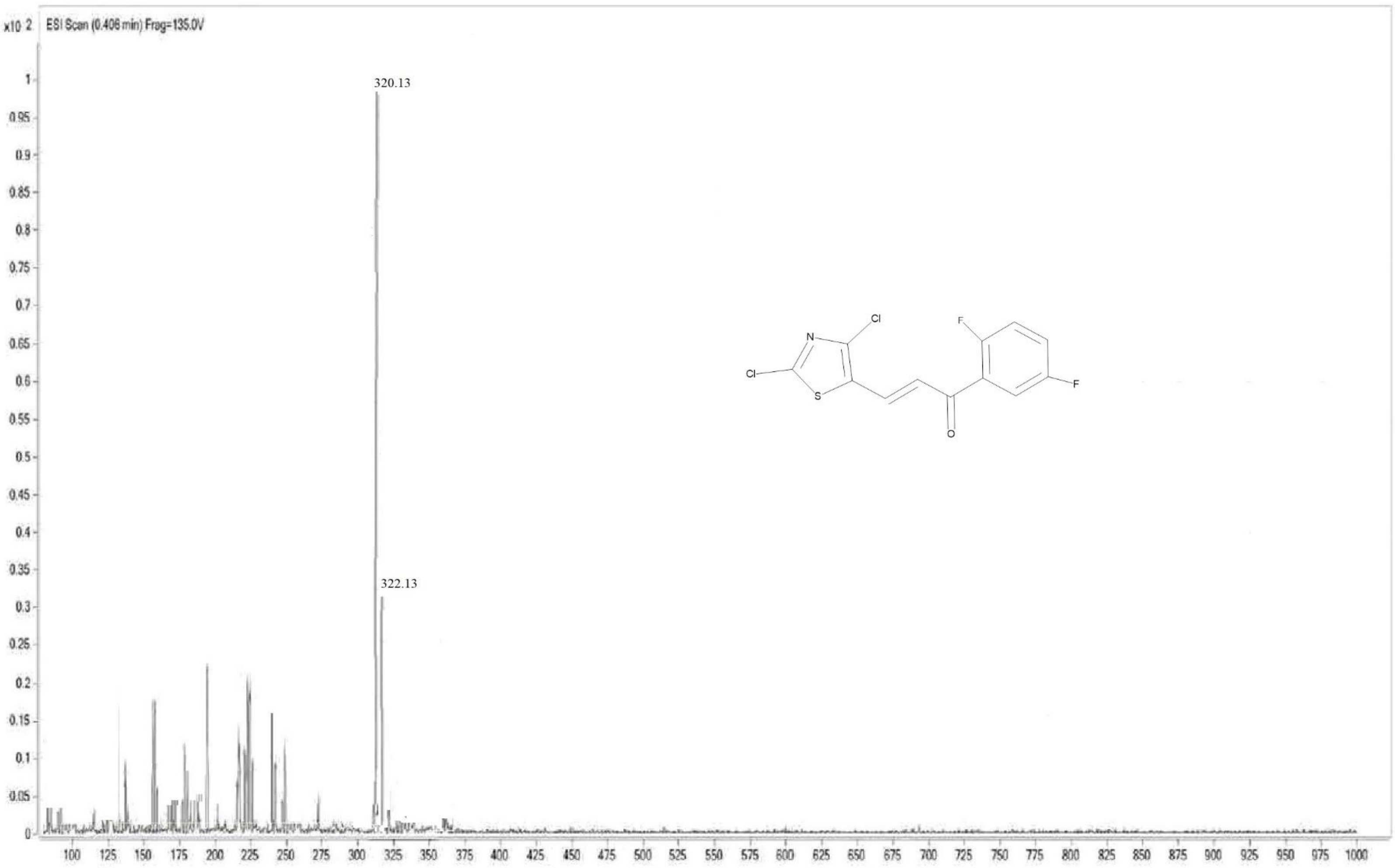

Figure S41. Mass Spectrum of compound 13

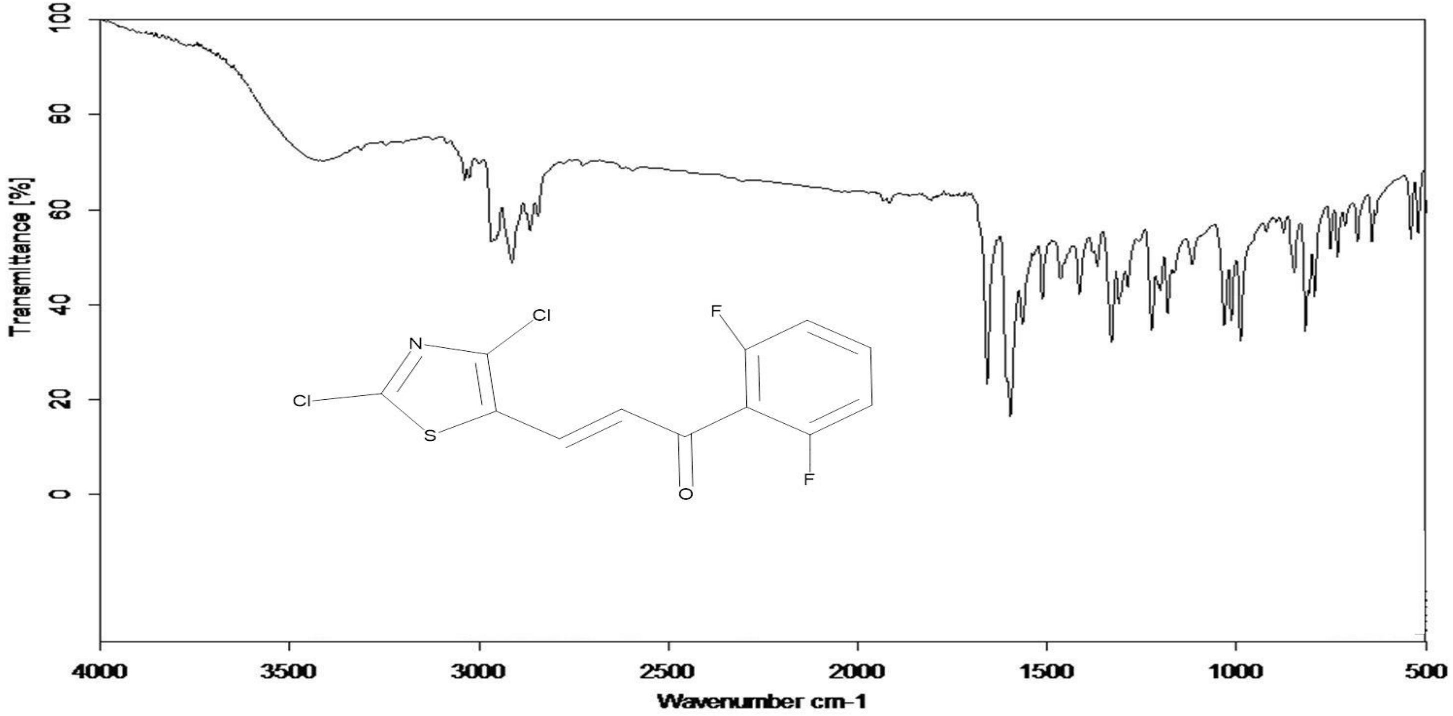

Figure S42. FT-IR Spectrum of compound 14

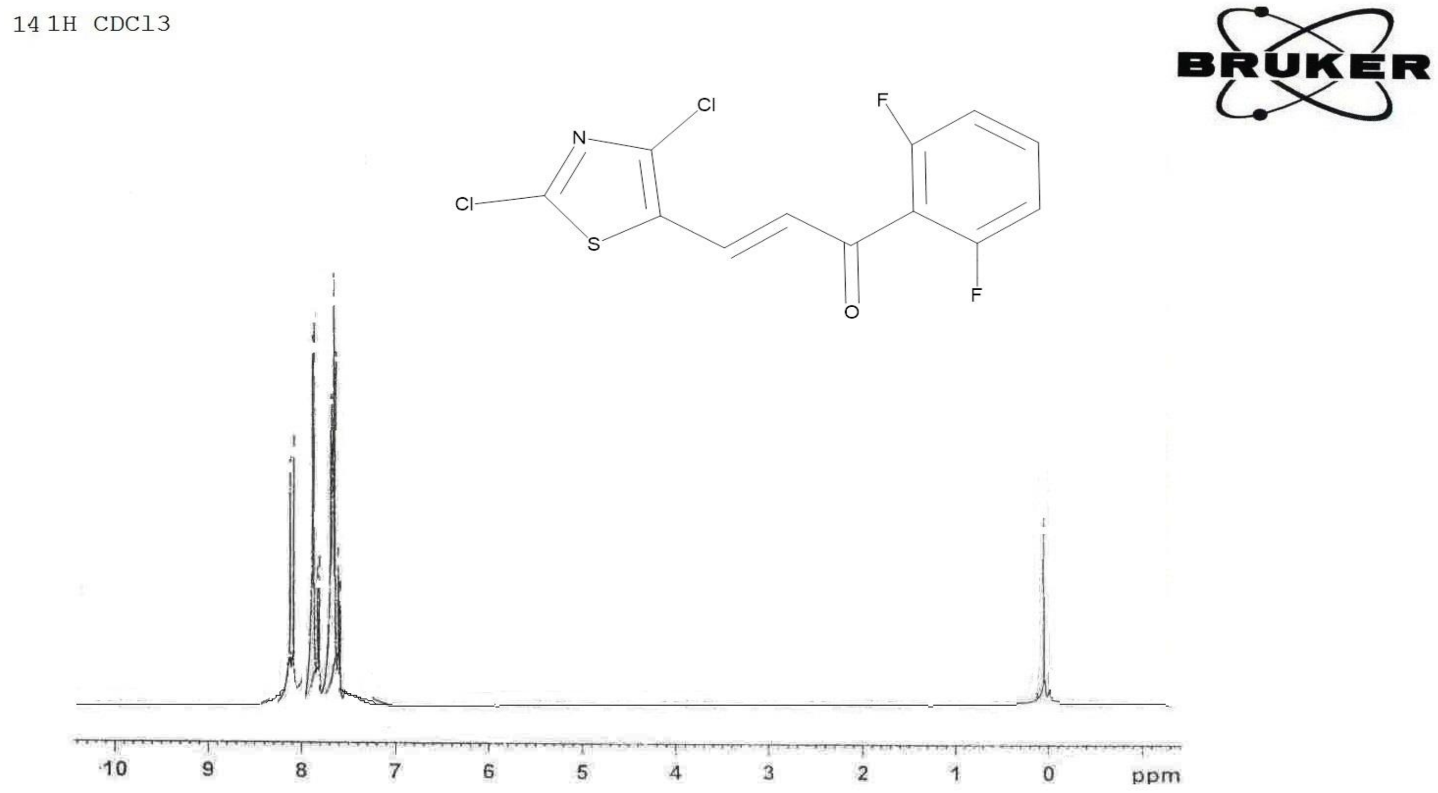

Figure S43. ¹H NMR Spectrum of compound 14

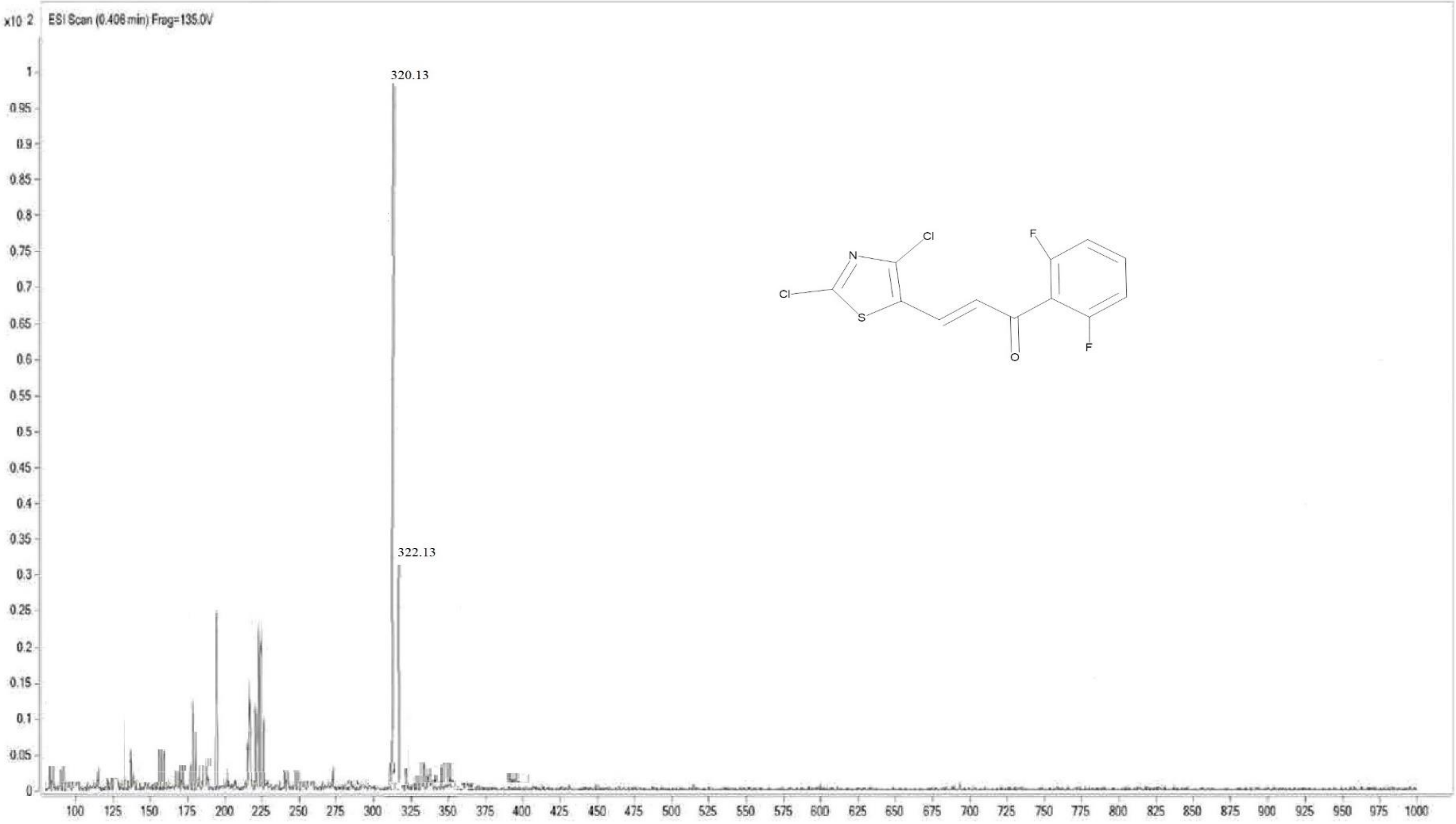

Figure S44. Mass Spectrum of compound 14

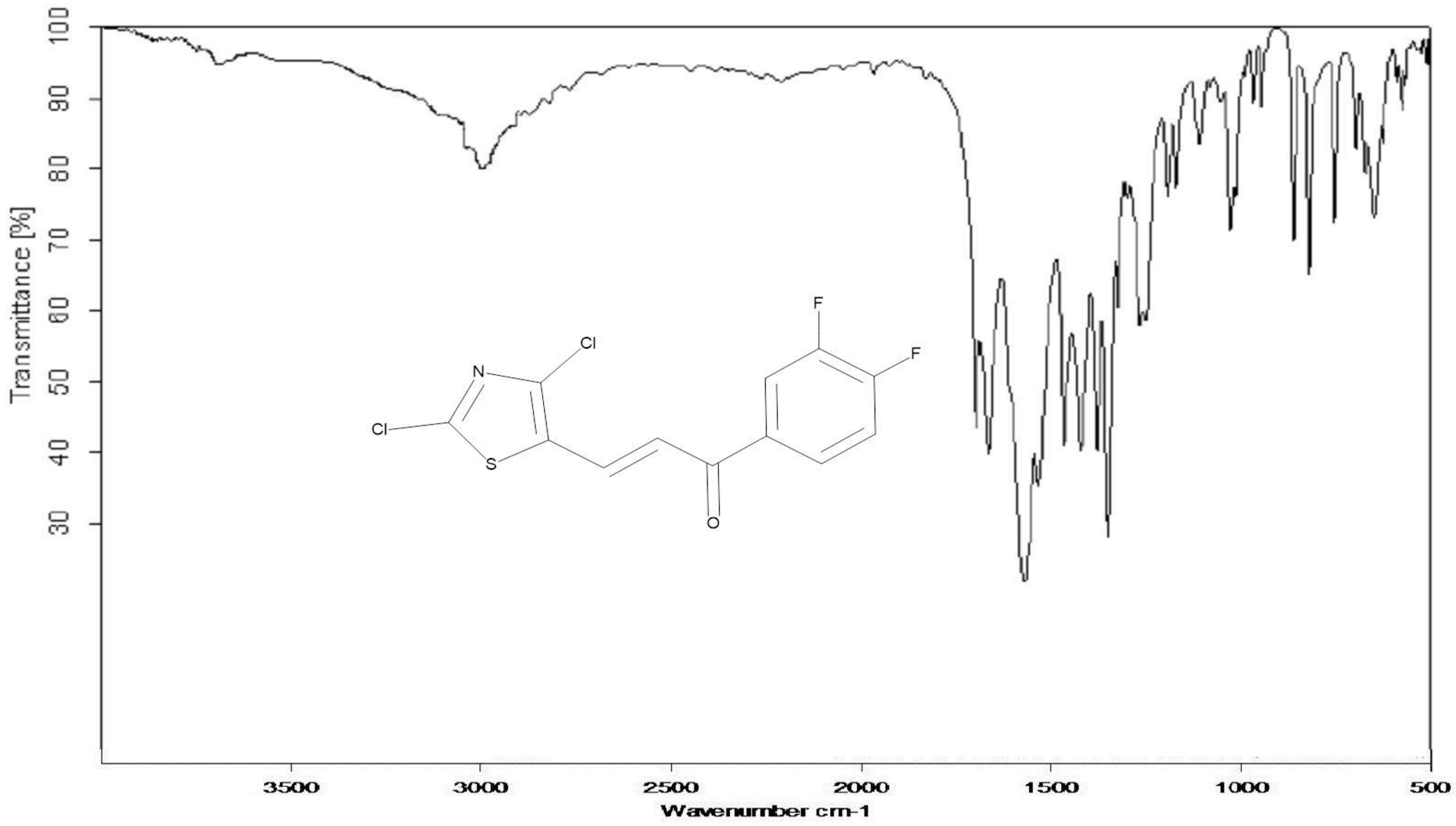

Figure S45. FT-IR Spectrum of compound 15

15 1H CDCl3

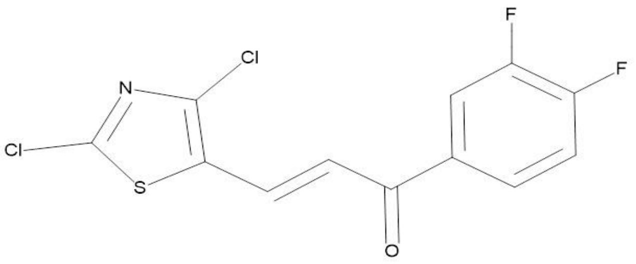

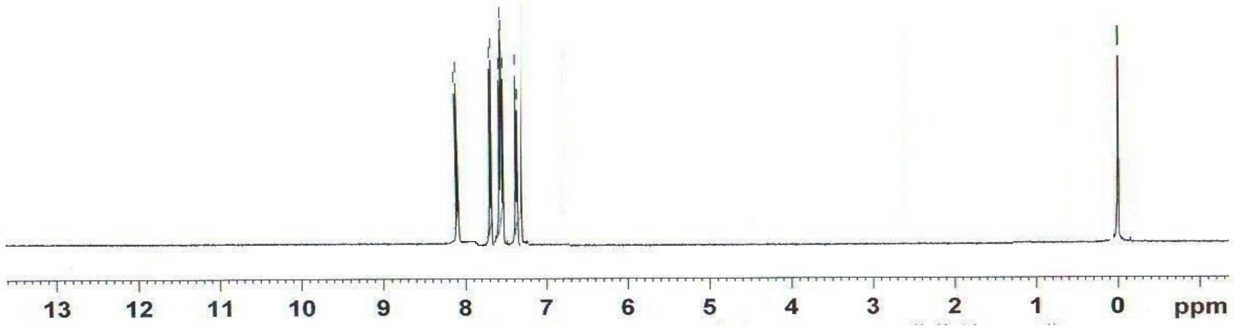

Figure S46. 1H NMR Spectrum of compound 15

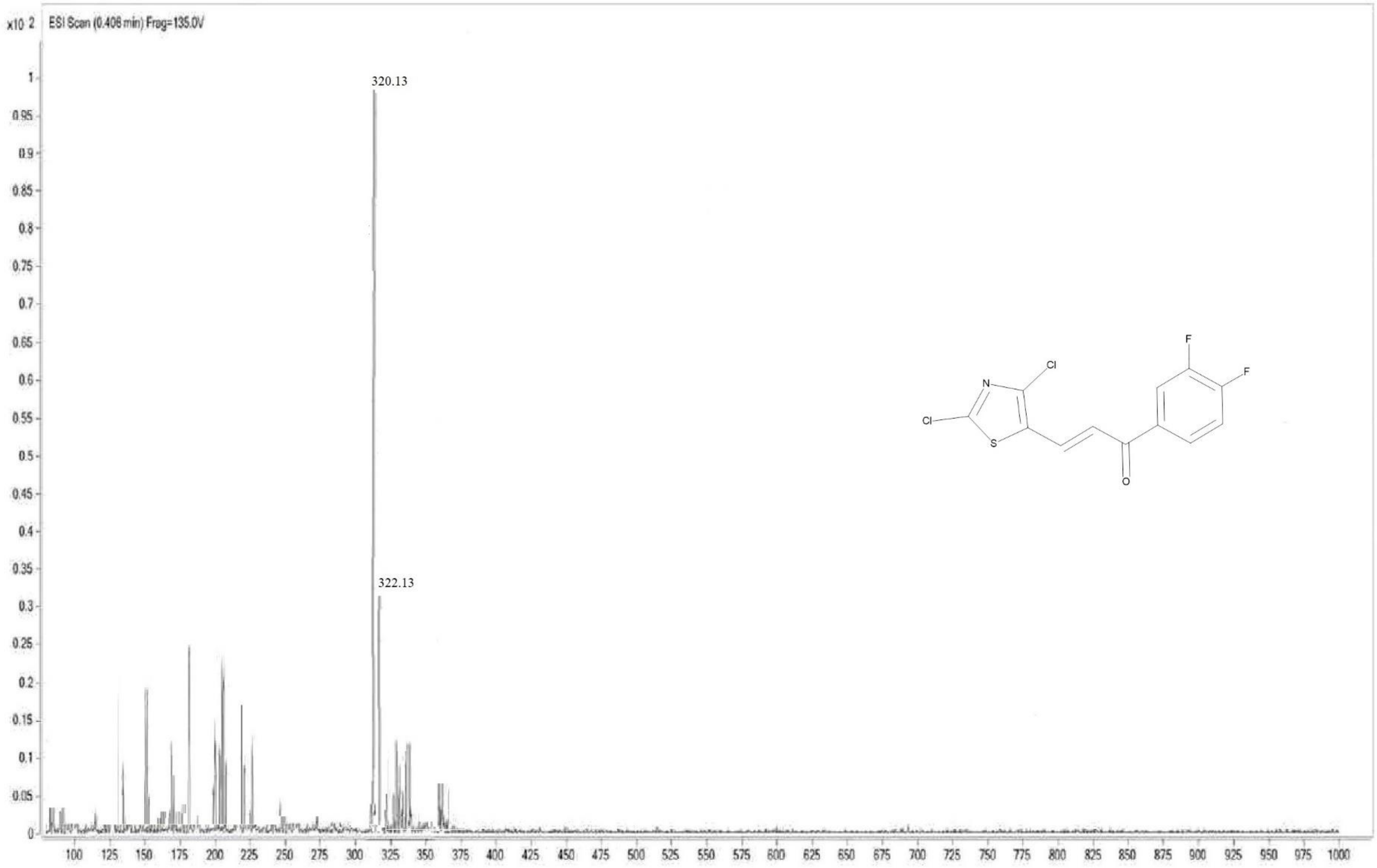

Figure S47. Mass Spectrum of compound 15

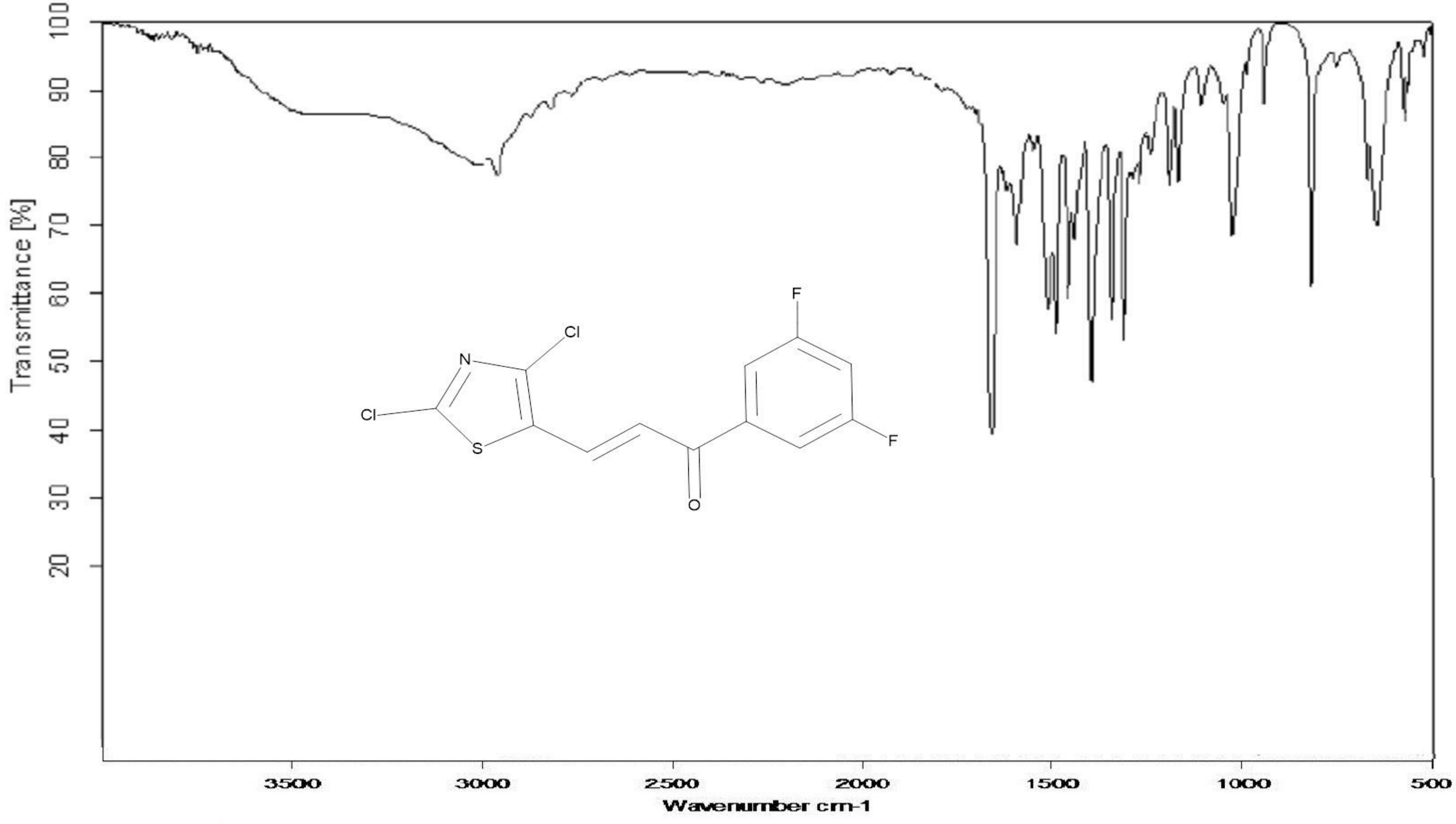

Figure S48. FT-IR Spectrum of compound 16

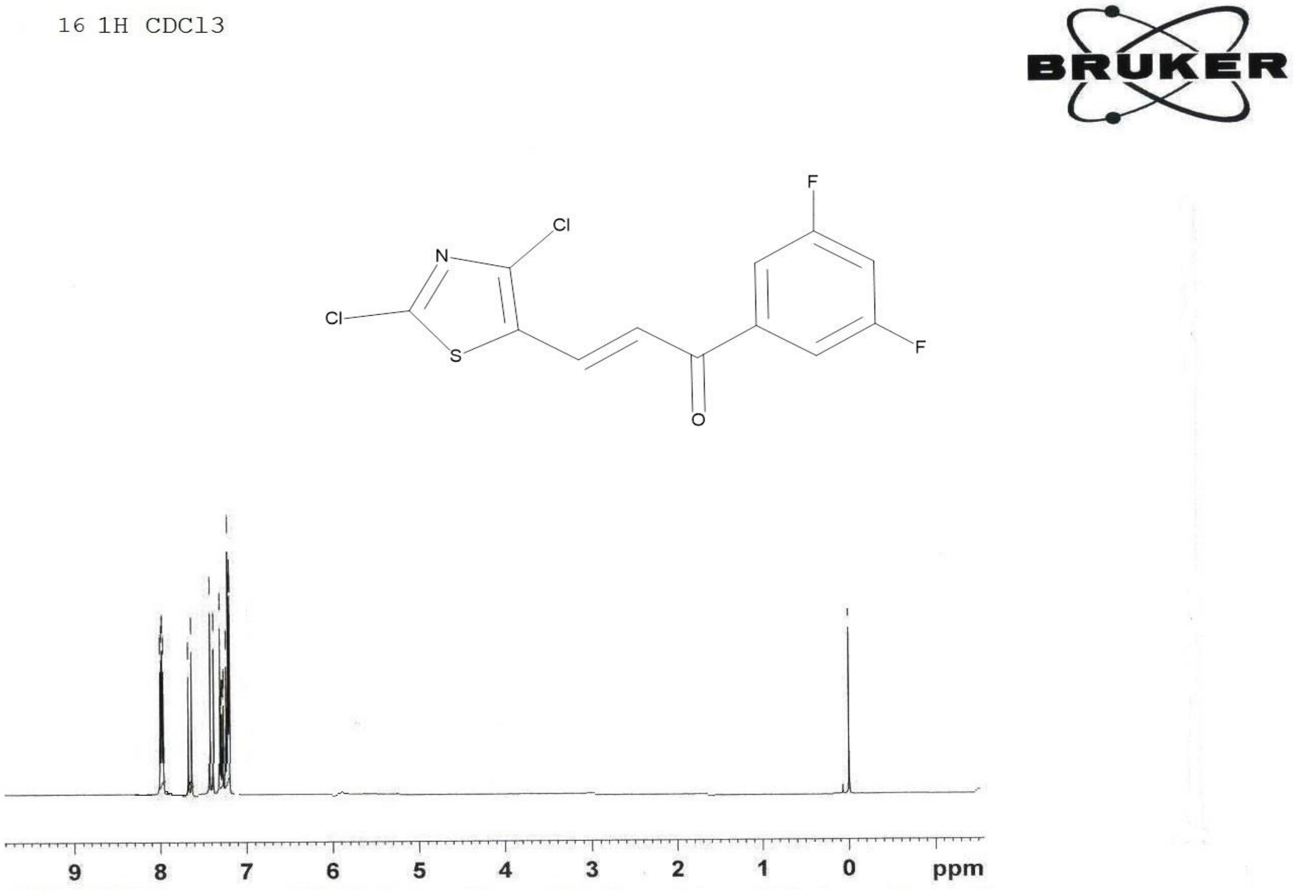

Figure S49. ^{1}H **NMR Spectrum of compound 16**

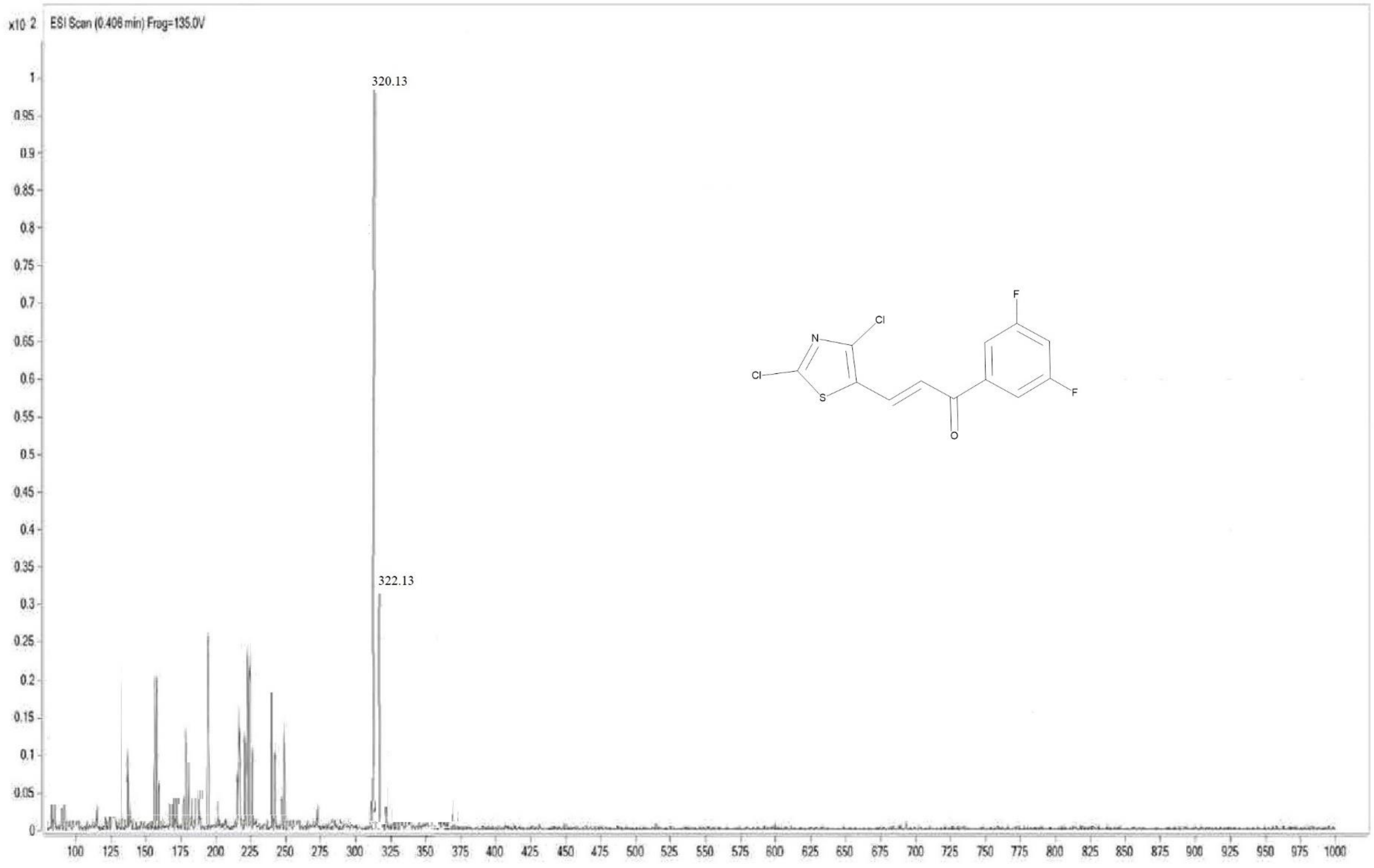

Figure S50. Mass Spectrum of compound 16

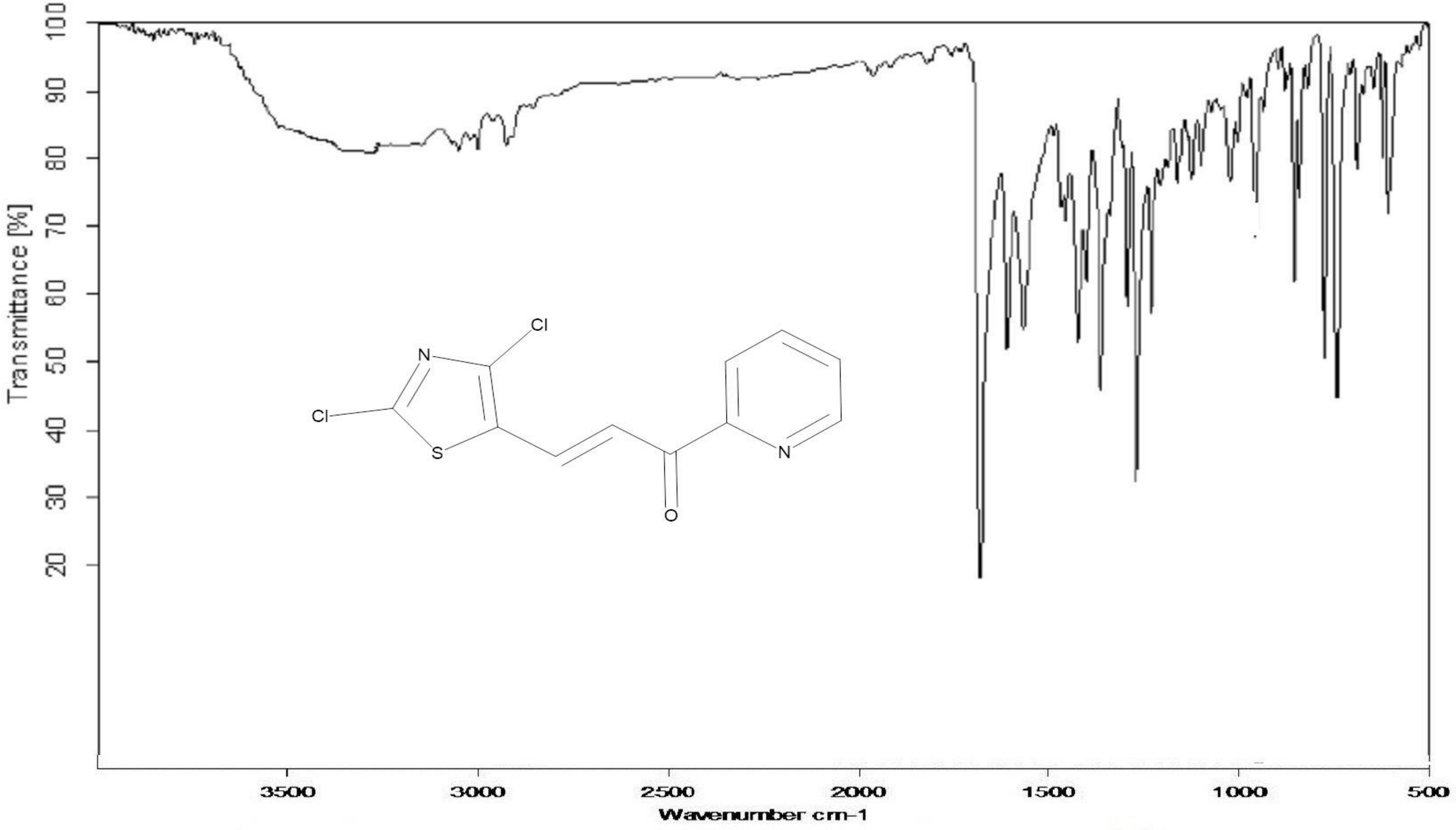

Figure S51. FT-IR Spectrum of compound 17

17 1H CDCl3

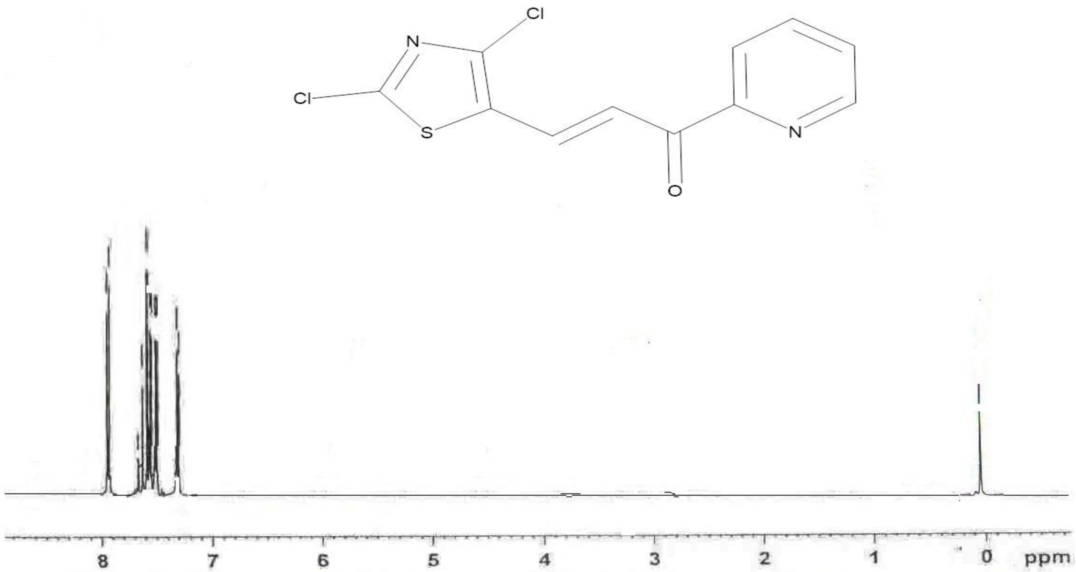

Figure S52. ¹H NMR Spectrum of compound 17

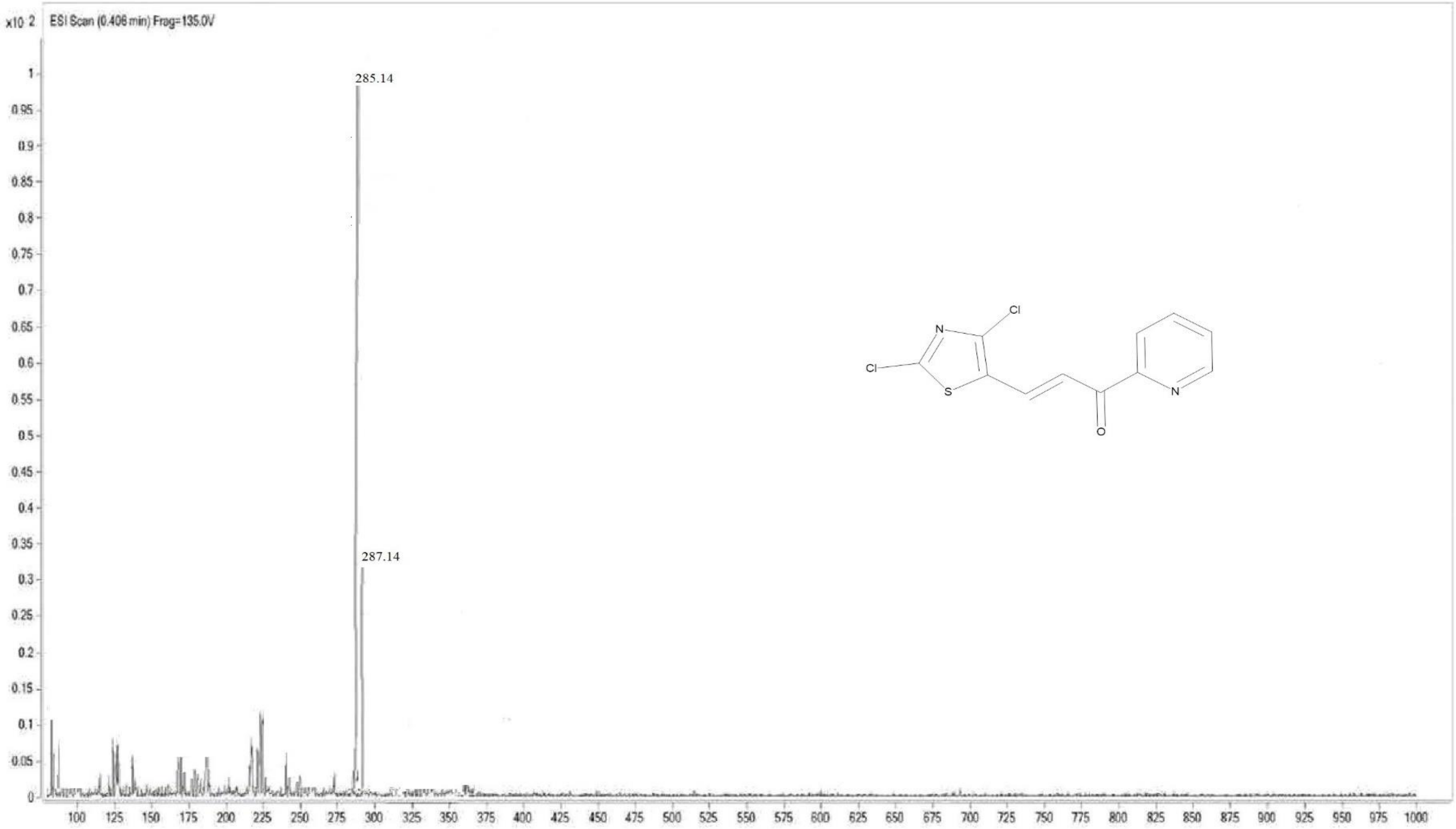

Figure S53. Mass Spectrum of compound 17

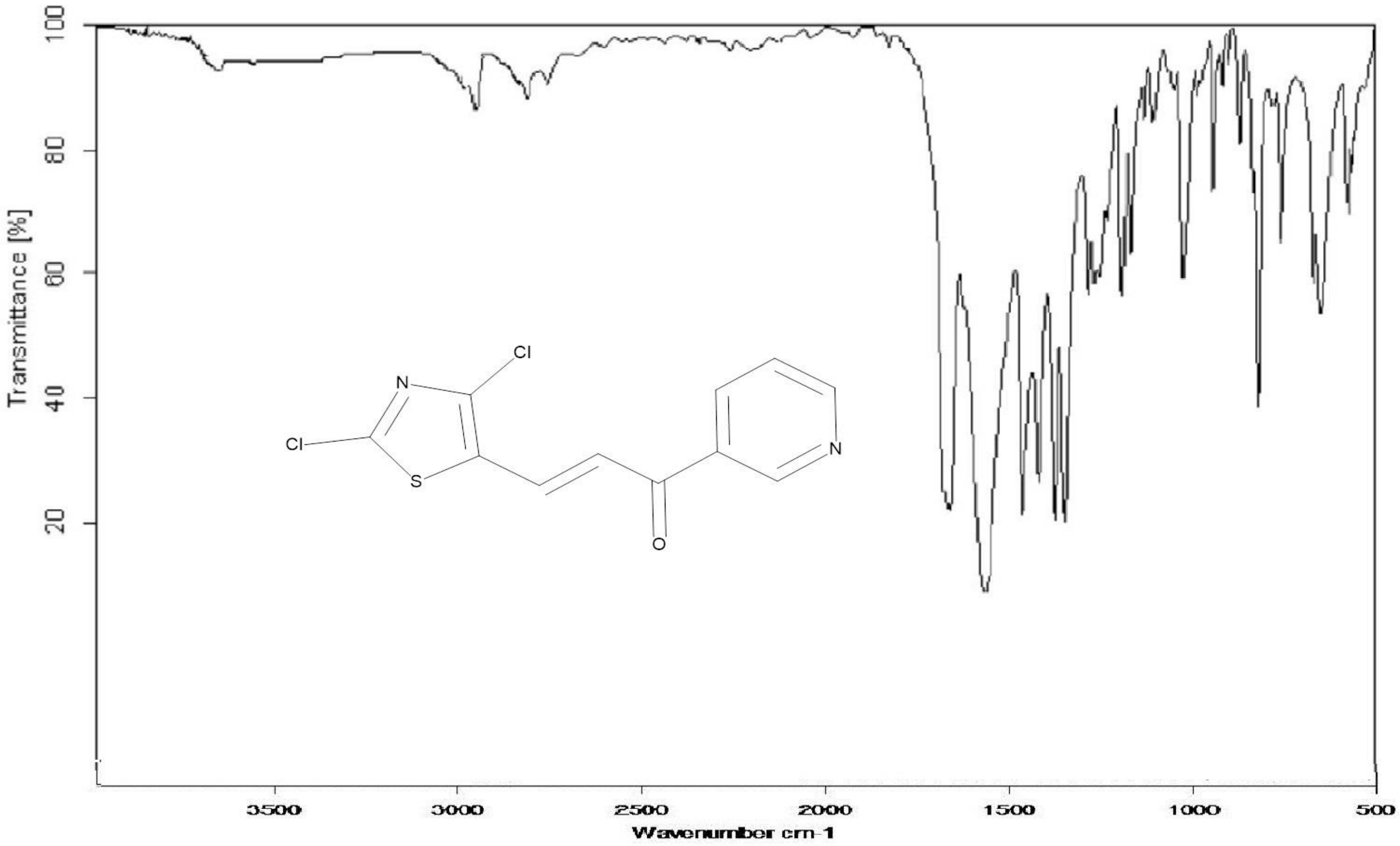

Figure S54. FT-IR Spectrum of compound 18

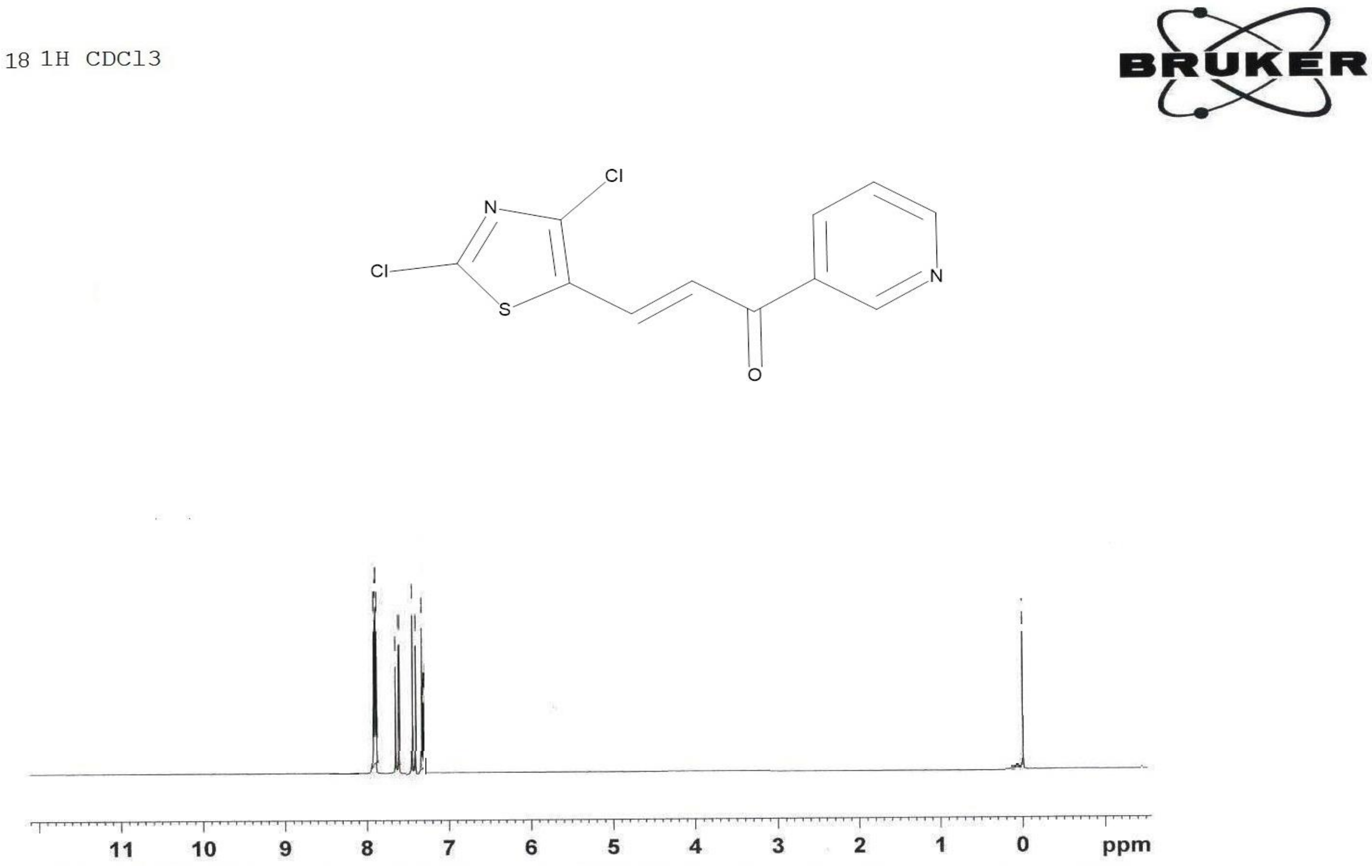

Figure S55. ^{1}H NMR Spectrum of compound 18

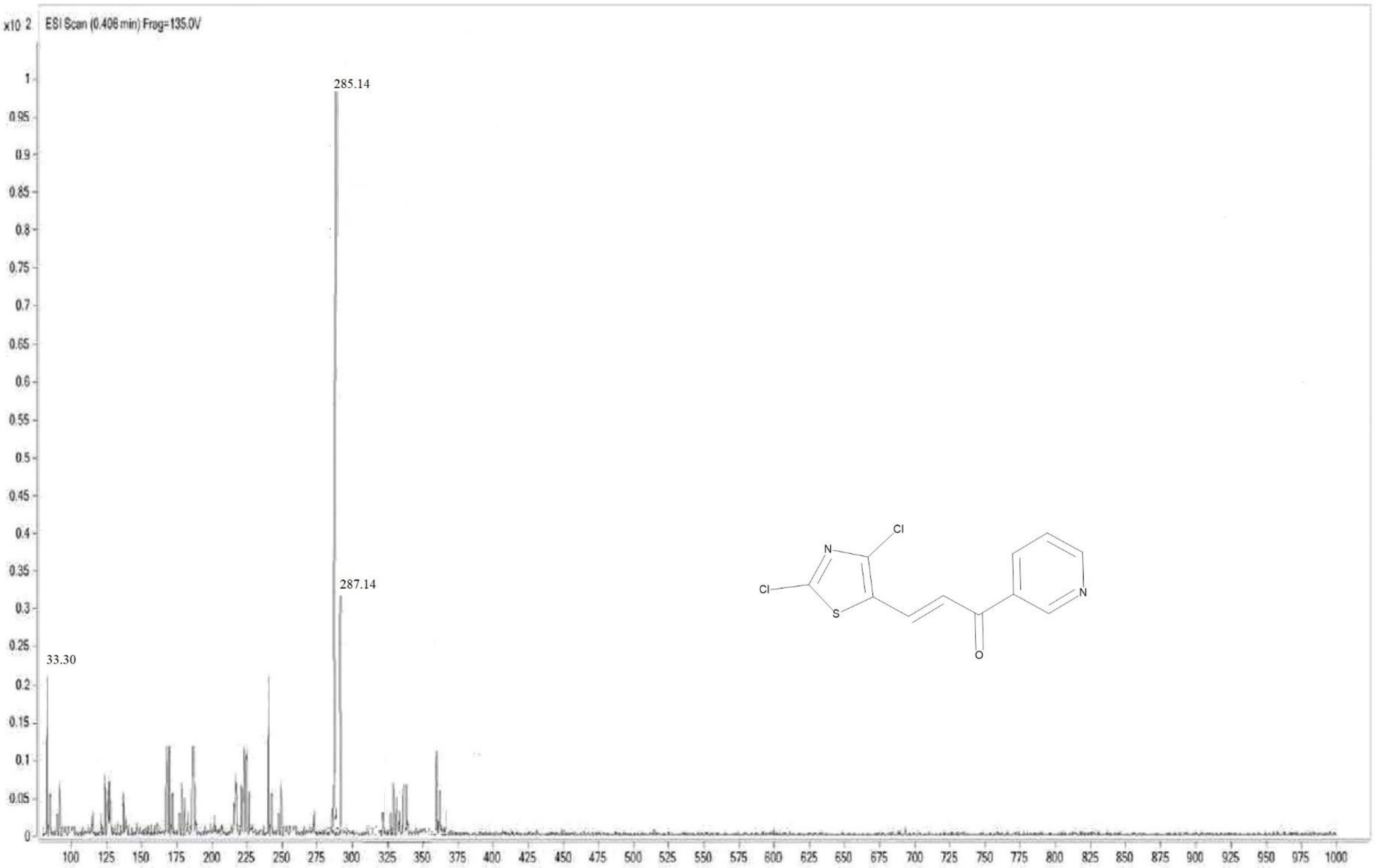

Figure S56. Mass Spectrum of compound 18

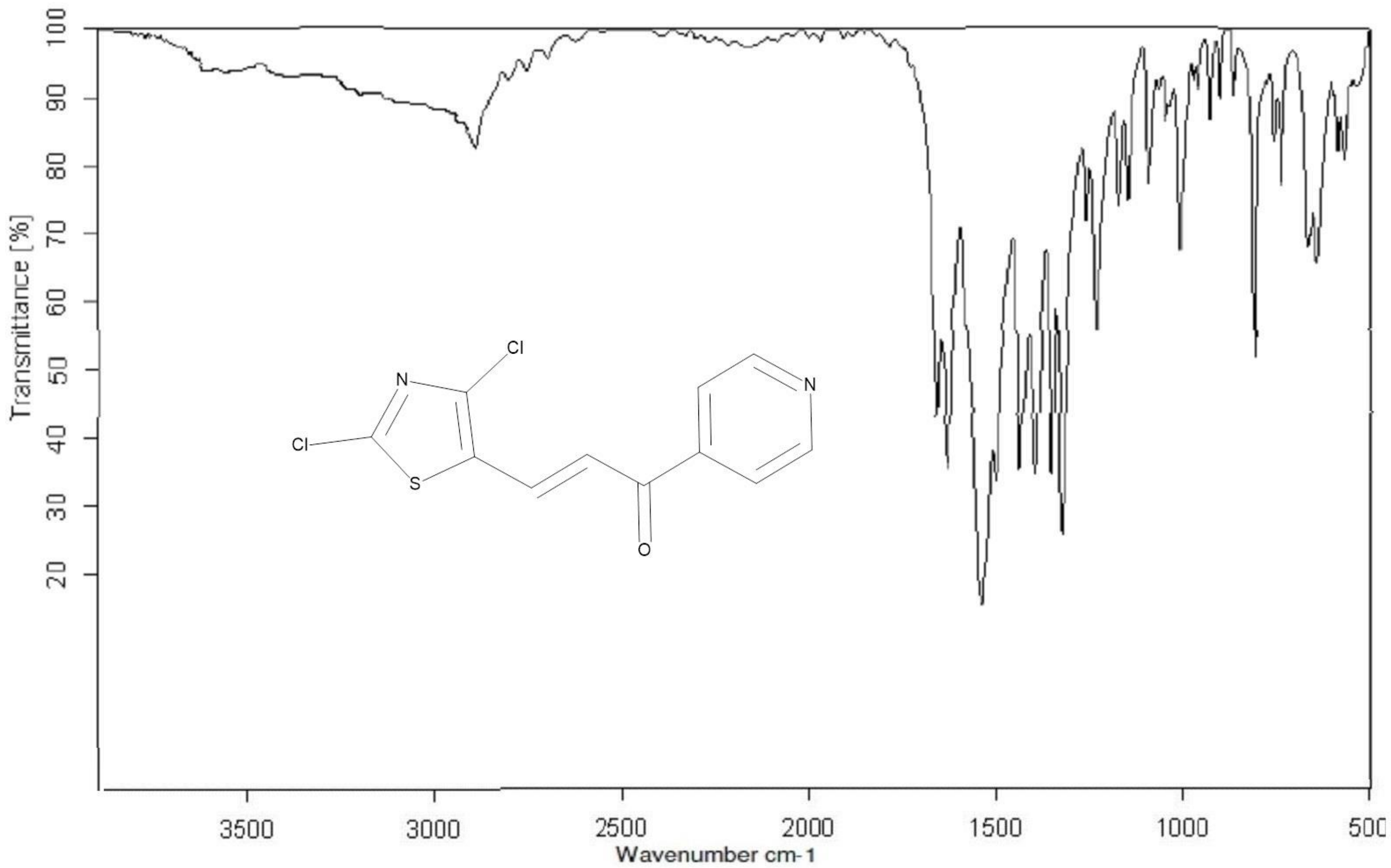

Figure S57. FT-IR Spectrum of compound 19

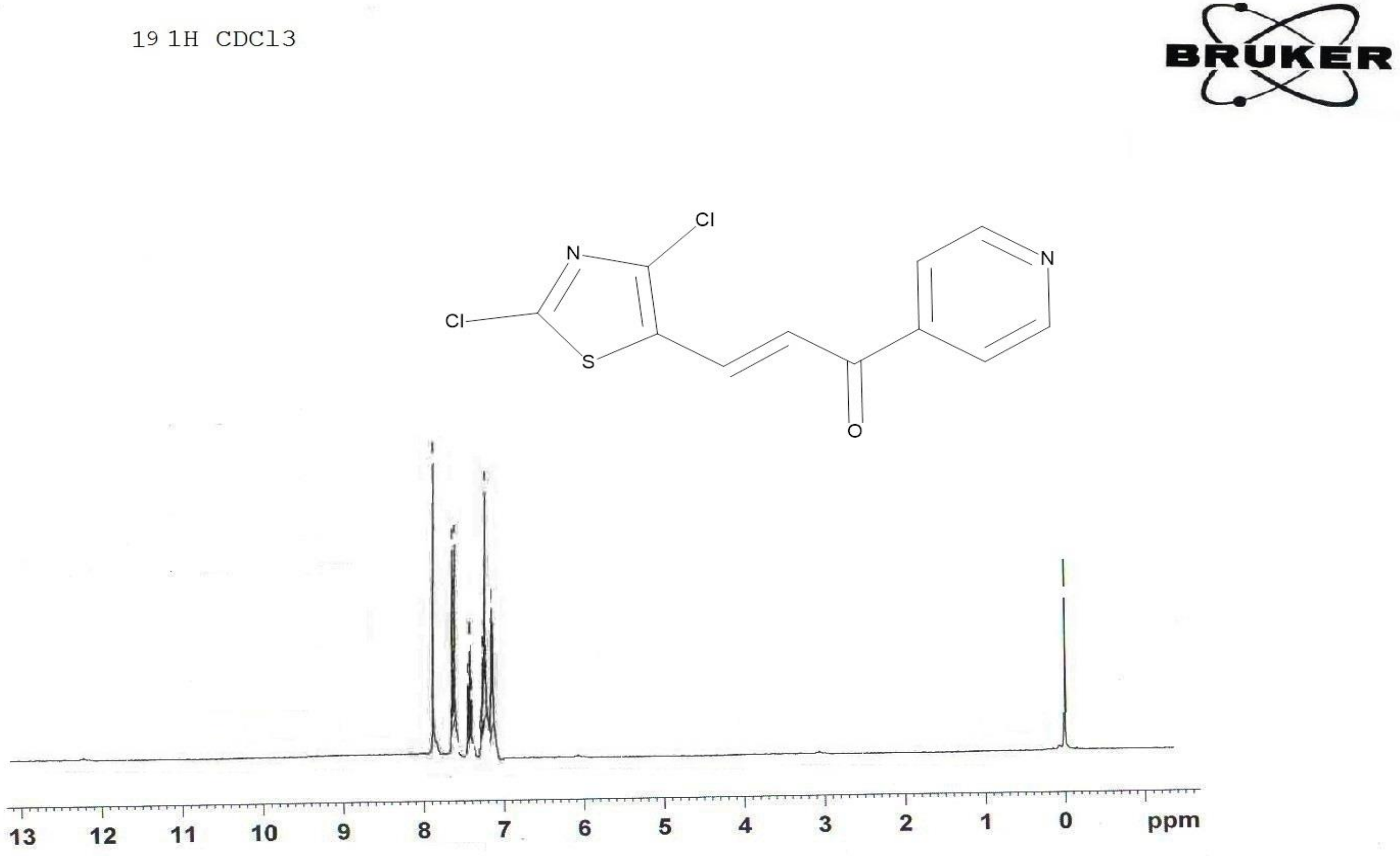

Figure S58. ^{1}H NMR Spectrum of compound 19

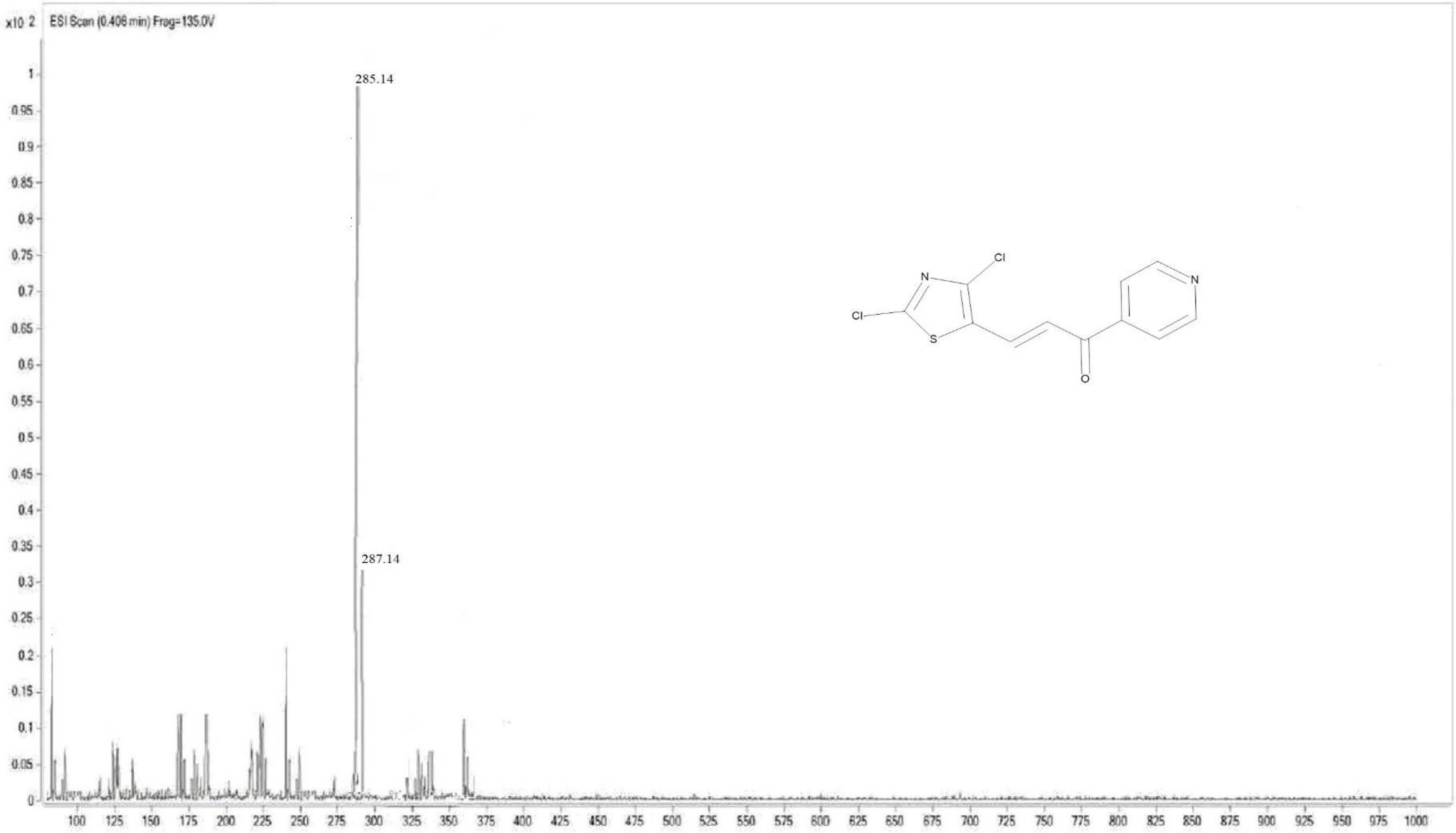

Figure S59. Mass Spectrum of compound 19

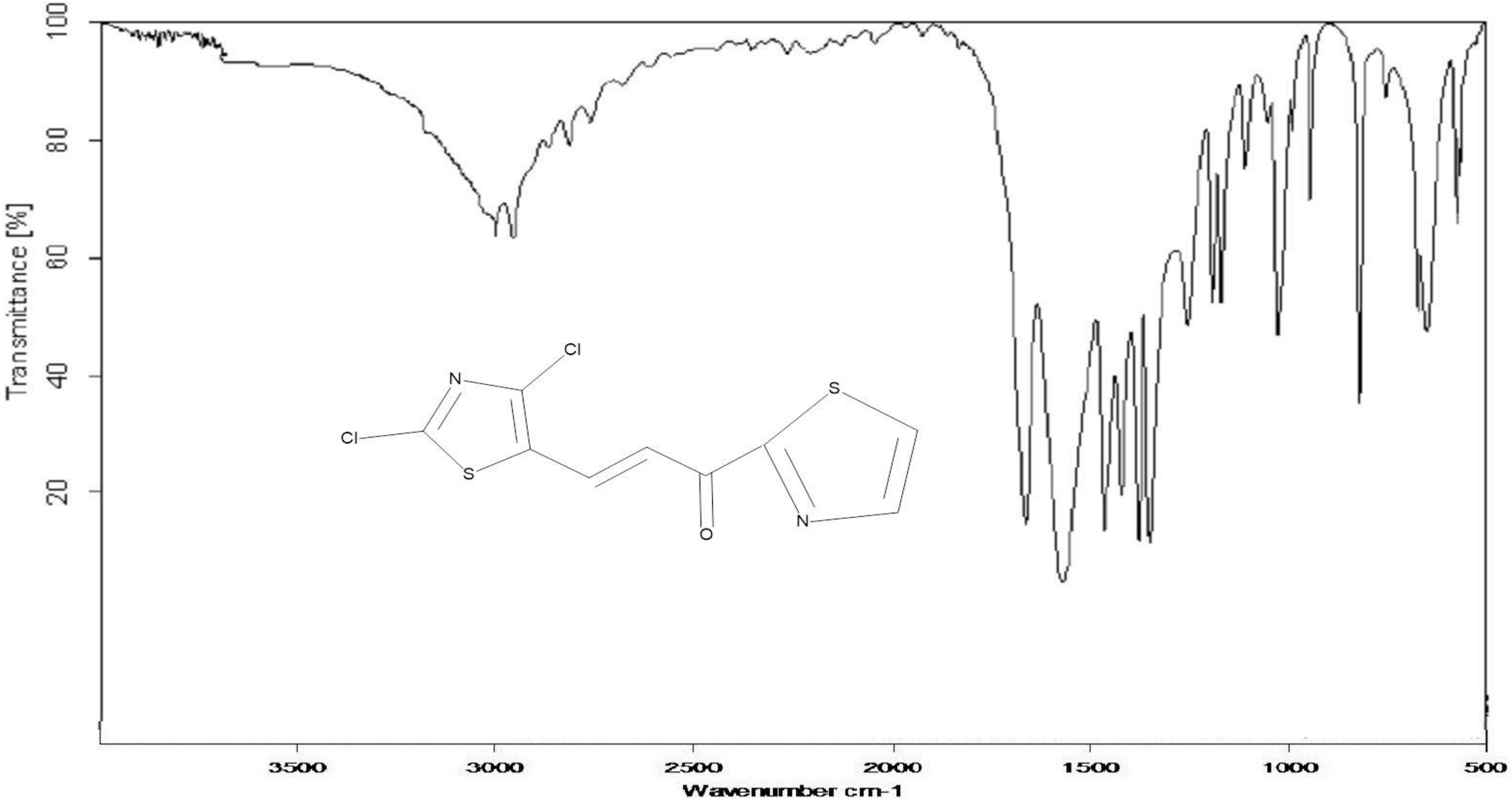

Figure S60. FT-IR Spectrum of compound 20

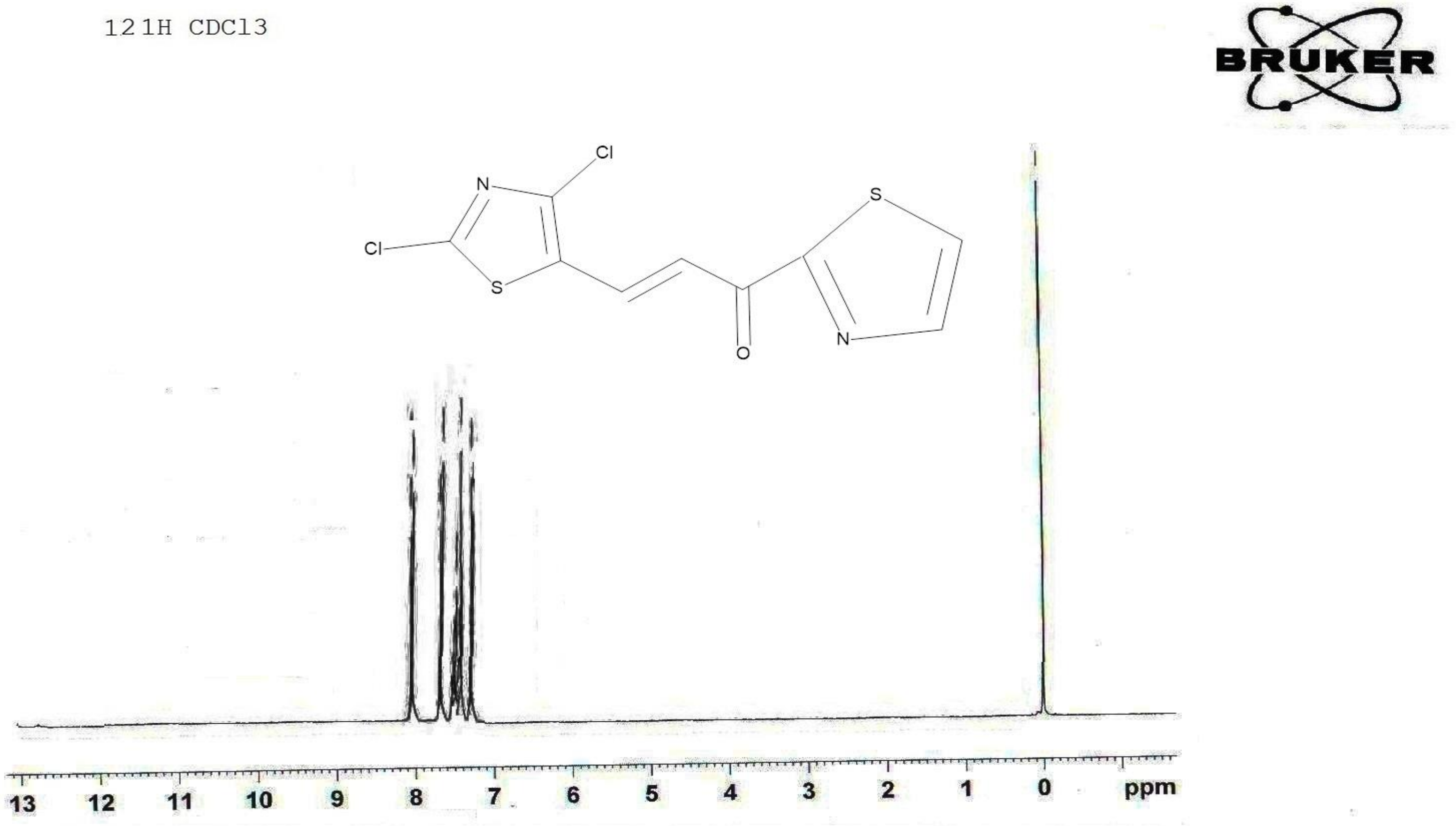

Figure S61. 1H NMR Spectrum of compound 20

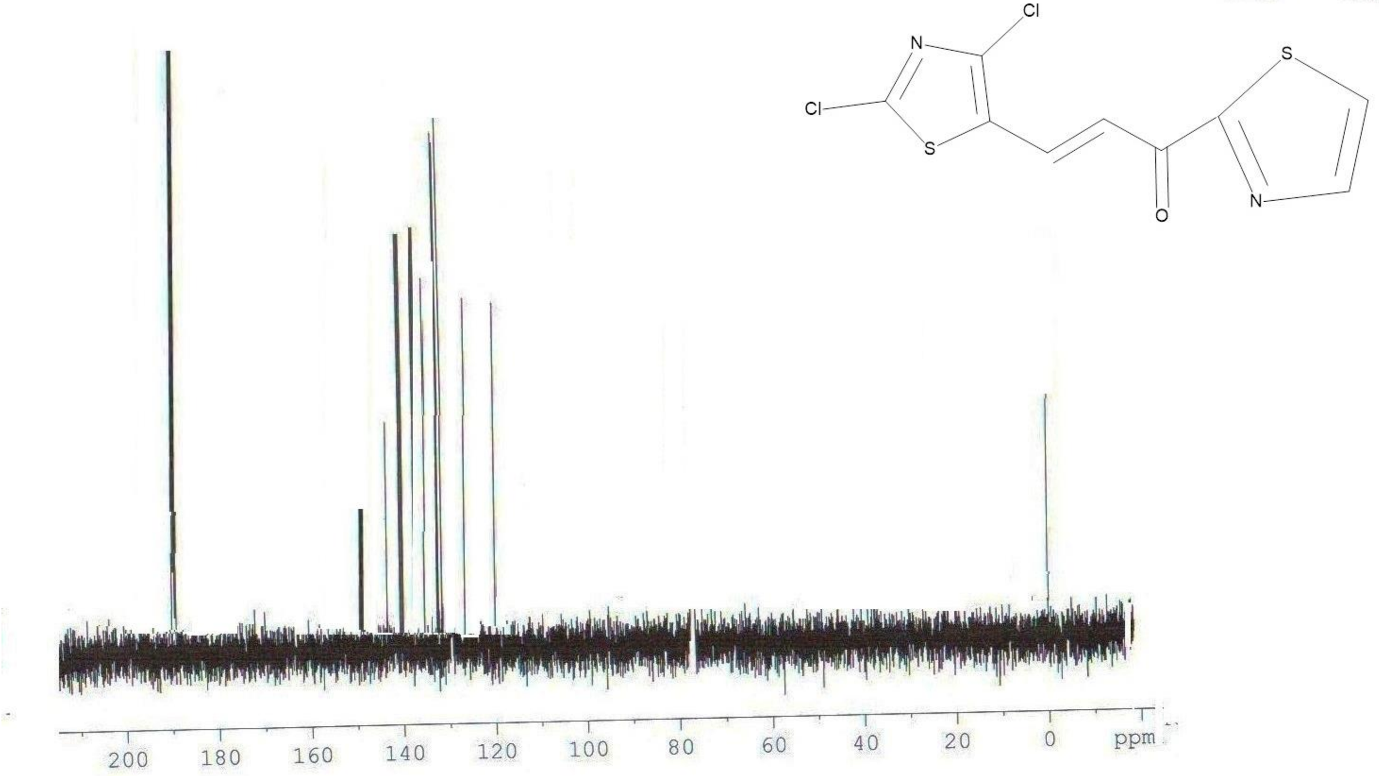

Figure S62. ^{13}C **NMR Spectrum of compound 20**

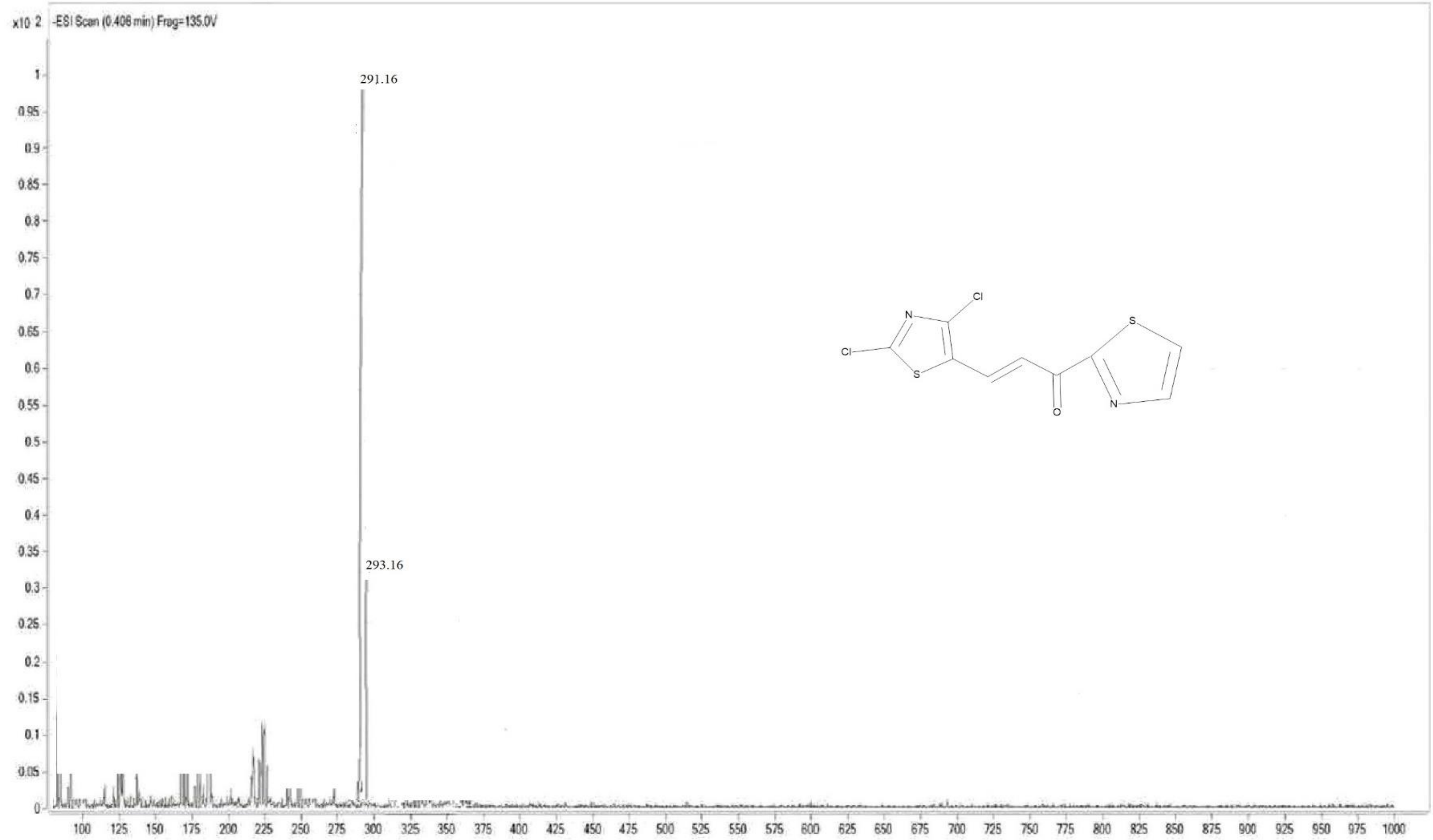

Figure S63. Mass Spectrum of compound 20
